# An extensive β_1_-adrenergic receptor gene signaling network regulates molecular remodeling in dilated cardiomyopathies

**DOI:** 10.1172/jci.insight.169720

**Published:** 2023-08-22

**Authors:** Philip D. Tatman, David P. Kao, Kathryn C. Chatfield, Ian A. Carroll, Jessica A. Wagner, Eric R. Jonas, Carmen C. Sucharov, J. David Port, Brian D. Lowes, Wayne A. Minobe, Sophia P. Huebler, Anis Karimpour-Fard, Erin M. Rodriguez, Stephen B. Liggett, Michael R. Bristow

**Affiliations:** 1 Division of Cardiology, Department of Medicine, and; 2 Colorado Center for Personalized Medicine University of Colorado School of Medicine, Aurora, Colorado, USA.; 3 Department of Pediatric Cardiology, Children’s Hospital Colorado, Aurora, Colorado, USA.; 4 ARCA biopharma, Westminster, Colorado, USA.; 5 Division of Cardiovascular Medicine, University of Nebraska Medical Center, Omaha, Nebraska, USA.; 6 Department of Biomedical Informatics, University of Colorado School of Medicine, Aurora, Colorado, USA.; 7 Departments of Medicine and Molecular Pharmacology and Physiology, Morsani College of Medicine, University of South Florida, Tampa, Florida, USA.

**Keywords:** Cardiology, Genetics, Bioinformatics, Heart failure, Molecular genetics

## Abstract

We investigated the extent, biologic characterization, phenotypic specificity, and possible regulation of a β_1_-adrenergic receptor–linked (β_1_-AR–linked) gene signaling network (β_1_-GSN) involved in left ventricular (LV) eccentric pathologic remodeling. A 430-member β_1_-GSN was identified by mRNA expression in transgenic mice overexpressing human β_1_-ARs or from literature curation, which exhibited opposite directional behavior in interventricular septum endomyocardial biopsies taken from patients with beta-blocker–treated, reverse remodeled dilated cardiomyopathies. With reverse remodeling, the major biologic categories and percentage of the dominant directional change were as follows: metabolic (19.3%, 81% upregulated); gene regulation (14.9%, 78% upregulated); extracellular matrix/fibrosis (9.1%, 92% downregulated); and cell homeostasis (13.3%, 60% upregulated). Regarding the comparison of β_1_-GSN categories with expression from 19,243 nonnetwork genes, phenotypic selection for major β_1_-GSN categories was exhibited for LV end systolic volume (contractility measure), ejection fraction (remodeling index), and pulmonary wedge pressure (wall tension surrogate), beginning at 3 months and persisting to study completion at 12 months. In addition, 121 lncRNAs were identified as possibly involved in *cis*-acting regulation of β_1_-GSN members. We conclude that an extensive 430-member gene network downstream from the β_1_-AR is involved in pathologic ventricular remodeling, with metabolic genes as the most prevalent category.

## Graphical abstract

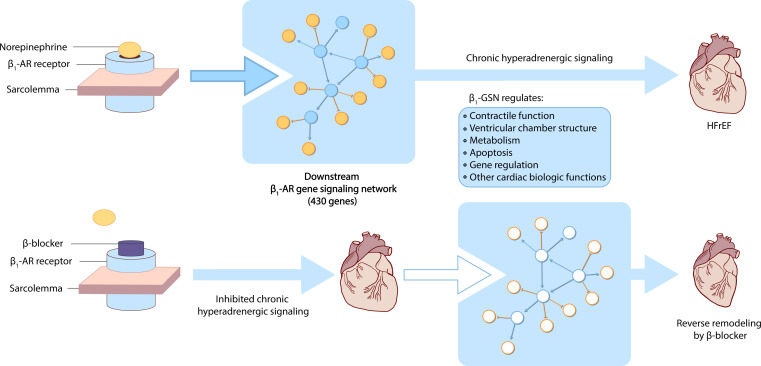

## Introduction

In patients with heart failure who have a reduced left ventricular (LV) ejection fraction (HFrEF), competitive inhibition of β_1_-adrenergic receptors (β_1_-ARs) by beta-blockers (BBs) produces the largest reduction in mortality among cornerstone drug therapies, thought largely to be due to favorable effects on pathologic ventricular remodeling (1, 2). The 2- to 3-month time course for detection of reverse remodeling (RR) indicates a biologic mechanism, presumably mediated by alterations in gene expression (1–3). Given the primary mechanism of action of BBs is to competitively inhibit β_1_-ARs, it is likely that a substantial portion of the gene expression changes associated with RR are the result of inhibition of chronic hyperadrenergic signaling with β _1_-ARs acting as a key node. This hypothesis is supported by work in the intact failing human heart (1–3), transgenic (Tg) mice overexpressing human β_1_-ARs (4, 5), cultured cardiac myocyte exposure to β-agonists (6), and isoproterenol infusions in murine models (7). However, BB-associated RR in dilated cardiomyopathies causes numerous gene expression changes (8, 9) that are unlikely to be primarily related to β_1_-AR signaling, considering the relatively small number of candidate genes identified as β_1_-AR regulated (3). Furthermore, the time course of gene expression changes and myocardial phenotypic associations has not been previously investigated. In addition, how such a β_1_-AR–driven gene network is regulated or coordinated has not been established.

Cardiac myocyte–targeted Tg β_1_-AR overexpression studies in mice have identified thousands of regulated genes that ultimately are associated with effects on LV performance and structure (4, 5). In the current study, we used a multispecies approach that included mouse and human models to identify data supporting an extensive, conserved β_1_-AR–responsive gene signaling network (β_1_-GSN) and its potential regulation in human ventricular myocardium. We propose that this network plays a major role in HFrEF pathophysiology and the BB therapeutic response and that it adds another dimension to the canonical role of β-adrenergic regulation of cardiac function.

## Results

In order to provide firm evidence that changes in mRNA expression associated with LV RR in BB-treated patients with dilated cardiomyopathy were from genes regulated by β_1_-AR signaling, we used *ADRB1* Arg389 and Gly389 cardiac overexpressor Tg mice (4, 5) as a biologic filter. In addition, we supplemented these identified genes by curating published information describing changes in myocardial or cardiac myocyte mRNA or protein expression associated with β-agonist or β-antagonist administration. We then matched these biologic filter gene expression changes to those associated with RR occurring in response to BB treatment (Table 1; Supplemental Methods; supplemental material available online with this article; https://doi.org/10.1172/jci.insight.169720DS1), with the requirement that the Tg mRNA or curated mRNA or protein abundance directional change be pharmacologically consistent with the RR change being a consequence of β_1_-AR blockade (Supplemental Methods, Supplemental Table 1).

### Tg ADRB1 cardiac overexpressor mice referenced to expression in reverse remodeled human left ventricles

In 3-month TG1 ADRB1 overexpressors (4), 7,085 genes had mRNA abundance comparisons to non-Tg (NTg) controls that were P < 0.05 in both Arg389 and Gly389 mice. Following Benjamini-Hochberg adjustment, 1,569 gene changes remained P < 0.05, with 370 upregulated and 1,199 downregulated (Supplemental Table 2A; individual genes listed in Supplemental Table 3). In 3 or 6–8 month TG2 ADRB1 overexpressors (5), there were 837 genes with ≥ 2 P < 0.05 differences versus NTg controls in Arg389 at 3 months, Gly389 at 3 months, or Gly389 at 6–8 months (434 upregulated and 403 downregulated; Supplemental Table 2A; individual genes are listed in Supplemental Table 3B). Between TG1 and TG2, 2,406 candidate genes were identified and 214 were present in both cohorts, resulting in 2,192 distinct genes and a concordance rate of 17.8% (Supplemental Table 2A and Supplemental Table 3C).

In RR responder interventricular septum biopsied from patients nonischemic dilated cardiomyopathy (NDC), there were 6,909 genes with responder versus nonresponder changes in expression, to which Tg mouse mRNA expression was compared. In TG1, there were 225 Tg genes with expression changes opposite to RR responder versus nonresponder changes, or 14.3% of the Tg total changes (Supplemental Table 2A). In human RR LVs, 61 of these genes were downregulated while upregulated in TG1 mice, and 164 were upregulated coupled with downregulation in TG1 (Supplemental Table 2A). For TG2, a total of 243 genes were antithetically changed in RR, or 29.0% of the 837 total changes (Supplemental Table 2A). Of these, 63 (14 downregulated and 49 upregulated) were also present in TG1. Therefore, for pharmacologically concordant changes, 28.0% of TG1’s and 25.9% of TG2’s gene expression changes were shared with the counterpart Tg mouse group. The total number of individual genes with pharmacologically concordant significant expression changes across all Tg mouse models and RR was 405 (176 upregulated and 229 downregulated, Supplemental Table 2A).

### Literature curation

There were an additional 25 curated genes with expression changes opposite to those in RR human hearts that were not identified as changed under ≥ 2 experimental conditions in Tg mice (Supplemental Table 2B and Supplemental Table 4). All the curated genes were present on the mouse array, and 17 were identified by same-direction changes in at least 2 independent studies, while 8 were through the combination of 1 published study and a single change in the Tg β1-AR overexpressors (Supplemental Table 4). These 14 downregulated and 11 upregulated genes were then added to the 405 from the Tg mouse data, resulting in a total β1-GSN population of 430 genes — 190 downregulated and 240 upregulated in RR LVs (Supplemental Table 2C).

### Gene expression changes from baseline to last observation in patients with dilated cardiomyopathy

The number of genes with *P* < 0.05 mRNA abundance changes from baseline in NDC RR responders versus nonresponders at last observation carried forward (LOCF, *n* = 39 at 12 months, 8 at 3 months) is given in Supplemental Table 5. With the 3 platforms used to measure mRNA abundance in 2 different cohorts, by at least 1 platform measurement, there were 2,975 unique genes upregulated and 3,934 downregulated. Concordance between at least 2 platforms was 6.5% for upregulated and 8.2% for downregulated genes. Compared with the total number of changed expression genes, concordance was markedly higher in the 430 β_1_-GSN genes, 25% in the 240 upregulated genes (*P* <0.0001), and 35% in the 190 downregulated genes (*P* < 0.0001) (Supplemental Table 5). For the Any Platform (RT-PCR, microarray, or RNA-Seq) analysis, downregulated genes outnumbered upregulated (*P* = 0.016), but upregulated were more numerous than downregulated (*P* = 0.025) in the β_1_-GSN (Supplemental Table 5).

### Gene ontology using general classification tools

**Figure 1:**
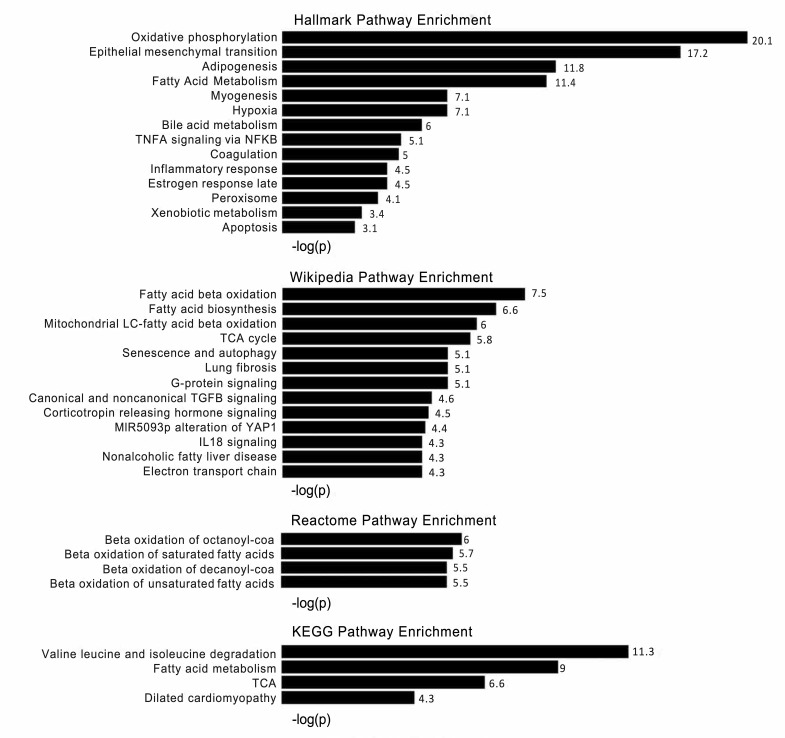
Canonical pathway enrichment of the β_1_-GSN in reverse-remodeled human left ventricles. –Log *P* values at the end of bars are from a modified Fisher’s exact test to determine pathway enrichment, with a Benjamini-Hochberg correction for FDR. *n* = 47.

**Figure 2:**
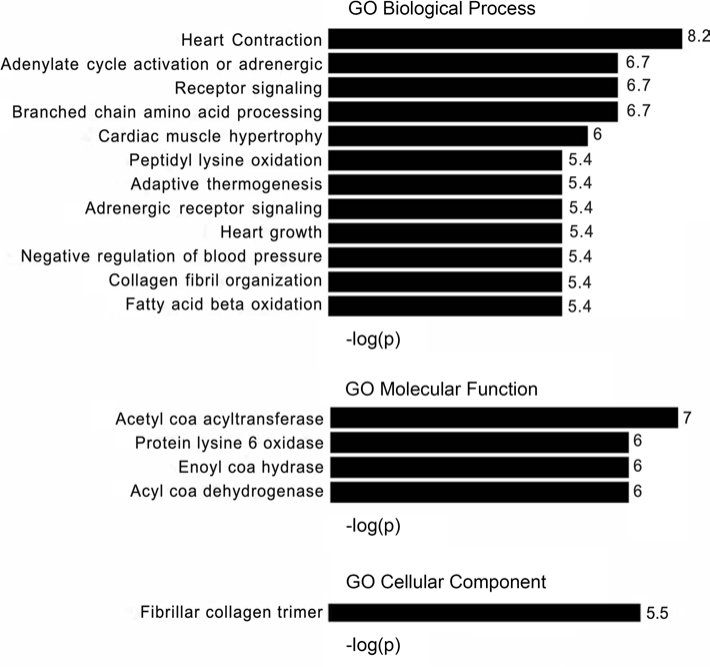
GO pathway enrichment of the β_1_ gene signaling network in reverse-remodeled human left ventricles. –Log *P* values at the end of bars are from a modified Fisher’s exact test to determine pathway enrichment, with a Benjamini-Hochberg correction for FDR. *n* = 47.

To investigate biologic function, we performed a gene ontology analysis on the 430 β_1_-GSN RR genes (Figures 1 and 2), including separate analyses with the 190 downregulated (Supplemental Methods, Supplemental Figures 1 and 2) and 240 upregulated genes (Supplemental Figures 3 and 4). The -log scores of the enrichments of the β_1_-GSN genes versus the entire human genome were displayed as waterfall plots for all pathways reaching a P < 0.05 after correcting for multiple comparisons. All 3 analyses (whole network, downregulated, and upregulated genes) revealed significant enrichments in some known cardiac pathologies of contractile, metabolic, immune, and extracellular matrix (ECM) pathways. The mechanistic diversity is easily visualized by a Reactome pathway map (Supplemental Figure 5). Despite these findings, the canonical and GO databases only cover 249 and 264 β_1_-GSN–responsive genes, respectively, and when combined, only 316 of the 430 total β_1_-GSN genes.

### Human ventricular myocardial ontology

With approximately 27% of β_1_-GSN–responsive genes lacking annotation and much of the network lacking a cardiac-specific assignment, we utilized a biologic classification developed specifically for pathologic eccentric ventricular remodeling in the human left ventricle (9). With this ventricular myocardial ontology (VMO) framework, 96% of the β_1_-GSN–responsive genes were assigned to specific biologic categories (Table 2).

### Cardiac myocyte contractile or pump function

One of the 2 components of pathologic eccentric remodeling that directly characterizes HFrEF is systolic contractile dysfunction (2). There were multiple mRNA expression changes of β_1_-GSN genes whose encoded proteins affect cardiac myocyte or chamber contractile function. In this group, there was no difference in upregulated versus downregulated genes (Table 2). However, changes in the apoptosis and contractile and associated protein categories would be expected to improve chamber contractile function (Table 2, Table 3, Supplemental Table 6, and Supplemental Figure 6A).

#### Ca^2+^ handling.

The SR calcium ATPase Serca 2a (*ATP2A2*) and the gene for its major regulator, phospholamban (*PLN*), as well as the ryanodine release channel (*RyR2*) were upregulated with RR, as previously described (3, 9) (Table 3, Supplemental Table 6, and Supplemental Figure 6A). Also upregulated was *S100A1*, whose encoded protein can affect Ca^2+^ handling and is a positive regulator of contractility (10), and *ASPH*, whose encoded product is important for SR Ca^2+^ homeostasis (11). Downregulated genes included *SLC8A1* — which encodes the Na^+^/Ca^2+^ exchanger (NCX) gene that is upregulated in the failing heart and in response to isoproterenol treatment (12) — and calnexin (*CANX*), encoding an ER associated molecular chaperone that is upregulated in the failing heart (13). Also downregulated were the gene for the Ca^2+^ binding protein S100A11, and *SYT12,* whose encoded protein is involved in Ca^2+^ mediation of neurotransmitter release (Table 3, Supplemental Table 6, and Supplemental Figure 6A).

#### β-Adrenergic/cAMP/PKA signaling.

In the canonical β_1_-AR cAMP/PKA pathway, 7 genes were upregulated in RR (Table 3, Supplemental Table 6, and Figure 6A), with 3 expected to lead to enhanced (*ADCY9*, *ADRB1*, *ADRB2*) and 3 to diminished (*PKIA*, *PPP1R3B*, *PPP1R1A*) cAMP production and PKA signaling. The seventh upregulated gene (*CDNF*) encodes a dopamine neurotrophic factor that also prevents apoptosis in H9c2 cells (14). With the exception of *GNB1,* the encoded proteins of the 10 downregulated genes (Table 3, Supplemental Table 6, and Supplemental Figure 6A) would be associated with increased cAMP/PKA signaling via decreases in phosphodiesterases (*PDE10A*, *PDE3B*, *PDE4B*, *PDE8A*), phosphatases (*PPP1R3C*, *PPP1R14B*), G protein receptor kinases (*GRK3*, *GRK5*), and inhibitory G protein subunit ai2 (*GNAI2*). *PDE10A* deserves particular emphasis, since its increased expression mediates pathologic but not physiologic hypertrophy and inhibition; by extension, downregulation can reverse pathologic ventricular remodeling in model systems (15).

#### Contractile and associated proteins.

As previously reported (3, 6, 9, 16), *MYH6*, which encodes the higher ATPase activity isoform of myosin heavy chain, meets criteria as a β_1_-GSN member and was upregulated in RR (Table 3, Supplemental Table 6, and Supplemental Figure 6A). *MYL3*, essential ventricular light chain 1, encoding a protein associated with an increased LV contractile state in isolated rat hearts (17), was similarly upregulated with RR. Also upregulated was myosin light chain kinase 4 (*MYLK4*), encoding a Ca^2+^/calmodulin–independent light chain kinase that phosphorylates ventricular regulatory light chain resulting in an increase in contractility (18), the striated muscle ubiquitin ligase gene *TRIM63*, and the muscle stress transducer gene *LRRC39*. Only 1 contractile protein gene was downregulated, the skeletal muscle isoform of α-actin (*ACTA1*). This isoform is expressed in fetal development in rodents and is upregulated in the failing human heart to become the majority isoform, but in isolated in vitro motility experiments, it is not associated with contractility differences compared with the cardiac isoform (19) (Table 3 and Supplemental Table 6).

#### Apoptosis.

Seven genes with encoded proteins that can facilitate apoptosis were downregulated with RR (Table 3, Supplemental Table 6, and Supplemental Figure 6A), including *CASP3* and *DAP*. The other 5 (*RERE*, *TM2D2*, *FILIP1L*, *MIF*, *PHLDA3*) play various roles in biologic processes that can lead to apoptosis. The only upregulated gene in this category was *TRIAP1*, which encodes an apoptosis inhibitor (20).

### Pathologic chamber hypertrophy

The second major component of eccentric pathologic chamber remodeling is hypertrophy, for which the VMO classification has 4 categories (Table 3 and Figure 6A). In these 4 categories combined, downregulated genes outnumbered upregulated 56 to 13 (*P* <0.0001) (Table 3 and Supplemental Table 6).

#### Growth/hypertrophy.

With RR, the growth/hypertrophy category exhibited an excess in downregulated genes, 13 versus 4 upregulated (*P* = 0.029) (Table 3). Notable known mediators of hypertrophy were downregulated, including *IGF1*, the translation regulator *IGF2BP2*, *EDN1*, and the hypertrophy biomarker natriuretic peptides *NPPA* and *NPPB*. Also downregulated were *MUSTN1*, which encodes a myogenesis factor, and 2 genes encoding regulators of cell growth, *GRN* and *GPC1*. *TMEM43* encodes a protein necessary for normal ventricular contractile protein content, and *EXT1* encodes an ER enzyme responsible for chain elongation of heparan sulfate, a cardiac myocyte growth regulator. Two of the downregulated genes, *RCAN1* (encoding a calcineurin inhibitor) and *BTG1* encode antiproliferative proteins, indicating that downregulation of genes in this category doesn’t transfer 1:1 to hypertrophy regression (Table 3 and Supplemental Table 6).

Four genes were upregulated with RR in the growth/hypertrophy category, and 2 of them (*MTSS1* and *SESN1*) encode growth inhibitors (Table 3 and Supplemental Table 6). The other 2 (*KY* and *GAB1*) encode proteins involved in normal cell growth responses; in mice, *GAB1* gene ablation causes a dilated cardiomyopathy and mitochondrial damage (21).

#### Cytoskeleton.

Five genes assigned to the cytoskeleton category were downregulated with RR, including 2 RAC1 regulators (*PAK3* and *PAK4*) whose enzyme gene products link this GTPase to cytoskeletal reorganization and regulation (Table 3 and Supplemental Table 6). In this group, there are also 2 genes (*TWF1*, *DBN1*) encoding actin binding proteins and a *FHL1* encoding a LIM-only protein expressed in fetal development. The only upregulated gene in the cytoskeleton category was *LDB3*, which encodes a LIM domain binding protein that interacts with α-actinin-2.

#### Fibrosis promoting, ECM.

In RR, changes in mRNA expression of genes encoding fibrosis or ECM promoting proteins were markedly biased toward downregulation, *n* = 36 versus only 3 with upregulation (*P* <0.0001; Table 3 and Supplemental Table 6). Downregulated genes included those encoding 7 collagens (*COL1A1*, *1A2*, *3A1*, *5A1*, *6A3*, *14A1*, *16A1*), elastin (*ELN*), 4 associated with collagen processing (*P3H2*, *ADAMTS2*, *PLOD3*, *BGN*), 7 other ECM related genes (*TNC*, *SPARC*, *ASPN*, *NID1*, *MGP*, *FBN1*, *CCDC80*), 3 matrix metallopeptidases (*MMP2*, *-14*, *-19*) and 1 inhibitor (*TIMP1*), 4 lysl oxidases (*LOX*, *LOXL-1*, *-2*, *-4*), 4 associated with TGF-β signaling (*TGFB1*, *GDF11*, *SMAD7*, *BAMBI*), 3 associated with fibroblast regulation (*PI16*, *UCHL1*, *DUSP5*), fibronectin (*FN1*), and the fibrosis biomarker galectin (*LGALS1*) (Table 3 and Supplemental Table 6). With RR, decreased protein expression of these genes would be expected to reduce myocardial fibrosis and other ECM deposition that is increased with β_1_-GSN signaling in TG1 (4) and TG2 (5) LVs and was also present at baseline in the investigated patient population (3). Only 1 of the 3 upregulated genes in this category, *PCOLCE2*, has any evidence that its encoded protein can participate in myocardial fibrosis enhancement.

#### Microtubule function/integrity.

Microtubules are assigned a separate subcategory from cytoskeleton because of their known regulation by β_1_-AR signaling and their potential to modify contractile function (22). There were 5 upregulated and 2 downregulated genes (*P* = 0.26), and this upregulation trend differed from the downregulation in the other 3 Pathologic Chamber Hypertrophy subcategories (2 × 2 χ^2^ *P* = 0.0002; Table 3 and Supplemental Table 6). Of the upregulated genes, 2 (*DPYSL2*, *MAPT*) encode proteins that promote microtubule assembly, 2 (*FSD2*, *CEP85*) protein products are involved in microtubule organization, and 1 (*FYCO1*) encoded protein participates in autophagic vesicular transport. Of the 2 downregulated genes, *MAP1LC3A* encodes a light chain subunit of microtubule-associated protein 1A or 1B that mediates cytoskeletal interactions, and the 1B gene (*MAP1B*) was itself downregulated.

### Metabolism

#### Mitochondrial based functions.

The number of changes in metabolic genes associated with RR is striking, and the majority are in the mitochondrial upregulation subcategory (47 of 67 upregulation and 53 of 83 total changes, both *P* <0.0001; Table 2, Supplemental Table 7, and Supplemental Figure 6B). Of the 47 upregulated mitochondrial genes, 46 encode proteins involved in either fatty acid transport, utilization, or β-oxidation (*n* = 15); branched chain amino acid metabolism (*n* = 4); glucose metabolism (termination of glycolysis, *n* = 6); the TCA cycle (*n* = 7); or respiratory chain and cofactor synthesis (electron transport chain, *n* = 15) (Supplemental Table 7).

#### Fatty acid metabolism, including β-oxidation.

There were 12 upregulated genes whose encoded proteins are critically involved in mitochondrial fatty acid β-oxidation, and none that were downregulated (*P* <0.0001; Supplemental Table 7 and Supplemental Figure 6B). These include genes encoding proteins involved in long-chain fatty acid β-oxidation, *CPT1B*, *ACADVL*, *HADHA*, *HADHB*, and *ACAA2*. There is also evidence for increased utilization of medium-chain fatty acids, with upregulation of *ACADM*. Other upregulated genes encoding enzymes that participate in fatty acid β-oxidation were *DECRI* and *ECI1*. Mitochondrial acyl-CoA dehydrogenases that utilize both long-chain fatty acids and branched-chain amino acids — *ACAD8*, *ACAD10*, *ACADSB*, and *HADH* — were also upregulated in RR. Beyond entry into and effectuation of β-oxidation, there were multiple other upregulated genes encoding proteins that facilitate fatty acid metabolism (*ACSL1*, *ECHDC2*, and *ECHDC3*; Supplemental Table 7). In addition to the 15 β_1_-GSN upregulated genes functioning in fatty acid metabolism, 4 other upregulated genes encode fatty acid transport or enzymatic activity in peroxisomes, and 1 (*CD36*) fatty acid transport in the sarcolemma (23). In all, 21 upregulated genes encode enzymes (*n* = 18) or transporters (*n* = 3) involved in fatty acid metabolism. In contrast, only 1 downregulated gene (*FADS1*; *P* < 0.0001 versus upregulated) encodes a protein involved in fatty acid metabolism.

#### Branched-chain amino acid metabolism.

Four genes encoding enzymes that degrade branched-chain amino acids to form acetyl CoA were upregulated. *AUH* and *MCCC1* are key enzymes in leucine degradation, and *HIBADH* and *BCKDHA* are involved in valine and branched-chain keto acid metabolism. There were no downregulated genes involved in metabolism of branched chain amino acids (*P* = 0.046; Supplemental Table 7).

#### Tricarboxylic acid cycle.

Of the remaining upregulated genes encoding proteins important for mitochondrial function, 4 are enzymes critical for the tricarboxylic acid (TCA) cycle: *CS*, *SUCLA2*, *IDH2*, and *IDH3B*. Also upregulated was *ACAT1*, which encodes a protein involved in cholesterol biosynthesis but can also regulate acetyl-CoA entry into the TCA cycle. Additionally, 2 upregulated genes involved in anaplerotic replenishment of succinyl-CoA are included in this group, *MUT* and *MMAB* (Supplemental Table 7). Thus, 7 TCA associated β_1_-GSN genes were upregulated with RR, versus none downregulated (*P* = 0.008).

#### Regulation of mitochondrial respiratory capacity (electron transport chain).

Of the 47 mitochondrial genes that were upregulated with RR, 15 encode proteins that reside in the electron transport chain (Supplemental Table 7). *NDUFS1,* *NDUFS2*, *NDUFS4*, *NDUFA5*, and *NDUFC2* are enzymatic subunits of respiratory chain complex I, *SDHA* and *SDHD* are 2 of the 4 enzymatic subunits of complex II, and *CYC1* encodes a subunit of the cytochrome bc1 complex (complex III). Genes encoding enzymes necessary for coenzyme-Q (ubiquinone) synthesis, including *COQ3* and *COQ9*, were upregulated, as was 1 subunit of complex IV — the *COX6C* subunit of cytochrome C oxidase. Electron transfer flavoprotein-ubiquinone oxidoreductase complex protein subunits *ETFB* and *ETFDH*, which are a critical link between the β-oxidation pathway and the respiratory chain, are also found in the upregulated group, as is *NUDT13*, which encodes an enzyme believed to be involved in regulation of mitochondrial NAD(P)^+^/NAD(P)H ratios, energy homeostasis, and redox control (24). Of additional interest is *GBAS* (also known as *NIPSNAP2*), which encodes a mitochondria-associated protein suspected to play a role in mitochondrial respiration (25).

#### Mitochondria- and cytosol-based functions — glycolysis.

With RR, genes encoding PDH subunit B (*PDHB*) and the E2 subunit (*DLAT*) were upregulated (Supplemental Table 6A, Supplemental Table 7, and Supplemental Figure 6B). However, *PDK2* was also upregulated; its encoded protein reversibly inactivates the PDH complex via phosphorylation of the E1 subunit. Further complicating the interpretation of inferred PDH activity is the fact that the gene whose encoded phosphatase dephosphorylates the E1 subunit, *PDP1*, was upregulated in RR. In addition, expression of *PFKM*, whose encoded protein is the rate-limiting step in glycolysis, was upregulated in RR while the “platelet” isoform, *PFKP*, was downregulated. Also downregulated was *HK1*, primarily expressed in mitochondria but also in the cytosol, whose encoded enzyme along with HK2 catalyze the phosphorylation of intracellularly transported glucose to glucose-6-phosphate (Supplemental Table 6B, Supplemental Table 7, and Supplemental Figure 6B). *PGAM1* was another downregulated cytoplasmic glycolysis enzyme gene. *PANK4*, whose encoded protein can interact with PDK2 (26), is also included in the glycolysis subcategory. Therefore, of the 6 upregulated genes encoding constituents of glycolysis, gene products of 5 were mitochondria based and involved in the terminal phase of pyruvate dehydrogenase activity or regulation. In contrast, of the 3 downregulated glycolysis genes, 2 encoded enzymes localized in the cytosol (*PFKP* and *PGAM1*), and 1 was expressed in both mitochondria and cytosol (*HK1*) (Supplemental Table 6B and Supplemental Table 7).

#### Peroxisome-based functions.

Very long–chain fatty acids are metabolized in the peroxisome, and *ABCD2*, a gene encoding a critical peroxisomal enzyme responsible for transmembrane import of fatty acids, was upregulated in RR (Supplemental Table 7 and Supplemental Figure 6B). Also upregulated in the peroxisomal compartment was *PHYH*, whose encoded protein catalyzes the first step in α-oxidation of the branched-chain fatty acid phytanic acid; *SCP2*, encoding a protein involved in the oxidation of branched chain fatty acids; and *ECHI* whose encoded protein functions in the auxiliary step of the β-oxidation pathway (Supplemental Table 7 and Supplemental Figure 6B). There were no downregulated genes whose encoded proteins are peroxisome based.

#### Sarcolemma, sarcoplasmic reticulum, or endoplasmic reticulum genes with metabolism functions.

Of potential particular interest is the marked (by 12.6-fold) upregulation of *DHRS7C* (Supplemental Table 6A, Supplemental Table 7, and Supplemental Figure 6B), which encodes a short-chain dehydrogenase/reductase that is highly expressed in the endoplasmic reticulum (ER) of cardiac myocytes. *DHRS7C* gene expression has been previously shown to be downregulated by both β- and α_1_-agonists, as well as in HF (27).

### Gene regulation, transcription, and translation

This category, which encompasses any process affecting protein gene product formation, was markedly biased toward upregulation in RR, 50 versus 14 downregulated (*P* <0.0001; Table 2). The gene regulation subcategory membership is listed in Supplemental Tables 6 and 8.

#### Transcription.

The excess numbers of genes exhibiting upregulation versus downregulation are in the transcriptional regulation (15 versus 3, *P* = 0.005), transcription factor (TF; 7 versus 2, *P* = 0. 096), mRNA processing or stability (8 versus 0, *P* = 0.005), ribosomes (5 versus 1, *P* = 0.10), and DNA repair, stability, or synthesis (6 versus 1, *P* = 0.059) subcategories (Supplemental Table 8). Transcription regulation is represented by upregulation in multiple repressors (*RBL2*, *RXRG*, *SAFB2*, *THUMPD1*, *KLF9*, *HDAC4*, *RCOR2*, *NR1D2*, *JARID2*) and fewer enhancers or activators (*RFXAP*, *HLF*). However, 1 of the 3 downregulated transcriptional regulators is *NELFE*, whose encoded protein represses RNA polymerase II (Pol II) transcript elongation. In addition, multiple other expression changes in genes regulating Pol II favor increased activity, including upregulation of *TCEA3* (encoding a cofactor in Pol II elongation), *MED4* (encoding a component of the Mediator complex), and *CDK19* (encoding a cofactor for Mediator and PCF11 polyadenylation factor subunit)*,* involved in Pol II transcription termination).

#### TFs.

Among upregulated TFs was *CREBZF* (Supplemental Table 8), encoding a protein that binds cooperatively with ATF4 to transactivate cAMP response elements when activated by PKA phosphorylation (28). Cardiac overexpression of a dominant negative CREB/ATF bZIP construct produces dilated cardiomyopathy in mice (29). The downregulated gene *NR4A1* encodes an orphan nuclear receptor TF that is inducible by isoproterenol and regulates the expression of multiple cardiac metabolic genes (30). Also downregulated was the nuclear receptor coactivator *PNRC1*, whose encoded protein facilitates activation of multiple nuclear hormone receptors (31).

#### mRNA processing or stability.

Eight genes that encode mRNA processing proteins were upregulated in RR (Supplemental Table 8), versus none downregulated (*P* = 0.005). These included 4 heterogenous nuclear riboproteins that bind to pre-mRNAs in the nucleus (*HNRNPA1*, *HNRNPA2B1*, *HNRNPM*, and *HNRPDL*).

#### mRNA splicing.

Two genes were upregulated, *RBM20* and *RBFOX1*, with *SRPK2* downregulated (Supplemental Table 8).

#### Ribosomes.

Ribosomal protein gene expression exhibited a nonsignificant trend for more upregulated genes (Supplemental Table 8, 5 versus 1 downregulated, *P* = 0.10), including 3 mitochondrial ribosomal protein genes (*MRPL9*, *MRPS23*, *MRPS9*) versus none downregulated.

#### DNA repair, stability and synthesis.

The DNA repair, stability and synthesis category is biased toward upregulated genes, 6 versus 1 downregulated (*P* = 0.059, Supplemental Table 8) and included 2 whose encoded proteins target mitochondrial DNA (*REVL3* and *RRM2B*).

#### Chromatin/histones.

Of potential importance for gene network coordination, the bromodomain and extraterminal (BET) family protein *BRD4*, a nodal effector of pathologic myocardial remodeling (32) via superenhancer binding and chromatin regulation, was downregulated (Supplemental Table 8).

#### DNA methylation, other epigenetic regulation.

In this subcategory, the DNA methyltranferase *N6AMT1* and lysine methyltransferase *N6AMT2* (*EEF1AKMT1*) genes were upregulated in RR, and *CDK2AP1* was downregulated (Supplemental Table 8).

### Channels and solute exchangers

Channels and solute exchangers exhibited 12 upregulated and 4 downregulated genes (*P* = 0.046; Table 2). The downregulated genes include *SLC9A1*, encoding a Na^+^/H^+^ exchanger (Supplemental Table 6 and Supplemental Table 9A) that is coupled to hypertrophy in β_1_-AR overexpressor mice (33). Among upregulated genes were 2 encoding potassium channels (*KCND3*, *KCNJ11*) and 1 (*RNF207*) that encodes a protein that stabilizes expression of KCNH2 (HERG) channels.

### Other changes within VMO categories

None of the remaining categories in Table 2 exhibited statistically significant differences in up- or downregulation by 1 × 2 χ^2^ test or had component changes that are likely directly related to pathologic remodeling. These categories and their gene membership are described in Supplemental Methods, Supplemental Table 6, Supplemental Table 9B, and Supplemental Table 10.

#### Relationships of β_1_-GSN gene expression to phenotypic characteristics.

Microarray global gene expression measurements were available at months 0, 3 (46 subjects each), and 12 (39 subjects), enabling a temporal assessment of β_1_-GSN VMO category gene expression to RR phenotypic measures as overviewed in Supplemental Figure 7. The selected phenotypic characteristics were measures of ventricular structure (LV EDV); structure plus function (remodeling, LV ejection fraction [LVEF], right ventricular ejection fraction [RVEF]); ventricular systolic/contractile function (LV ESV); ventricular filling pressures (pulmonary wedge pressure [PWP], right atrial mean pressure [RAP]); clinical HF (New York Heart functional class [NYHA] functional class, plasma norepinephrine [NE]); pharmacodynamic effects of β-blockade (heart rate [HR], systolic blood pressure [SBP]); and clinical measures not directly related to HF (creatinine clearance [CrCl], BMI) (Table 1 and Supplemental Table 11). In responders as well as the entire cohort (EC), ESV, LVEF, and RVEF were favorably changed at 3 months and then progressively improved at 12 months, with highly statistically significant linear trend tests. EDV exhibited the same pattern. Reduction in PWP reached statistical significance in responders at 12 months with a significant linear trend, but RAP was not changed. NYHA class exhibited a marked improvement at 12 months, with 31% of responders and 26% of the EC becoming asymptomatic, plus a shift from NYHA class III to II at 3 months in both groups. NE was not significantly changed in responders or the EC. HR exhibited the expected reduction from β-blockade at both 3 and 12 months in responders and the EC but, interestingly, was unchanged in nonresponders who treated with the same target doses of β-blockade (3). SBP exhibited a paradoxical small increase at both 3 and 12 months in responders and the EC, which is likely due to improved systolic function. BMI increases slightly at both 3 and 12 months in responders and the EC, while CrCl was unchanged in all groups. In contrast, in nonresponders, no linear trend tests were positive, and only 2, NYHA at 3 months and BMI at 12 months, were statistically significant compared with month 0.

Relationships between mRNA abundance and the phenotypic measures in Supplemental Table 11 were assessed by nonparametric permutation testing, generating a permuted *Z* score (*Z*_p_) that reflects the strength of β_1_-GSN VMO category mRNA abundance-phenotypic parameter relationship relative to the null. The key component of the *Z*_p_ is the within-VMO category averaged Rho value across all the gene members. To illustrate the type of correlations produced, Supplemental Figure 8 is a plot of the average normalized mRNA abundance for metabolism genes versus LVEF or PWP in the 46 study subjects, generating a composite Rho in contrast to the *Z*_p_ calculation, which is based on the average Rho of individual gene mRNA abundance-phenotype correlations. The characteristics of these plots are discussed further in Supplemental Methods.

**Figure 3:**
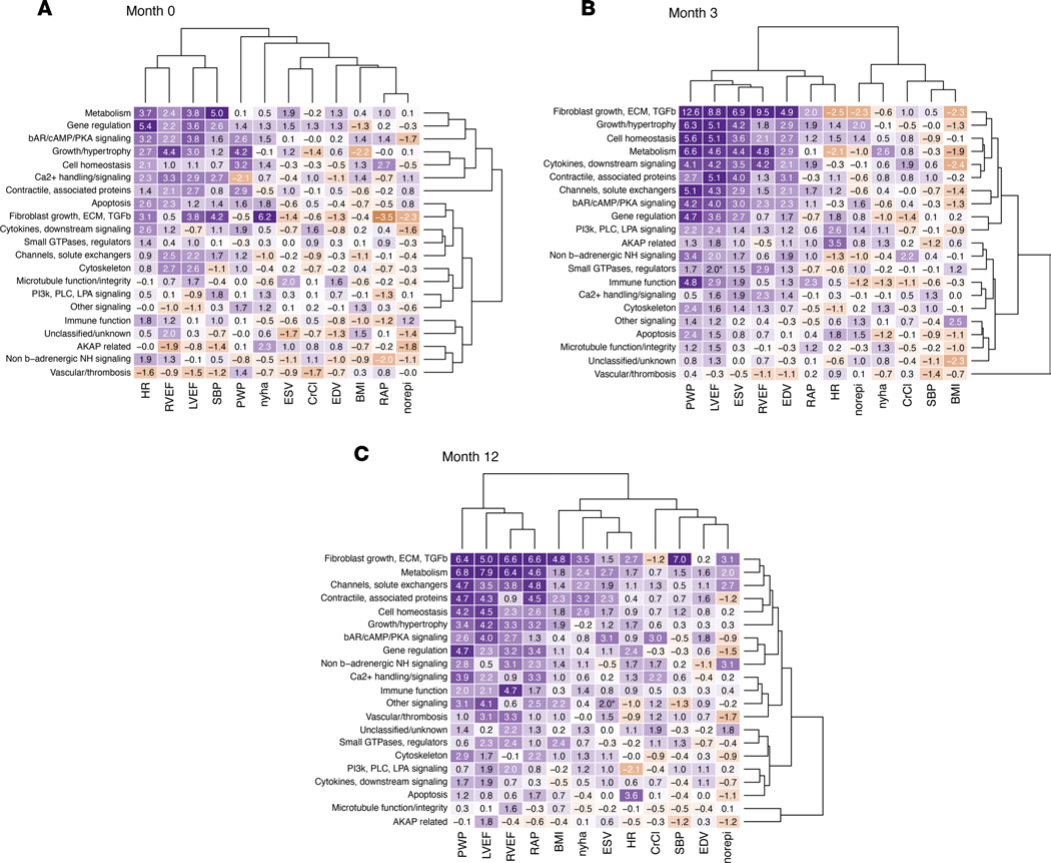
Heatmap and cluster analysis of the *Z*_p_ comparing β_1_-GSN mRNA abundance-phenotype correlations with non–β_1_-GSN controls. A positive or negative *Z*_p_ measures the degree of correlation that is respectively > or < controls/null, with an absolute *Z*_p_ of > 1.96 (rounded to 2.0) considered statistically significant. Blue color intensity is the degree that the β_1_-GSN *Z*_p_ exceeds the expected correlation in controls, red represents correlation less than expected, and white (0.0) is the null. The linear scale is suppressed at absolute values > 5.0. (**A**) Month 0 (baseline, *n* = 46), mRNA abundance of the 430 β_1_-GSN genes compared with values of 12 different cardiac and patient phenotypic characteristics in the 30 reverse remodeling responders. (**B** and **C**) Month 3 (*n* = 46) and month 12 (*n* = 39) are shown.

**Figure 4:**
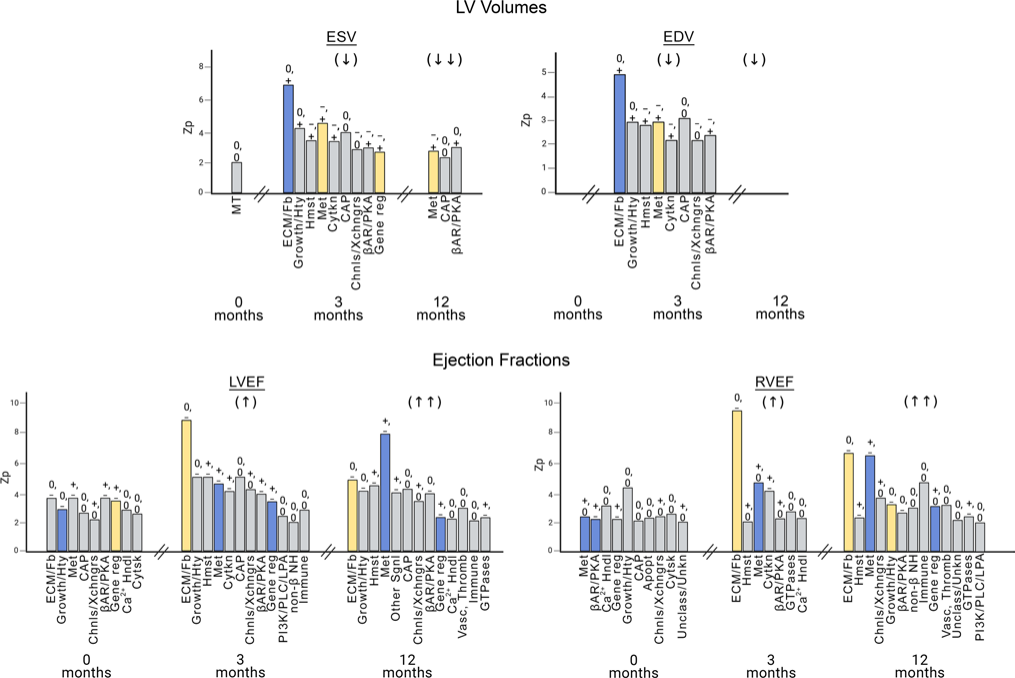
Temporal pattern of mRNA abundance-ventricular function and structure phenotypic relationships. Patients with nonischemic dilated cardiomyopathy were investigated at baseline (month 0; *n* = 46) on no beta-blocker treatment; *n*= 46 were investigated at 3 months and *n* = 39 at 12 months on beta-blockade. The *y* axes are *Z*_p_ values ≥ 1.96 from nonparametric permutation testing of average Spearman’s rank correlation Rho values of β_1_-GSN ventricular myocardial ontology (VMO) categories (Table 2 and Figure 3) versus non–β_1_-GSN VMO controls at months 0, 3, and 12. Blue, direct relationship of phenotypic measurement with net (including upregulated and downregulated genes) RNA expression; yellow, inverse relationship of net mRNA abundance changes with phenotypic measure; gray, no statistically significant relationship of net mRNA expression and phenotype measurement. The designations above the bars are as follows: first entry is for upregulated genes (top), second is for downregulated (bottom); (+), direct directional correlation with the phenotypic measure; (−), inverse directional correlation; 0, no directional correlation.

Heatmaps for mRNA abundance versus phenotypic measure *Z*_p_ values and cluster analyses for each of the 21 gene expression categories and 12 phenotypic characteristics for the 3 time points are in Figure 3. Data in Supplemental Table 11 indicate that the study population was composed of 2 populations of ventricular myocardial RR related responses, which are substantially favorable in the 30 responders and absent in the 16 nonresponders. The *Z*_p_ in the heatmap are the average of Spearman’s rank correlation coefficients (Rho) in the β_1_-GSN ontology categories versus each phenotypic measure, standardized using control values from non β_1_-GSN genes. At month 0 (Figure 3A), for the 430 β_1_-GSN member mRNA abundance measurements versus the 12 phenotypic parameters, of the 252 possible VMO category-phenotype measures there were 152 that had a positive *Z*_p_ value, and 100 that were negative (*P* = 0.001). For *Z*_p_ ≥ or ≤1.96 the numbers were 40 and 5, respectively (*P* <0.0001). At 3 and 12 months (Figure 3, B and C), the positive/negative *Z*_p_ counts were, respectively, 170/82 (*P* <0.0001) and 200/52 (P <0.0001), and the *Z*_p_ ≥ 1.96 and ≤ –1.96 were 58/6 (*P* <0.0001) and 72/1 (*P* <0.0001) (Supplemental Table 12, Supplemental Methods). The number of *Z*_p_ values ≥ 1.96 at all 3 time points is markedly greater than expected by random chance versus 2.5%, at months 0, 3, and 12, values were 40, 58, and 72 versus 3.8, 4.2, and 5.0 (*P* < 0.0001). In addition, the patterns of gene expression categories with *Z*_p_ ≥ 1.96 within phenotypic measures are different across the 3 time points (χ^2^ *P* = 0.003; Supplemental Table 12 and Figure 3) with the number of statistically significant *Z*_p_ generally increasing over time (proportional trend test *P* = 0.0006; Supplemental Table 13). However, for phenotypic measures, the pattern of statistically significant *Z*_p_ scores varies, which can be appreciated by inspection of Figure 4 and Supplemental Figure 9, A and B. Only 1 measure, HR, has a greater number of *Z*_p_ ≥ 1.96 at month 0 compared with months 3 or 12 — 9 versus 2 or 3, respectively (*P* = 0.019 by χ^2^, 0.026 by proportional trend; Supplemental Tables 13 and 14 and Supplemental Figure 9A). LVEF, RVEF, NE, PWP, and BMI (Figure 4 and Supplemental Figure 9, A and B) all exhibit increasing numbers of *Z*_p_ ≥ 1.96 with time, with proportional trend tests of *P* < 0.05. In contrast, ESV and EDV have more statistically significant *Z*_p_ values at 3 months versus either months 0 or 12 (Supplemental Tables 13 and 14, Figure 4, and Supplemental Figure 9, A and B).

The direct or inverse correlation of mRNA abundance within up- or downregulated genes on the LOCF analysis (Table 2) versus phenotypic measure is given in Figure 4 and Supplemental Figure 9, A and B, above the individual gene category bars, depicted as direct relationship (+), inverse relationship (−), or 0 (no statistically significant relationship). In all cases where a significant relationship was observed for both up-and downregulated genes (*n* = 48 of 170 possible combinations), respective inverse associations with phenotype measures were noted. For example, for PWP and metabolic genes, those that are upregulated on LOCF analysis have an inverse (−) relationship to PWP values, while downregulated genes have a direct (+) association, indicating that the expression of all genes in this category was associated with declining PWP values. For net association including both up- and downregulated genes, the color coding of the bar is inverse, meaning that upregulated genes correlated with declining PWP values were the dominant relationship.

**Figure 5:**
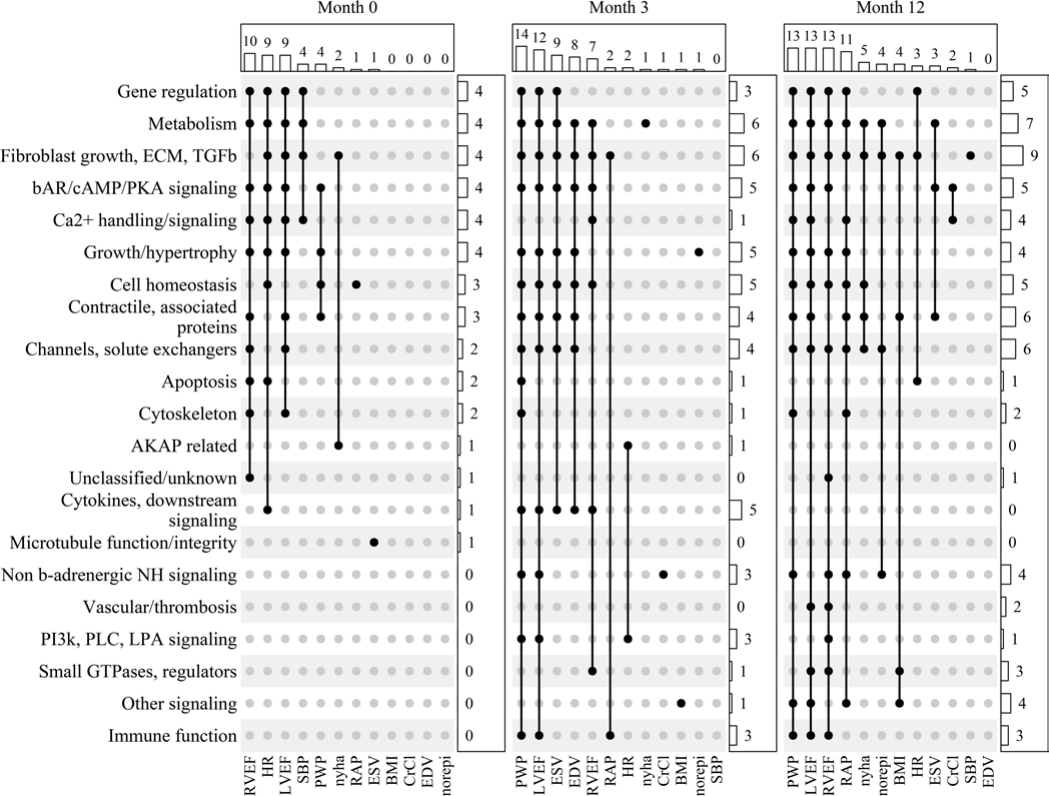
Upset plots of statistically significant gene expression biology categories vs. phenotypic measurements. Plotted are *Z*_p_ values ≥ 1.96 for β_1_-GSN ventricular myocardial ontology (VMO) categories versus non β_1_-GSN VMO controls, for 12 phenotypic measurement categories at months 0 (*n* = 46), 3 (*n* = 46), and 12 (*n* = 39) of beta-blocker treatment.

Upset plots comparing *Z*_p_ ≥ 1.96 at months 0, 3, and 12 are given in Figure 5. Here, the temporal course of the gene expression–phenotypic measure relationship is readily observed as is the aggregation of various gene category relationships to individual phenotypic measures. Note that the ordering by number of *Z*_p_ ≥ 1.96 from most to least is different at each time point and that 3 and 12 months are very different from month 0. Month 12 has greater numbers of aggregated significant correlations than month 3, but month 3 has statistically significantly larger *Z*_p_ values for ESV and EDV than month 0 or month 12.

#### Long noncoding RNAs.

**Figure 6:**
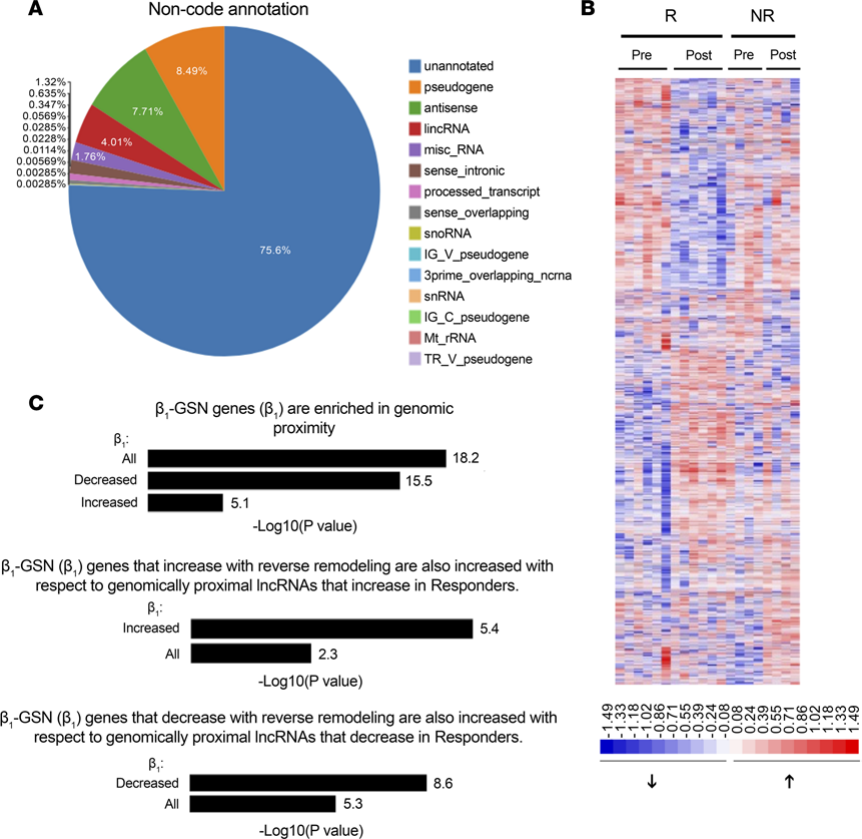
Lnc-RNAs in reverse remodeled DCMs. (**A**) Annotation of noncoding RNAs. (**B**) Heatmap of lncRNAs statistically significantly (fold change ≥ 1.5, *P* < 0.05 by Wilcoxon signed rank) changed in responders versus nonresponders in reverse remodeled human left ventricles from the superresponders (R_SR_, *n* = 6) and nonresponder (NR_SR_, *n* = 4) cohort. Pre, baseline value; post, last observation. Color intensity of log_2_ FPKM in the respective transcripts is normalized to the mean of all pre and post values. (**C**) Enrichment analysis of β_1_-GSN genes for proximity to changed lncRNAs. –Log *P* values at the end of bars are from a modified Fisher’s exact test to determine pathway enrichment, with a Benjamini-Hochberg correction for FDR.

To investigate the regulation of β_1_-GSN–responsive genes beyond the known input of TFs and downstream cross-regulation, we considered a role for lncRNAs since they are capable of contributing to gene network coordination (34). The RNA-Seq data were aligned to the NONCODE database, and the entire spectrum of identified non-coding RNAs are shown in Figure 6A. Unannotated species are by far the majority (75.6%), with pseudogenes (8.49%) and antisense (7.71%) also constituting larger fractions than annotated lncRNAs (4.01%). A total of 30,861 unique annotated or unannotated lncRNAs were identified in at least 1 subject by NONCODE (Supplemental Table 15).

Figure 6B is a heatmap of annotated and unannotated lncRNAs that changed from baseline to 12 months/LOCF. Using a fold change of 1.5 and a *P* < 0.05 for a meaningful change, 3,446 lncRNAs changed between baseline and end of study, 2,278 in the 6 analyzed superresponders (R_SR_) versus 1,168 in the 4 nonresponders (NR_SR_). In R_SR_ 1,118 lncRNAs were *P* < 0.05 for increased compared with baseline, and 1,160 were decreased. In NR_SR_, the number of increased lncRNAs was 690, compared with 478 decreased. None of the significantly changed lncRNAs that overlapped with a changed β_1_-GSN mRNA were annotated in GENCODE.

The *cis*-acting lncRNAs are positioned in close genomic proximity to the genes they regulate. For the changed lncRNAs in Figure 6B, we determined the closest coding gene and then asked if they were enriched in the β_1_-GSN versus the entire number of protein coding genes (PCG) in hg19 GENCODE (Figure 6C). In responders, 121 of the 430 (28%) member β_1_-GSN–responsive genes were the closest PCG to a changed lncRNA, compared with 315 of the total 19,500 PCGs (1.6%) (*P* = 6.3 × 10^–19^). Of the 121 β_1_-GSN–responsive genes that were the closest PGCs, 68 were associated with downregulated and 53 with upregulated transcripts, and each set of changed genes was enriched relative to respective decreases and increases in lncRNAs (Figure 6C). In contrast, lncRNAs significantly changed in nonresponders were not enriched in β_1_-GSN genes: all, *n* = 23, *P* = 0.48; upregulated, *n* = 12, *P* = 0.50; downregulated, *n* = 11, *P* = 0.88. A listing of the 121 nearest proximity β_1_-GSN members in responders is in Supplemental Table 16. For the 68 downregulated β_1_-GSN genes associated with changed lncRNAs in responders, 13, 6, 1, and 1 were the closest gene to 2, 3, 4, and 5 different changed lncRNAs, respectively, and the remaining 47 were the closest gene to a single lncRNA (Supplemental Table 16). For the 53 upregulated transcripts, 5 were from the closest genes to each of 2 and 3 lncRNAs and 43 genes were closest to a single lncRNA.

## Discussion

We have provided evidence of an extensive, 430-member gene network downstream from the β_1_-AR that participates in pathologic ventricular eccentric remodeling and its reversal.

### Global summary of gene expression changes

In addition to standard gene enrichment analyses, we utilized a VMO classification specifically designed for ventricular chamber remodeling of the human heart (9). Similarities between the general canonical pathway and VMO analyses included the metabolism categories such as upregulation in β-oxidation of fatty acids (in agreement with Wiki, Reactome; https://www.gsea-msigdb.org/gsea/msigdb/collection_details.jsp), fatty acid metabolism in general (Hallmark/KEGG), TCA cycle (Wiki/KEGG), branched chain amino acids metabolism (KEGG), and oxidative phosphorylation/electron transport (Hallmark/Wiki). Additional agreement occurred for downregulation in apoptosis (Hallmark) and fibrosis (Wiki). In addition, for biologic processes, there was approximate agreement with VMO for upregulation in force of contraction, branched chain amino acid metabolism and fatty acid β-oxidation, and downregulation in heart growth and collagen fibril organization.

By the VMO classification, the β_1_-GSN changes in gene expression were widespread across multiple biologic categories and consistent with the reversal of the structural and functional phenotypic characteristics of eccentric pathologic remodeling (2). Changes in mRNA expression occurred in key genes whose encoded proteins would positively influence contractility or chamber pump function (Ca^2+^ handling, β-adrenergic signaling, contractile proteins, apoptosis) and would lead to regression of pathologic hypertrophy (downregulation in growth promoting factors, fibrosis/ECM promoting and cytoskeletal genes, upregulation in microtubular function). Some of these changes have previously been identified (3, 9), but many have not been definitively connected to a β_1_-AR mechanism. Presumably most of the changes in gene expression in Tg mice and RR human LVs originated from β_1_-AR–expressing cardiac myocytes, but a myocyte origin is not a requirement for β_1_-GSN membership. For example, the marked number of fibrosis/ECM genes exhibiting downregulation in RR is likely mediated through fibroblasts, which do not contain β_1_-ARs, responding to the inhibition of microRNAs (35) and/or growth factor (36) secretion from cardiac myocytes.

Two large categories of changes likely underpinned the favorable changes in gene expression contributing to RR. Metabolism category genes were dramatically biased in favor of upregulation versus downregulation, with genes encoding mitochondrial proteins accounting for the majority of the imbalance. Improvement in depressed contractile function in the dilated, myopathic LV would require an enhanced and more efficient source of energy, which was likely supplied by upregulation in the expression of genes encoding fatty acid metabolism. Critical steps in fatty acid β-oxidation, respiratory chain function, branched chain amino acid metabolism, and metabolism with peroxisomal and membrane compartments were downregulated in β_1_-AR–overexpressing mice that ultimately develop a dilated cardiomyopathy (4, 5) and then upregulated on reverse LV remodeling in NDC patients treated with β_1_-AR antagonist treatment. The dramatic upregulation of genes involved in mitochondrial metabolism supports the concept that RR involves normalization of metabolic changes that have been previously described in end-stage HF, a shift from fatty acid oxidation and oxidative metabolism toward glycolysis and ketone utilization in the human heart (37). In pathologic ventricular remodeling neurohormonal signaling and mitochondrial function are generally linked (38), and the β_1_-GSN may be playing a major role in this connectivity.

The other large category of gene expression changes favoring upregulation versus downregulation was the gene regulation, transcription, and translation category. Extensive changes in gene expression would require multiple protein synthesis pathway changes, for which there was evidence at multiple levels, with 50 genes exhibiting upregulation versus 14 downregulated on RR. The upregulated genes included 15 that are involved in transcription regulation and 7 TFs, compared with only 3 and 2 in these respective categories.

Key to any proposed β_1_-GSN construct is the signaling architecture between the β_1_-AR receptor and nuclear gene regulation. β_1_-Adrenergic canonical and noncanonical signaling pathways that were likely involved through cross-regulation were altered in RR. With canonical β_1_-AR signaling, RR-associated mRNA changes were clearly biased in favor of restoration of the downregulated signaling present in Tg mice, with 13 changes in RR favoring increased cAMP/PKA signaling or improved neurotransmission versus 4 changes that would be predicted to decrease signaling. For potential GPCR cross-talk–mediated signaling, which — based on cognate gene expression changes — was extensive and involved multiple pathways and signaling effectors, phosphoinositide/phospholipase and non–β-adrenergic neurohormonal signaling exhibited numerically greater numbers of upregulated versus downregulated genes that would be expected to improve signaling. Moreover, the extensive involvement of small GTPases and their regulators could play a crucial role in transducing a single proximal signaling node into widespread changes in gene expression.

### Phenotypic association and temporal course

Serial gene expression measurements conducted at baseline, 3, and 12 months allowed for temporal assessment of β_1_-GSN gene category-phenotypic associations during RR. We used a variant of nonparametric permutation testing for these analyses, a valid method for generating a randomly distributed control (39). Compared with null/control mRNA abundance-phenotypic correlations empirically generated by 10,000 permutations using non-β_1_-GSN genes, β_1_-GSN member values were correlated with phenotypic measurements at all time points and well above (by 10- to 14-fold) the statistically expected number of *Z*_p_ ≥ 1.96 values based on the null. The numbers of statistically significant *Z*_p_ increased progressively from months 0 to 12, and for ESV and EDV, month 3 had higher *Z*_p_ scores than either month 0 or month 12. *Z*_p_ patterns also changed over time; at month 0, HR, LVEF, and RVEF had the most numerous *Z*_p_ ≥ 1.96 values, while at 3 months, it was ESV, EDV, LVEF, and RVEF, and at month 12, LVEF, PWP, RAP and RVEF had the most statistically significant *Z*_p_ values. Within categories, the directionality of relationships between gene expression and phenotypic measures was not qualitatively changed prior to RR (month 0) and after RR (months 3 and 12), indicating that gene expression and phenotype correlations that were statistically significant at month 0 and later at months 3 and 12 retained the same directional relationships.

In terms of which VMO categories may have been involved in initiation of the RR process, for increased intrinsic systolic function assessed by a decrease in ESV (40), at 3 months, there were 9 significant gene category correlations compared with 1 at month 0 and 3 at month 12. The 9 VMO categories with *Z*_p_ ≥ 1.96 at 3 months included several that would be expected to affect contractile function (metabolism, contractile and associated proteins, β-AR/PKA signaling, and channels and solute exchangers), with the gene regulation category providing the means for favorably altering contractility-enhancing gene expression.

For the other major component of remodeling, pathologic eccentric hypertrophy, a decrease in EDV as a measure of LV size also exhibited a burst of statistically significant *Z*_p_ values at month 3 (*n* = 8), with no *Z*_p_ ≥ 1.96 at either month 0 or 12. Gene categories that could have directly contributed to RR were the ECM/fibrosis and growth/hypertrophy categories. For EDV, the gene regulation category did not have a *Z*_p_ ≥ 1.96 at 3 months but was statistically significantly correlated with PWP and LVEF, the latter measure heavily influenced by EDV.

### Potential mechanisms for ensemble regulation or coordination of the β1-GSN

The myocardial remodeling β_1_-GSN is comprised of a relatively large number (430 is likely an underestimate) of genes whose products have major biologic importance for contractile function, tissue structure, and metabolism. These genes reside on multiple different chromosomes, and in order to be transcriptionally activated or repressed in a coordinated fashion — i.e., to function as a network — this diverse genomic mosaic would likely need a means of ensemble regulation or coordination. We have previously demonstrated that microRNAs are regulated in RR (41), and miR involvement in coordinating the β_1_-GSN is therefore likely. Based on their presence within the β_1_-GSN, coordinated TF regulation is almost certainly involved, as is extensive downstream cross-regulation that is a characteristic of GPCRs. Another candidate mechanism is superenhancer recruitment by BET domain protein family members such as BRD4 (32), which, through regulation of chromatin and other mechanisms, leads to myocardial pathologic gene expression (32, 42), including increased fibrosis (43). BRD4 has been shown previously to downregulate with LVEF recovery from ventricular assist device placement (42) and was downregulated on RR in the current study. Seven TFs were upregulated and 2 downregulated in RR, supporting the possibility of their superenhancer recruitment in the coordination of the β_1_-GSN in RR.

Changes in lncRNAs, as assessed by RNA-Seq and annotation of likely species, were more numerous in reverse remodeled responder LVs compared with nonresponders, by nearly a 2:1 ratio. None were annotated, preventing a computational analysis of their function. Unlike PCG, lncRNA lack evolutionary conservation (44), limiting the utility of common animal models to identify a focused subset of molecules, as done for PCG in this study. However, the proximal location of many lncRNAs to β_1_-GSN–responsive genes suggests that *cis*-regulation may be involved in the transcriptional response differentiating RR responders from nonresponders. Presumably, the role of *cis*-acting lncRNAs in gene network coordination would involve an interaction with a network-oriented TF or other factors acting as chromatin modifiers (45). Given the prior work investigating enhancer RNAs and superenhancers in adrenergic-stimulated pathologic cardiac models (42), this could account for a substantial portion of lncRNA expression observed in this study. However, the lack of H3K27ac and POL2 ChIP-Seq data currently prevent such an analysis. Considering the current inability to easily elucidate the function of lncRNAs computationally or by Tg models, future experimentation in human models is warranted and represents a potentially promising approach to identifying novel mechanisms involved in the ventricular remodeling process.

### Limitations

The gene expression readout was entirely composed of mRNA measurements, a consequence of the ability to measure mRNA abundance on a global expression level using small amounts of interventricular septum endomyocardial biopsies (EmBx) taken serially from the intact hearts of patients with HF (3, 9) and tissue sampling limitations for proteomic assessment. Although right ventricular accessed EmBx only extract tissue at a 1–2 mm depth of the mid-distal septum, we have shown that gene expression is indistinguishable from full septum expression in these samples, including at depths abutting the LV (46). Another limitation is that, although cardiac myocytes comprise approximately 70% of the volume of human ventricular myocardium, the cell of origin of the measured transcripts can only be inferred. In addition, although Tg mice had normal LVEF measurements (4, 5), undetected early LV remodeling rather than increased β_1_-AR signaling could have been responsible for some of the detected gene expression changes. Also, in view of the many additional gene expression changes associated with NDC RR (8, 9), the β_1_-GSN is likely one of many gene programs that can participate in pathologic ventricular remodeling and its potential reversal. Finally, despite equal doses of administered BBs and equivalent levels of β-blockade, nonresponders did not exhibit favorable changes in β_1_-GSN expression or in remodeling. The reason why inhibition of the β_1_-GSN was confined to 2 of 3 of the patients with DCM is being investigated in a current clinical trial testing the hypothesis that reversal of remodeling and adverse β_1_-GSN signaling by β_1_-AR blockade is dependent on a permissive effect of adequate heart rate reduction (47), as nonresponders in this study did not exhibit a reduction in heart rate.

### Conclusions

These data support the hypothesis that LV pathologic eccentric remodeling and its reversal by β-blocking agents are associated with gene expression changes within a gene network regulated by β_1_-AR signaling. These data indicate that the β_1_-AR system has important biologic consequences beyond the mediation of acute fight or flight physiologic responses — namely, when deployed chronically, activation of an extensive gene network that is involved in maladaptive myocardial remodeling.

## Methods

### Model system identification of genes whose myocardial expression is regulated by β1-AR signaling

#### Genes exhibiting mRNA expression changes in Tg mice overexpressing human ADRB1 389 polymorphic variants in the heart.

This methodology is described in Supplemental Methods.

#### Literature manual curation of experiments demonstrating changes in myocardial mRNA or protein expression by subacute or chronic administration of β-agonists or antagonists.

Evidence for β_1_-AR signaling was also sought by literature curation as described in Supplemental Methods. As for Tg mouse data, ≥ 2 statistically significant experiments or reports with same-directional change were required to qualify as β_1_-AR regulated. Curated evidence could also qualify with 1 statistically significant study, with a single *P* < 0.05 condition from the Tg mice that would not otherwise be eligible.

### BB-associated RR in nonischemic patients with dilated cardiomyopathy

RR responders and nonresponders were identified in the BB Effect on Remodeling and Gene Expression (BORG, NCT01798992) study as previously reported (9) and further described in Supplemental Methods. Global gene expression was measured as mRNA abundance in RNA extracted from right-sided mid-distal interventricular septum EmBx using microarrays in the 47 patient BORG EC, and RNA-Seq in a 12-patient super-responder cohort (SRC) (9) (Table 1). Prespecified candidate gene mRNA abundance (*n* = 50) was also measured in EC RNA by reverse transcription PCR (RT-PCR) (Supplemental Methods). Endomyocardial biopsies, right heart catheterization, and SPECT imaging were performed at baseline (time 0), 3 months, and 12 months.

### Detection of members of a β1-GSN operative in LV RR

We used a 3-tiered algorithm for selection of genes qualifying for membership in a β_1_-GSN consisting of (a) establishing that a gene alters its myocardial expression in response to changes in β_1_-AR input, (b) demonstrating these candidate genes change their expression during RR in response to β_1_-blockade in NDC patients, and (c) providing evidence of multigene network behavior (Supplemental Table 1, Supplemental Methods).

#### Gene ontology and enrichment analyses.

A modified Fisher’s exact test was used to determine pathway enrichment, with a Benjamini-Hochberg correction for FDR (48). Pathway databases were downloaded from the Broad Institute’s MSigDB (https://gsea-msigdb.org/gsea/msigdb). Myocardial biology gene ontology assignment of members of the β_1_-GSN to a previously described VMO classification tailored to eccentric pathologic remodeling (9) was performed.

### Specificity and temporal characteristics of the β1-GSN for effects on physiologic measures or clinical consequences of ventricular RR

In order to investigate the specificity of β_1_-GSN VMO categories to physiologic and clinical consequences of ventricular RR, the 21 VMO categories were compared by nonparametric permutation testing to 12 cardiac physiologic, pharmacodynamic, or clinical measures (phenotypic measures) for their relationships at baseline, 3 months, and 12 months. For these analyses, microarray identification of 19,673 transcripts in the 46 members of the entire cohort who had microarray measurements on biopsies taken at all time points were used to compare 430 β_1_-GSN member genes with 19,243 nonmembers.

### Identification of lncRNAs potentially involved in β1-GSN regulation

In RNA extracted from the 6 SRC R_SR_ and 4 age- and sex-matched NR_SR_, lncRNA was measured by RNA-Seq. Ribodepleted RNA-Seq reads were aligned via TopHat2 to NONCODE v4 and hg19. The resulting transcripts were then filtered by those that significantly changed in RR responders versus nonresponders (≥1.5 fold change, *P* < 0.05) using within-responder group analyses as described for mRNAs (Supplemental Methods).

lncRNAs were annotated from NONCODE using a minimum of a 99% overlap with the most recent GENCODE annotation from hg19. The *cis* regulation was inferred computationally using proximity between a given lncRNA and a corresponding mRNA transcript. Overlapping lncRNA and mRNA transcripts were identified using bedtools. Instances in which both transcripts significantly changed in expression were isolated as examples of potential *cis* regulation downstream of BB-mediated RR.

### Statistics

#### Statistical path to membership in the β_1_-GSN.

Tg mouse or curated source (Supplemental Methods) gene expression changes were matched to *P* < 0.05 opposite-direction changes for the same gene in RR human ventricular septal biopsy material. Using previously defined methodology (9), a gene’s transcript abundance was considered changed in the EC if it was *P* < 0.05 by RT-PCR or microarray in responders versus nonresponders (R_EC_ versus NR_EC_, or if the SRC if RNA-Seq data were *P* < 0.05 in R_SR_ (*n* = 6) versus NR_SR_ (*n* = 6). In addition, abundance change was considered statistically significant for a *P* < 0.05 in a paired analysis of LOCF versus baseline abundance in responders, provided that any same-direction change in nonresponders was *P* ≥ 0.10 (Supplemental Methods). The estimated net α and FDRs for Tg gene identification combined with human RR responder versus nonresponder statistical validation are given in the Supplemental Methods.

#### Statistical testing for β_1_-GSN membership.

In the Tg *ADRB1* 389Arg or 389Gly mice the original (4, 5), and the confirmatory statistical tests performed in the current study 1-way ANOVA with a Benjamini-Hochberg false discovery rate of 5% in TG1 mice (4) and 1-way ANOVA with a Bonferroni correction in TG2 mice (5). The multiple comparison testing assumed a 2-tailed distribution, and *P* < 0.05 was required for statistical significance for both ANOVA and multiple comparisons. For analysis of changes in human myocardial mRNAs, Wilcoxon rank-sum or Wilcoxon signed rank tests were used (3, 9). Categorical variables were compared using χ^2^ 1 × 2 configurations comparing the number of upregulated versus downregulated genes in a single category and 2 × 2 configurations comparing a single category or subcategory to all other categories in the data set. LVEF and LV volume changes were assessed by unpaired *t* tests. For temporal analysis of RR changes, 1-way ANOVA coupled with a Holm-Šídák test on paired values was used to assess changes from baseline at 3 and 12 months, followed by a linear trend test on unpaired values.

#### Temporal relationships of β_1_-GSN mRNA expression to phenotype measurements.

A variant of nonparametric permutation testing (49, 50) (summarized in Supplemental Figure 7 and Supplemental Methods) was employed to compare patterns of β_1_-GSN gene expression relative to cardiac and clinical phenotypic responses before and after RR. In separate analyses of data from months 0, 3, and 12, Spearman’s correlation coefficients (Rho values) were generated from individual gene mRNA abundance-phenotype measurements within each β_1_-GSN or null (control) permuted VMO category and averaged, from which a *Z*_p_ was calculated. The null effect was calculated using randomly selected gene sets obtained from the 19,243 microarray identified transcripts that were not members of the *n* = 430 β_1_-GSN. Further details of the permutation testing method are given in Supplemental Methods.

*P* < 0.05 was considered significant for all measurements, including for the 3-month-old TG1 mouse mRNA analysis (4) where a Benjamini-Hochberg test for FDR correction at α = 0.05 was applied (Supplemental Table 2 and Supplemental Methods). Analyses were performed using the R statistical suite version (v. 4.1) or GraphPad Prism. Heatmaps (9) and Upset plots (51) were generated as previously described.

### Study approval

The Tg mice studies were approved by the University of Maryland School of Medicine IACUC (4) (TG1) and the University of Colorado Health Sciences Center Animal Care Committee (5) (TG2). The methods of anesthesia and euthanization for TG1 mice were CO_2_ narcosis followed by cervical dislocation, and for TG2, a combination of xylazine and ketamine i.p. followed by cervical dislocation were used.

The clinical study (NCT01798992) was conducted according to Declaration of Helsinki guidelines, the protocol was approved by the University of Colorado and University of Utah IRBs, and all patients signed informed written consent.

### Data availability

Source data from the BORG trial are in the Supporting Data File. For the Tg mouse studies, source material are in Supplemental Table 3, with additional source data in Gene Expression Omnibus (GEO) (https://www.ncbi.nlm.nih.gov/geo/query/acc.cgi?acc=GSE11887) and in the GitHub repository (https://bristowlab.github.io/Tatman_7_2023/Tatman2023.html).

## Author contributions

PDT and DPK contributed conceptualization, methodology, formal analysis, writing of the original draft, and review/editing; KCC contributed formal analysis, writing of the original draft, and review/editing; IAC contributed methodology, formal analysis, conceptualization, investigation, validation, writing of the original draft, and review/editing; JAW contributed data curation, formal analysis, writing, and review/editing; CCS contributed investigation, formal analysis, resources, writing, and review/editing; JDP contributed validation, writing, and review/editing; BDL contributed investigation, writing, and review/editing; WAM contributed investigation, data curation, writing, and review/editing; SPH contributed formal analysis, validation, writing, and review/editing; AKF contributed formal analysis; EMR contributed data curation; SBL contributed investigation, methodology, resources, funding acquisition, writing, and review/editing; MRB contributed conceptualization, methodology, resources, funding acquistion, project administration, supervision, writing of the original draft, and review/editing.

## Supplementary Material

Supplemental data

Supplemental table 15

Supplemental table 16

Supplemental table 3

Supplemental table 6

Supporting data values

## Figures and Tables

**Table 2:**
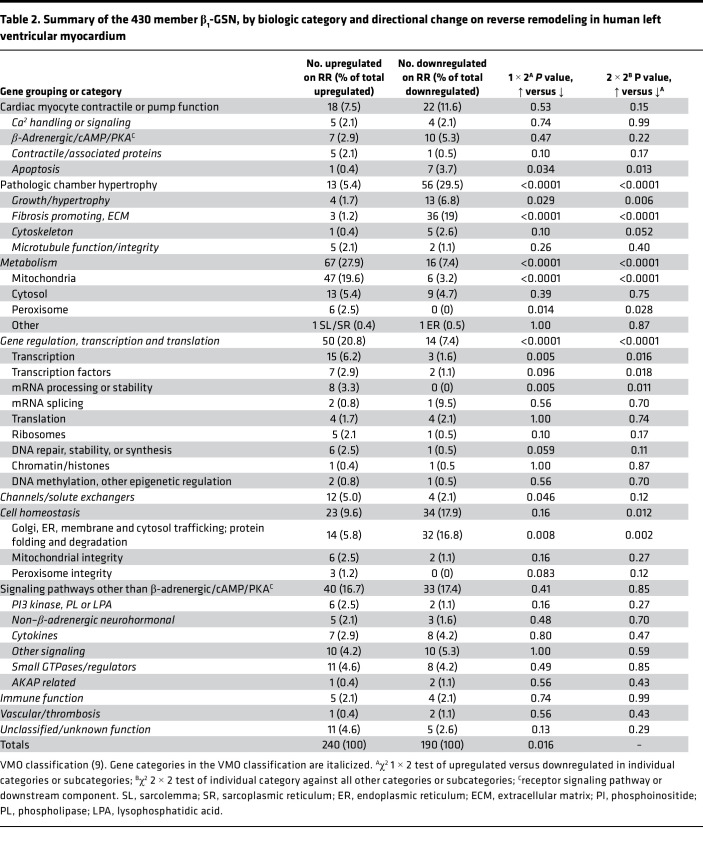
Summary of the 430 member β_1_-GSN, by biologic category and directional change on reverse remodeling in human left ventricular myocardium

**Table 1:**
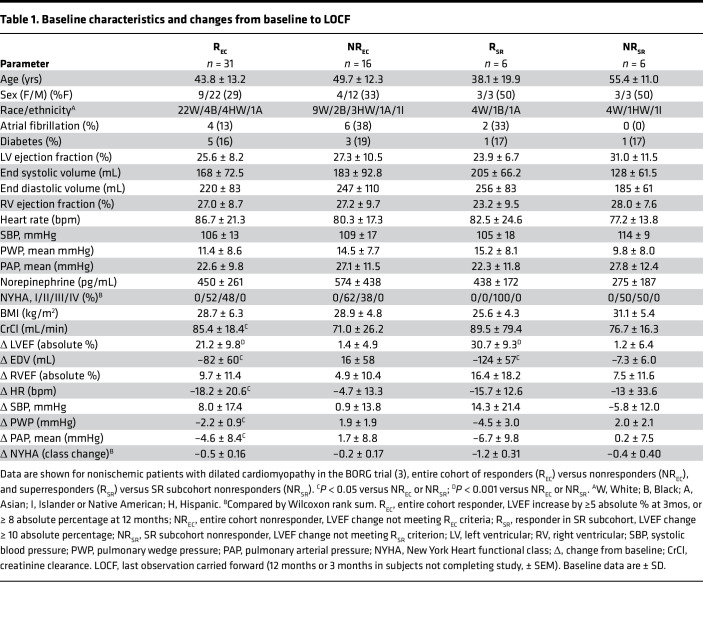
Baseline characteristics and changes from baseline to LOCF

**Table 3:**
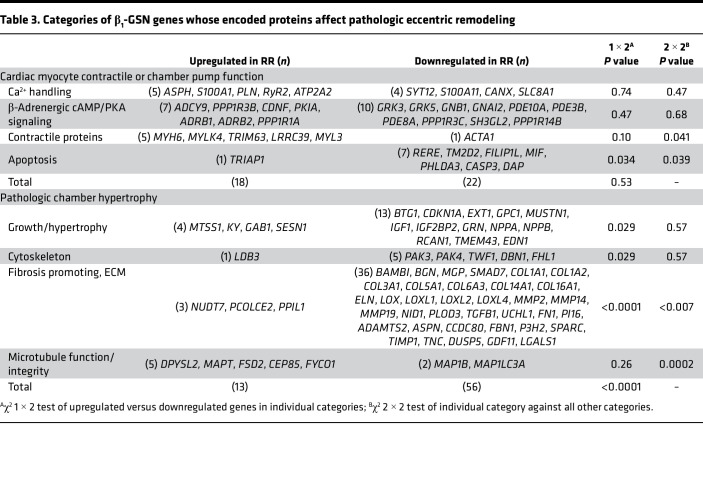
Categories of β_1_-GSN genes whose encoded proteins affect pathologic eccentric remodeling

## Version 1 08/22/2023

Electronic publication
