# Supplementary material for: An extensive β1-adrenergic receptor gene signaling network regulates molecular remodeling in dilated cardiomyopathies
Source: JCI Insight. 2023 Aug 22;8(16):e169720. doi: 10.1172/jci.insight.169720 (PMC10543724; doi:10.1172/jci.insight.169720)
Supplement: Supplemental data [file jciinsight-8-169720-s238.pdf]

SUPPLEMENTAL MATERIAL, TABLE OF CONTENTS

1.0 Identification of differences in mRNA Expression in *ADRB1* Arg389 or Gly389 Cardiac Overexpressor Transgenic Mice.

2.0 Literature Curation.

3.0 Serial Myocardial Gene Expression Measurements in Ventricular Septum of HFrEF Patients Undergoing Left Ventricular Reverse Remodeling.

4.0 Statistically Significant Changes in mRNA Expression in Reverse Remodeling Responders.

5.0 Nonparametric Permutation Testing.

6.0 β_1-GSN Membership assigned to: Cell Homeostasis; Signaling Pathways Other than β-adrenergic/cAMP/PKA; Channels and Solute Exchangers; and Immune Function, Vascular/Thrombosis, and Unclassified Gene Categories.

7.0 Correlation of Metabolism gene**s** normalized mean mRNA abundance vs. LVEF or PWP in 46 study subjects at Months 0, 3 and 12.

8.0 Supplemental Material References.

9.0 Supplemental Tables:

Table S1. Three-tiered qualification for membership in the β_1-adrenergic receptor gene signaling network (β_1-GSN): algorithm for demonstrating pharmacologic concordance.

Table S2. Flow of Tg mouse and literature curated candidate genes through confirmation by antithetical changes in reverse remodeled human ventricular myocardium into final N=430 β_1-Gene Signaling Network membership.

Table S3. Genes with mRNA abundance changes in transgenic mice: **A**, 3 months old (TG_1); **B**, 3 or 6-8 months (TG_2).

Table S4. Literature curated genes whose mRNA or protein expression was quantitatively changed in myocardium or cardiac myocytes by β-agonist or -antagonist exposure of ≥4 hours; agonist directional change opposite and antagonist change in the same direction as in RR Responders vs. Nonresponders in BORG.

Table S5. Numbers of genes upregulated or downregulated in Responders vs. Nonresponders.

Table S6. Gene List of Changes (N=430) from biologic filters by Biologic Category: **A** (Sheet 1), Downregulated in Tg, upregulated in reverse remodeling; **B** (Sheet 2), Upregulated in Tg, downregulated in reverse remodeling.

Table S7. Subcategories of genes whose encoded proteins affect metabolism.

Table S8. Subcategories of genes whose encoded proteins affect gene regulation.

Table S9. Upregulated vs. downregulated genes by subcategory for **A.** Channels/Solute Exchangers., **B.** Cell Homeostasis;

Table S10. A. Non-β-adrenergic canonical signaling pathway genes with changed expression by category, and **B.** Immune function, vascular/thrombosis and unclassified categories.

Table S11. Baseline (0 months), 3 months and 12 months data for the BORG study entire cohort used in Nonparametric Permutation Testing (N=46), Responders and Nonresponders.

Table S12. Spearman's rho values and curve slopes for rank correlation between gene categories and phenotypic measurements for β_1-GSN mRNA abundance vs. physiologic or clinical measurement.

Table S13. Z_p ≥1.96 by VMO categories and phenotypic measures.

Table S14. Z_p value summary and directional correlation of VMO category vs. phenotype measurement.

Table S15. All lncRNAs identified by NONCODE.

Table S16. 121 β_1-GSN genes that are the closest proximity to lncRNAs change with reverse remodeling in Responders: **A**, Downregulated β_1-GSN that are the closest genes to changed lncRNAs; **B**, Upregulated β_1-GSN closest genes to changed lncRNAs.

10.0 Supplemental Figures:

Figure S1. Canonical pathway enrichment of downregulated genes in the β_1-gene signaling network.

Figure S2. GO pathway enrichment of downregulated genes in the β_1-gene signaling network.

Figure S3. Canonical pathway enrichment of upregulated genes in the β_1-gene signaling network.

Figure S4. GO pathway enrichment of downregulated genes in the β_1-gene signaling network.

Figure S5. Reactome enrichment map of the β_1 gene signaling network.

Figure S6. Cytoscape network modeling of β_1-GSN members within VMO categories involved in **A.** eccentric pathologic remodeling and its reversal, and **B**. Metabolism.

Figure S7. Scheme for Nonparametric Permutation Testing and generation of Z_p values for β_1-AR Gene Signaling Network (β_1-GSN) vs. non β_1-GSN Controls, and determination of directionality of β_1-GSN) Ventricular Myocardial Ontology (VMO) categories vs. Phenotype measurements.

Figure S8. Plots of mRNA abundance vs. LVEF or PWP in genes that were A. Upregulated, B. Downregulated in reverse remodeling Responders.

Figure S9. Temporal pattern of mRNA abundance-ventricular function and structure phenotypic relationships, **A.** ventricular filling pressures and pharmacodynamics, **B.** clinical parameters.

11.0 Funding Sources

SUPPLEMENTAL MATERIALS

1.0 Identification of differences in mRNA Expression in *ADRB1* Arg389 or Gly389 Cardiac Overexpressor Transgenic Mice

In Tg mice cardiac overexpressing the Arg389 or Gly389 polymorphic variant of the human *ADRB1*, a dilated cardiomyopathy (DCM) characterized by a reduced LVEF develops in Arg389 but not Gly389 mice at 6-8 months (1,2). LVEFs are in the normal range in Gly389 mice at 6-8 months as well as at 3 months in both Arg389 and Gly389 mice (1,2). To identify genes regulated by β_1-AR signaling, we analyzed Tg mice data from two of our previously reported *ADRB1* overexpression studies, conducted in lineages from the same founding stock (1,2).

ADRB1 Arg389 or Gly389 receptor variants were overexpressed in the heart by 40 (1) or 25 (2) fold, using the cardiac specific human *MYH6* promoter. RNA extraction from LV myocardial tissue was performed as previously described (1,2). One dataset (TG1) was from 3 months old (1) and the other (TG2) was from either 3 or 6-8 months old (3/6-8 months) mice (2). In both datasets global mRNA expression was measured by microarrays (1,2) and compared to nontransgenic (NTG) littermate controls. In order for a gene to be considered as regulated by β_1-AR signaling mRNA abundance had to be same-directional changed in both Arg389 and Gly389 TG1 overexpressor mice, and in at least 2 of the 3 timepoints at 3 months or Gly389 mice at 6-8 months in the TG2 mice **(Supplemental Material, Section 1.0, Table S1)**.

The 3 months old-only TG1 non-transgenic (NTg) and Tg overexpression of *ADRB1* 389Arg or 389Gly and *ADCY5* experiments consisted of 6 mouse hearts/group (1). We confirmed and used the original statistical analysis that evaluated microarray (Affymetrix GeneChip^{TM} Mouse Genome MOE 430 plus 2.0 array) measured mRNA abundance measurements in the 4 groups (Nontransgenic (NTg) controls, *ADCY5*, *ARDB1* Arg389 or *ADRB1* Gly389 overexpressors) by ANOVA followed by Benjamini-Hochberg test of each Tg group vs. NTg control with a false discovery rate of 5%. If an mRNA change was $P < 0.05$ in both Arg389 and Gly389 animals vs. control the gene was considered to have been potentially regulated by β_1-adrenergic receptor signaling. The estimated alpha based on these steps is 0.0005, and the false discovery rate (FDR) is 11 of 21,814 unique GenBank RefSeq transcripts on the array (**Table S2**). When the criterion of $P < 0.05$ opposite directional changes on RR by β_1-AR blocking agents in nonischemic dilated cardiomyopathies is added, the alpha is 0.000013*(0.0005*0.05/2) and the false discovery rate (FDR) is 0.28 transcripts.

In the 3 and 6-8 months old Tg mouse dataset (2) microarray (Affymetrix GeneChip^{TM} Mouse Genome 1.0 ST Array, 21,814 unique GenBank RefSeq transcripts (**Table S2**) measurements of mRNA abundance (TG2) changes were analyzed at the 3 months (NTg, Gly3, Arg3) and 6-8 months (NTg, Gly6, Arg6) timepoints as in the original analysis (2), against the respective NTg controls. Because a DCM develops in Arg6 mice the measured gene expression changes in these animals may reflect both the effects of ventricular remodeling and β_1-AR signaling, only changes in Arg3, Gly3 and Gly6 mice were considered eligible. At these timepoints and receptor variants, genes whose mRNA abundance changes vs. NTg controls were P <0.05 (two sided) and whose fold change was ≥1.25 fold (2) were considered statistically significant. Confirmed evidence for regulation by β_1-AR signaling was taken as P <0.05 changes vs. NTg controls in 2 of the 3 eligible timepoint/variants, in the same direction. The estimated alpha for these conditions is 0.0036, with an FDR of 84 of the 24,009 unique transcripts on the array. When the criterion of opposite directional changes on RR is added, the alpha and FDR are respectively 0.00009 (0.0036*0.05/2) and 2.2 transcripts.

2.0 Literature Curation

Literature manual curation (**Table S4**) was conducted independent of the transgenic mouse identification of candidate genes, and used combinations of search terms such as "isoproterenol, beta receptor agonists, beta receptor antagonists" coupled with cardiac or heart "gene expression, mRNA expression, protein expression, or gene regulation". NCBI PubMed and Google Scholar were the main sources searched. Acceptance of a curated gene as a β_1-GSN member was determined by the same adjudication system described for assignment into the ventricular remodeling ontology classification. Firm evidence included at least 2 studies or 2 separate experiments within a single study with P <0.05 gene expression changes in the same direction, or

a single study or experiment bolstered by data from *ADRB1* overexpressor Tg mouse experiments. The supportive Tg data had to be a $P < 0.05$ in one of the variants and conditions used in the Tg biologic filter identification, with a 2nd variant condition changed in the same direction at a $P < 0.10$. The alpha calculation based on 2 same direction experiments at $P < 0.05$ and then in RR an antithetical change of $P < 0.05$ for agonists or same direction for antagonists is ((0.05^3)/4) or 0.00003. Based on an estimate of 19,370 protein coding genes in the human genome (3) the FDR is 0.6 transcripts.

3.0 Serial Myocardial Gene Expression Measurements in Ventricular Septum of HFrEF Patients Undergoing Left Ventricular Reverse Remodeling

In the Beta-blocker Effects on Remodeling and Gene Expression (BORG) study (NCT0178992) (4, 5), β-blocker naïve nonischemic, nonvalvular/idiopathic dilated cardiomyopathy (NDC) patients, entire cohort (EC, N=47, LVEF 24±9%) were randomized to either metoprolol succinate, metoprolol succinate + doxazosin, or carvedilol, all arms of which contain a β-antagonist that blocks β_1-ARs (4). SPECT imaging radionuclide ventriculography and right ventricular mid-distal septum endomyocardial biopsies were performed at baseline, 3, and 12 months. Eight of the 47 EC subjects had only 3 month LVEF, LV volume and gene expression measurements (5) and were last observation carried forward (LOCF) for inclusion with the 39 subjects who had both 3 and 12 month follow-up studies. Presence of LV reverse-remodeling (RR) was defined as an increase in EF of ≥8 absolute % at 12 months or ≥5 % by 3 months (Responders, R_{EC}), and Nonresponders (NR_{EC}) were subjects with LVEF changes not meeting these criteria. In the Super-responder (SR) cohort responders (R_{SR}) were defined as those having an LVEF improvement ≥10 absolute % at either 3 or 12 months, and were paired

with 6 age- and sex-matched NRs to form a R_{SR} subcohort in which Nonresponders (NR_{SR}) were patients with an LVEF change <5 % (5).

The EC was relatively young (46±13 years), with NYHA Class II and III heart failure and moderately severe LF dysfunction and remodeling (mean LVEF 26±9%). All subjects were diagnosed with idiopathic dilated cardiomyopathy (IDC), and none had a familial history or genetic testing revealing a likely cause of cardiomyopathy. All had angiographically confirmed unobstructed coronary arteries. Exclusion criteria included HF due to valvular disease, thyroid disease, obstructive or hypertrophic cardiomyopathy, pericardial disease, amyloidosis, or myocarditis. Patients considered heart transplant candidates were excluded, and patients could not be receiving β-blockers or β-agonists or have decompensated HF at the time of randomization or baseline studies (4). No patient had a family history of NDC or sudden cardiac death.

LVEF, LV volume and hemodynamic changes in Responders and Nonresponders during the 3 or 12 months follow-up (4,5) for the EC and in the 12 member SR subcohort assessed at LOCF are given in **Table 1**. In both cohorts LVs undergo substantial RR, with an R_{EC} LVEF change at LOCF by 21 absolute % (P <0.001 vs. NR_{EC}s) and by 31 absolute % in the 6 R_{SR}s, P <0.001 vs. the 6 age/sex matched NR_{SR}s (**Table 1**). Consistent with the LVEF changes, LV diastolic volume (EDV) measured by SPECT imaging was also decreased in both R_{EC}s and R_{SR}s (**Table 1**), and on paired analysis in R_{EC}s both EDV and LV end systolic volume were decreased at 3 and 12 months (**Table S11**). When compared to NR_{EC}s RVEF was not changed by an unpaired t-test (**Table 1**), but on paired analyses was increased in R_{EC}s at both 3 and 12 months (**Table S11**). Note that in **Tables 1** and **S11**, in addition to the lack of eccentric remodeling improvements NR_{EC}s do not have a statistically significant reduction in resting heart rate.

RNA from endomyocardial biopsies was extracted as previously described (4,5). Global changes in myocardial mRNA expression in the NDC patients' biopsies were quantified by cDNA hybridization to the Affymetrix HGU-133 Plus 2.0 Gene Chip and compared between R (n=31) and NR (n=16), and in the Super-responder cohort (6 SR and 6 NR_{SR}) using RNA sequencing (RNA-seq) (5). As previously described (5), the number of subjects available was the entire 47 for RT-PCR (4, 5), 46 for microarray (5) (one subject had a missing baseline value) and 12 (6 SRs and 6 matched NR_{SR} controls) for RNA-seq (5). The mRNA expression of 50 candidate genes was also measured by RT-PCR (4,5), in RNA from all 47 NDC patients' biopsies.

4.0 Statistically Significant Changes in mRNA Expression in Reverse Remodeling Responders

To qualify as an R vs. NR change, genes had to have a significantly different ($P < 0.05$) expression change between grouped R vs. NR data, or exhibit significantly increased expression compared to baseline in R but not NR (same direction changes of $P \geq 0.1$ or opposite directional changes) as measured by microarray or RT-PCR in the EC, or RNA-Seq in the SR cohort. For reference to Tg mouse or literature curated β-agonist treatment data the BORG R vs. NR change had to be directionally opposite, whereas curated β-antagonist changes had to be in the same direction as the RR changes.

5.0 Nonparametric Permutation Testing

The nonparametric permutation testing approach to generating correlations between microarray mRNA abundance and phenotypic measures at baseline (N=46) and after 3 (N=46) and 12 months (N=39) of β-blocker treatment is represented in **Figure S7**. Sampling permutation was used to create 10,000 random genesets, which were partitioned to match the sizes of equal

size of the β_1-GSN comparator VMO categories. Z_p was calculated using the formula: $Z_p = (\overline{nR_\beta} - \overline{pR_C})/S_{pRC}$ where $\overline{nR_\beta}$ is the mean of the VMO category β_1-GSN absolute Rho values, $\overline{pR_C}$ is the mean of the permuted VMO category absolute control/null Rho values, and S_{pRC} is the standard deviation of the permuted Rho values, with normality assumed for the 10,000 samples comprising the empirical sampling distribution of the mean control/null effect.

Based on the relationship of the averaged β_1-GSN Rho values to the null within each gene category, positive Z_p values represent a greater than expected correlation between VMO category mRNA abundance and phenotypic measure, with negative values indicating a weaker than expected correlation (i.e. if the null absolute Rho value is larger than the β_1-GSN mean Rho). Statistical significance was set at a two-tailed $\alpha = 0.05$, corresponding to $|Z_p| \geq 1.96$ and rounded to ≥ 2.0 in heat maps and in the text in **Figure S7**.

For gene category-phenotype Z_p values ≥ 1.96, average Rho positive or negative values of individual gene mRNA abundance-phenotype measures were generated in each of the identified upregulated and downregulated gene sets (**Table 2**) to determine if the correlation with each phenotypic measure was direct or inverse. To derive the sum/net correlation directionality of the VMO category with the phenotypic measure a Wilcoxon rank sum test was used to determine if the net Rho was $P < 0.05$ compared to a zero correlation.

6.0 Changes in Cell Homeostasis, Signaling Pathways Other than β-adrenergic/cAMP/PKA, Immune Function, Vascular/thrombosis, and Unclassified Ventricular Myocardial Ontology Categories

6.1 Cell Homeostasis

6.1.1 Golgi, ER, Sarcolemmal and Cytosol Trafficking; Protein folding and Degradation

A large number (N=57) of Tg genes with changed expression on RR were assigned to the Cell Homeostasis category, 23 upregulated vs. 34 downregulated genes (P=0.16, **Table 2).** Of the downregulated genes, 32 are in the Golgi, ER, Membrane and Cytosol Trafficking, or Protein Folding/degradation subcategory, compared to 14 upregulated (P=0.008, **Table S9B**).

6.1.2 Mitochondrial Integrity

For the Mitochondrial Integrity subcategory, the trends are reversed, with 6 upregulated and 2 downregulated (P=0.16 1x2 Chi square, P=0.031 2x2 chi square, **Table S9B**). One of the upregulated genes, *OMA1*, encodes a mitochondrial membrane protease that is a primary determinant of fusion competence and mitochondrial integrity (6).

6.1.3 Peroxisome Integrity

All 3 Peroxisome Integrity genes with changed expression were upregulated (P= 0.083, P=0.031 for 2x2 Chi square, **Table S9B)**.

6.2 Signaling Pathways Other than β-adrenergic/cAMP/PKA

We included in the classification scheme signaling pathways that may cross-regulate with β-adrenergic signaling (**Tables 2, S6, S10A**), and the data indicate that β_1-GSN signaling extends extensively beyond the canonical cAMP-PKA pathway.

6.2.1 Phosphoinositide, Phospholipase or Lysophosphatidic Acid

There were 6 upregulated genes vs. 2 downregulated in this category (P= 0.16, **Tables 2, S6, S10A**). Of the upregulated genes, 2 each are in the PLC (*PLCD3, PLCL2* (inactive)) and PLD (*PPAP2B, PPAPDC3*) pathways, one interacts with IP3 (*ARAP2*) and one (*PIK3IP1*) is a negative regulator of phosphatidylinositol 3-kinase (PI3K). One of the two downregulated genes (*SH3D19*) encodes an activator of EGFR and IP3 kinase signaling, Eve-1, providing evidence of decreased activity of this pathway that would result in an anti-hypertrophic effect. The other

downregulated gene, *PLCG2*, also encodes a pro-hypertrophic signaling molecule, based on the properties of its highly homologous gamma-1 isozyme (7).

6.2.2 Non-β-adrenergic Neurohormonal

This category features 5 upregulated genes: angiotensin II type 1 receptor (*AGTR1*); alpha 1A adrenergic receptor (*ADRA1A*); purinergic receptor P2Y1 *(P2RY1*); a nuclear receptor/transcription factor (*NR3C2*) that binds to mineralocorticoid response elements; and *ART3*, an ADP-ribosyltranferase listed as inactive in GenBank (**Tables 2, S6, S10A**). The 3 downregulated genes (P=0.48) in this category are the G protein γ subunit *GNG12* whose activity is regulated by PKCα, a gene for a G-protein coupled chemerin-like receptor (*CMKLR1*) that may induce insulin resistance in response to the adipokine chemerin (8), and *PDE4B*, the encoded protein effect of which would be expected to increase cAMP levels.

6.2.3 Small GTPases/Regulators

This category, whose members are coupled to multiple signaling pathways including β_1-adrenergic, PI3 kinase Phospholipase C and others, has 18 changed genes (11 upregulated, 8 downregulated (P=0.49), **Tables 2, S6, S10A**), the largest number in designated signaling pathways or components. Of the 11 upregulated genes, 5 are small GTPases (3 Ras family (*NKIRAS1*, *RAB12*, *RASD2*), 1 Rho (*RHOT1*), 1 Rab (*RAB12)*), 3 are guanine nucleotide exchange factors (GEFs), 2 are small GTPase activating proteins (*ARHGAP9, RICS*), and 1 (*GPSM1*) is a G-protein signaling modulator (RGS-like protein). Of the 8 downregulated genes in this category 2 are GEFs (*DOCK1, RABGEF1*), and 1 each is a small GTPase (*RAB23*), a Ras associated factor (*RASSF2*), a Rab acceptor/receptor (*PRAF2*), a small GTPase binding protein/inhibitor (*EHBP1L1*) and an ADP ribosylation factor (*ARF4*).

6.2.4 Cytokines

This category was nearly evenly divided between upregulated (N=7) and downregulated (N=8) genes (**Tables 2, S6, S10A**). The downregulated genes include interleukin receptor 1 (*ILR1*), interleukin 6 (*IL6*), and inducible nitric oxide synthase 2 (*NOS2*). Two genes encoding TNFα (*TNFAIP6*) or TNF receptor associated proteins (*TRAF4*) were also downregulated, as were genes for a novel adipokine (*CIQTNF6*), a small chemokine (*CXCL16*) and a SOCS family protein (*SOCS2*). The 7 upregulated genes include 4 (*ASB4*, *ASB10*, *ASB14*, *ASB15*) encoding for ankyrin repeat and SOCS box containing proteins that are negative regulators of cytokine signaling. The gene encoding interleukin 15 (*IL15*), a mitochondrial TNF associated protein (*TRAP1*) and a negative regulator of IKKB (*KLHL21*) were also upregulated.

6.2.5 Other Signaling Pathways

There were 10 upregulated and 10 downregulated genes not classified in signaling pathways other than PI3 kinase/PL/LPA, Non-β-adrenergic Neurohormonal. and Cytokines. (**Tables 2, S6, S10A**). The cardioprotective epidermal growth factor gene (*EGF*) gene was upregulated with RR, as was a gene for a protein kinase C isoform (*PRKCQ*) that protects against pathologic remodeling (9), and an integrin (*ITGB6*). Among the downregulated genes were those encoding protein kinase Cα (*PRKCA*), decreases in which increase contractility (10), a sphingosine-1 phosphate receptor (*S1PR1*), and a protein phosphatase (*PPM1E*). Another downregulated gene was PAK1 (p21 (RAC1) which has previously been shown to be under β_1-AR control and whose encoded protein is responsible for microtubule densification in pathologic hypertrophy (11).

6.2.6 AKAP Related

Of the 2 downregulated AKAP genes, 1 (*AKAP13*), encodes a protein that couples α_{1A}- and α_{1B}-ARs to MAP kinase, and the other (*AKAP2*) is associated with the actin cytoskeleton where it binds to a regulatory subunit of PKA (**Tables 2, S6, S10A**). The upregulated AKAP (*AKAP8*),

also known as AKAP95, is a nuclear based AKAP involved in cell cycle regulation and chromatin condensation.

6.3 Immune Function, Vascular/Thrombosis and Unclassified Gene Categories

These categories were comprised of relatively few genes, none of which were differentially up-or downregulated (**Tables 2, S6, S10B).**

7.0 Representative Plot of VMO Category (Metabolism) mRNA Abundance vs. Phenotypic Measurements (LVEF, PWP)

A variant of nonparametric permutation testing was used to assess relationships between microarray mRNA abundance in the 430 β_1-GSN genes, referenced against the 19,243 non-β_1-GSN genes that had transcripts identified. These analyses were performed on an entire cohort of 46 subjects, since 1 patient had a missing microarray measurement at Month 0. **Figure S7** gives an overview of this methodology, and **Figure S8** gives examples of the correlation plots of mean mRNA abundance vs. phenotypic measurements of LVEF and PWP mean pressure using the 67 Metabolism genes that were upregulated and 16 that were downregulated in LOCF R_{EC}s. Months 0, 3 and 12. The statistical analysis is by Spearman's rank correlation generating Rho and P values. For the 67 upregulated genes (**Figure S8A**), at Month 0 vs. LVEF measurements there is no statistically significant relationship, but at months 3 and 12 the correlation is direct, with increasing mRNA abundance associated with higher LVEF values. For PWP the relationships are, as expected, inverse at 3 and 12 months, with increasing mRNA abundance associated with decreasing PWP. At Month 0, i.e. prior to β-blocker treatment and reverse remodeling there is no statistically significant relationship.

For the 16 downregulated genes (**Figure S8B**), compared to upregulated genes and as expected for both LVEF and PWP the directionality is reversed, with decreasing mRNA

abundance associated with increasing LVEF and decreasing PWP at both 3 and 12 months. However, unlike for upregulated genes the relationships are statistically significant at Month 0 with the same patterns as in Months 3 and 12. This raises the possibility that the genes that downregulated with reverse remodeling, which had higher baseline expression, may have been exerting negative effects on the failing heart at Month 0 since increasing mRNA abundance was associated with decreasing LVEF and increasing PWP.

8.0 References for Supplemental Material

1. Swift SM, Gaume BR, Small KM, Aronow BJ, Liggett SB. Differential coupling of Arg- and Gly389 polymorphic forms of the beta1-adrenergic receptor leads to pathogenic cardiac gene regulatory programs. *Physiol Genomics* 2008;**35**:123-131.
2. Dockstader K, Nunley K, Karimpour-Fard A, Medway A, Nelson P, Port JD, Liggett SB, Bristow MR, Sucharov CC. Temporal analysis of mRNA and miRNA expression in transgenic mice overexpressing Arg- and Gly389 polymorphic variants of the β1-adrenergic receptor. *Physiol Genomics* 2011;**43**:1294-1306.
3. Liu C, Bai B, Skogerbø G, Cai L, Deng W, Zhang Y, Bu D, Zhao Y, Chen R. NONCODE: an integrated knowledge database of non-coding RNAs. *Nucleic Acids Res*. 2005;**33**(Database issue):D112-115. doi: 10.1093/nar/gki041. https://pubmed.ncbi.nlm.nih.gov/15608158/ (accessed 9/1/22).
4. Kao D, Lowes B, Gilbert E, Minobe W, Epperson LE, Meyer L, Ferguson D, Volkman K, Zolty R, Borg D, Quaife R, Bristow M. Therapeutic Molecular Phenotype of β-blocker Associated Reverse-Remodeling in Nonischemic Dilated Cardiomyopathy. *Circ Cardiovasc Genet* 2015;**8**:270-283.
5. Toni LS, Carroll IA, Jones KL, Schwisow JA, Minobe WA, Rodriguez EM, Altman NL, Lowes BD, Gilbert EM, Buttrick PM, Kao DP, Bristow MR. Sequential analysis of myocardial gene expression with phenotypic change: Use of cross-platform concordance to strengthen biologic relevance. *PLoS ONE* 2019;**14**:e0221519.
6. Baker MJ, Lampe PA, Stojanovski D, Korwitz A, Anand R, Tatsuta T, Langer T. Stress-induced OMA1 activation and autocatalytic turnover regulate OPA1-dependent mitochondrial dynamics. *EMBO J* 2014;**33**:578-593.
7. Dent MR, Dhalla NS, Tappia PS. Phospholipase C gene expression, protein content, and activities in cardiac hypertrophy and heart failure due to volume overload. *Am J Physiol Heart Circ Physiol* 2004;**287**:H719-727.
8. Roman AA, Parlee SD, Sinal CJ. Chemerin: a potential endocrine link between obesity and type 2 diabetes. *Endocrine* 2012;**42**: 243-251.
9. Paoletti R, Maffei A, Madaro L, Notte A, Stanganello E, Cifelli G, Carullo P, Molinaro M, Lembo G, Bouché M. Protein kinase Cθ is required for cardiomyocyte survival and cardiac remodeling. *Cell Death Dis* 2010;**1**:e4.
10. Dorn GW 2nd, Force T. Protein kinase cascades in the regulation of cardiac hypertrophy. *J*

Clin Invest 2005;**115**:527-357.
11. Cheng G, Kasiganesan H, Baicu CF, Wallenborn JG, Kuppuswamy D, Cooper 4th G. Cytoskeletal role in protection of the failing heart by β-adrenergic blockade.*Am J Physiol Heart Circ Physiol* 2012;**302**:H675-H687.
12. Shannon P, Markiel A, Ozier O, Baliga NS, Wang JT, Ramage D, Amin N, Schwikowski B, Ideker T. Cytoscape: a software environment for integrated models of biomolecular interaction networks. *Genome Research* 2003;**13**:2498-2504.

9.0 Supplemental Tables

Table S1. Three-tiered qualification for membership in the β_1-adrenergic receptor gene signaling network (β_1-GSN): algorithm for demonstrating pharmacologic concordance between biofilter or curated evidence and human myocardial RR changes in gene expression.

**Tier I: Biologic filter from Transgenic (Tg) Mice or Literature Curation**	**A. *ADRB1* Arg389 or Gly389 cardiac overexpressor Tg mice**<br>1. TG1 (1), 3 months old mice with either Arg389 or Gly389 overexpression, all with normal LVEFs.<br>2. TG2 (2), 3 months old mice with either Arg389 or Gly389, 6-8months old mice with Gly389, all with normal LVEFs.<br>3. Algorithm for a change in gene expression, statistically significant same direction changes (**Section 1.0**) in<br>a. TG1, Gly3 + Arg3 OR<br>b. TG2, in at least 2 of Gly3, Arg3 or Gly6-8<br><br>OR<br><br>**B. Literature curation**<br>1. Genes that change expression in myocardium or in cultured cardiac myocytes in response to a β-agonist or -antagonist administered for ≥4 hours; need at least 2 separate reports or replicate sets in same report.<br><br>AND
**Tier II: Confirmation in human heart**	**A. Responder (R) vs. Non-responder (NR) changes on RR in human ventricular myocardium, BORG Study candidate or global genes, Entire Cohort or Super-responder cohort, in a single or ≥2 platform measurements**<br>1. R vs. NR P <0.05 OR<br>2. R change vs. baseline P <0.05 AND NR change vs. baseline P >0.10<br><br>AND
**Tier III: Evidence of network behavior in human heart**	**A. Correlation with another β_1-GSN's member's expression profile**<br>1. Spearman's Rho of ≥0.50 when plotted against another gene's change from baseline expression in BORG Responders.

Table S2. Ventricular myocardial mRNA expression of genes in transgenic (Tg) mice overexpressing the Arg389 or Gly389 variant of the human *ADRB1* (β_1-adrenergic receptor, β_1-AR) gene. A. Tg mice mRNA abundance by microarray. B. Literature curated genes and total β_1-GSN membership.

**A. Tg Mice β_1-AR overexpressors**	**3 Months Age, TG1[7]**		**3 or 6-8 Months Age, TG2[8]**	
Array probe sets/unique transcripts identified →	*45,000/21,814		†28,000/24,009	
**Condition:**	**Direction of mRNA change vs. NTg controls**			
	**Upregulated, 3 months TG1 mice**	**Downregulated, 3 months TG1 mice**	**Upregulated, 3/6-8 months TG2 mice**	**Downregulated, 3/6-8 mos TG2 mice**
‡Genes with mRNA P<0.05 vs. NTg controls in ≥2	370	1199	434	403

conditions, *ADRB1* Arg and Gly389, same directional change				
Unique, TG1/TG2 concordant genes qualifying for regulation by β_1-AR signaling	Only TG1: 318<br>Also in TG2: 52	Only TG1: 1037<br>Also in TG2: 162	Only TG2: 382<br>Also in TG1: 52	Only TG2: 241<br>Also in TG1: 162
**Condition:**	**Direction of mRNA change vs. Baseline with reverse remodeling (RR)[&]**			
	**↓ in RR, ↑ in TG1 mice**	**↑ in RR, ↓ in TG1 mice**	**↓ in RR, ↑ in, TG2 mice**	**↑ in RR, ↓ in TG2 mice**
Tg ***gene changes*** concordant[&] with LV reverse remodeling (RR) mRNA changes, total number	61	164	129	114
Total ***gene changes*** antithetical to RR changes	468			
Unique changes in 3m or 3/6-12	47	115	115	65
Common changes in both groups (T1/TG2 concordant)	14	49	14	49
Total ***number of genes*** exhibiting directionally antithetical changes to RR	342 unique changes + 63 common TG1/TG2 changes<br>= 405 total gene changes			
**Condition:**	**Genes downregulated in RR (concordant with upregulated in TG1, TG2 mice)**		**Genes upregulated in RR (concordant with downregulated in TG1, TG2 mice)**	
Total number of individual Tg ***genes*** concordantly changed with RR, combined Tg groups[¶]	176 (47_{TG1} + 115_{TG2} + 14_{common})		229 (115_{TG1} +65_{TG2} + 49_{common})	
Total number of Tg genes qualifying for β_1-GSN membership	405			
**B. Curated genes**	**Upregulated in model systems exposed to β_1-AR stimulation or down-regulated on exposure to β_1-blockade; downregulated in RR**		**Downregulated in model systems exposed to β_1-AR stimulation or up regulated on exposure to β_1-blockade; upregulated in RR**	
Curated genes from Table S4	14		11	
**C. Total of Tg and Curated**	**Downregulated, RR (concordant with upregulated in Tg mice & curation)**		**Upregulated, RR (concordant with upregulated in Tg mice & curation)**	
Total β_1-GSN membership by directional change in human LV RR	**190**		**240**	
Total number of genes in β_1-GSN	**430**			

*Affymetrix GeneChip Mouse Genome 430 2.0 Array; †Affymetrix GeneChip Mouse Genome 1.0 ST 1.0 ST Array; ‡Transgenic Biofilter; §NTg = Nontransgenic controls; &Concordant (pharmacologically) change means opposite directionality in Tg mouse vs. reverse remodel human myocardium; ¶Only 1 set of common changes are counted in tabulating the total number of individual changed genes in the combined 3 month (3m) and 3/6-8 month (3/6-12m) groups. β_1-AR = β_1-adrenergic receptor.

Table S3 (Excel file**).** Genes with mRNA abundance changes in transgenic mice: **A**, 3 months old (TG_1); **B**, 3 or 6-8 months (TG_2); **C.** concordance of changed gene expression between TG1, TG2.

Table S4. Literature curated genes whose mRNA or protein expression was quantitatively changed in myocardium or cardiac myocytes by β-agonist or -antagonist exposure of ≥4 hours; agonist directional change opposite and antagonist change in the same direction as in RR Responders vs. Nonresponders in BORG.

Gene #	Genes upregulated by a β-AR agonist or downregulated by a β-AR antagonist in model system myocardium or cardiac myocytes, and DOWNREGULATED on RR in BORG		Genes downregulated by a β-AR agonist or upregulated by a β-AR antagonist in model system myocardium or cardiac myocytes, and UPREGULATED on reverse remodeling in BORG	
	**Gene Symbol**	**Curated References**	**Gene Symbol**	**Curated References**
1	*ACTA1**,‡	Sucharov CC et al. DOI: 10.1016/j.yjmcc.2008.04.014	*ADRB1**,‡	Nanoff C et al. doi: 10.1097/00005344-198902000-00004; Sato Y et al. DOI: 10.1254/jjp.69.343
2	*CANX*†,‡	Chen L et al. DOI: 10.1002/jbt.20405; Cicek FA et al. DOI: 10.1007/s10863-014-9568-6	*ADRB2**,‡	Matthews JM et al. doi: 10.1007/BF00168760; Zhao M et al. doi: 10.1161/01.res.73.5.943
3	*EDN1*†,‡	Chang L et al. PMID: 15339387; Xu M et al. DOI: 10.1211/jpp.60.6.0009	*AQP7**,§	(Fasshauer M et al. doi: 10.1055/s-2003-39478
4	*EEF1A1**,§	Song B et al. doi: 10.1021/pr500835w	*CD36*†,‡	Yan et al. DOI: 10.1128/MCB.00229-15; Lam MPY et al. doi: 10.1172/JCI73787
5	*GDF11**,‡	2 models in 1 paper: Zhang XJ et al. doi: 10.3892/mmr.2019.10077	*CKM**,§	Hammerschmidt S et al. doi: 10.1016/s0925-4439(00)00070-3
6	*GNAI2*†,§	(Eschenhagen T et al. DOI: 10.1007/BF00184292	*CPT1B*†,§	Faulx MD et al. doi: 10.1111/j.1440-1681.2007.04531
7	*IL6*†,‡	Yin F et al. DOI: 10.1074/jbc.M211028200 Cha HN et al. doi: 10.4196/kjpp.2009.13.3.153	*CYC1**,§	Song et al. doi: 10.1021/pr500835w
8	*LGALS1*†,‡	Song B et al. doi: 10.1021/pr500835w; Fan J et al. doi: 10.1016/j.bbadis.2018.08.016.	*PLN**,‡	Stein B et al. doi: 10.1152/ajpheart.1996.270.6.H2021; Feng Y et al. DOI: 10.1111/j.1745-7254.2007.00650.x
9	*MMP2*†,§	Guo D et al. doi: 10.1161/CIRCRESAHA.110.229054	*PPP1R1A**,‡	El-Armouche A et al. doi: 10.1016/j.ejheart.2007.09.006; El-Armouche A et al. doi: 10.1093/cvr/cvn208
10	*NOS2*†,‡	Cha HN et al. doi: 10.4196/kjpp.2009.13.3.153; Krenek P et al. doi: 10.1093/eurjhf/hfn026;	*RYR2**,‡	Feng Y et al. DOI: 10.1111/j.1745-7254.2007.00650.x; Waters SB et al. doi: 10.3389/fphys.2013.00011
11	*NR4A1*†,‡	(Yan G et al. DOI: 10.1128/MCB.00229-15	*ATP2A2**,§	Stein B et al. doi: 10.1152/ajpheart.1996.270.6.H2021

12	*PRKCA*†,‡	Braun M et al. doi: 10.1097/00005344-200306000-00018; Somvanshi RK et al. DOI: 10.1016/j.bbamcr.2014.01.002		
13	*SLC8A1**,‡;	Golden et al. DOI: 10.1152/ajpheart.2001.280.3.H1376; Mani et al. doi: 10.1016/j.yjmcc.2009.11.007		
14	*SLC9A1**,‡	(Shibata M et al. doi: 10.1152/ajpheart.00483.2011		
	N = 14		N = 11	

*Concordant changes in ≥2 platforms in BORG ("Effects of Beta-blockers on Remodeling and Gene Expression" trial, NCT01798992); †single platform change in BORG; ‡qualified by 2 published studies and/or models employing β-AR agonists or -antagonists; §qualified by 1 published agonist/antagonist study plus 1 three months Arg389 or Gly389, or six months Gly389 transgenic mouse β_1-AR cardiac overexpression mRNA change.

Table S5. Genes upregulated or downregulated in Responders, by any platform at P <0.05 or within the β_1-adrenergic gene signaling network (β_1-GSN).

Gene Category	P <0.05 Any Platform*		β_1-GSN	
	**Upregulated, Number (%)**	**Downregulated, Number (%)**	**Upregulated Number (%)**	**Downregulated Number (%)**
Unique	2975 (100†)	3934 (100†)	240 (8.1†)	190 ((4.8†))
Concordant§ ≥2 platforms	197 (6.6†)	321 (8.2†)	60 (25‡)	67 (35‡)
Fisher's exact P value vs.:				
Any Platform Upregulated	–	0.016	<0.0001	–
Any Platform Downregulated	0.016	–	–	<0.0001
β_1-GSN Upregulated	<0.0001	–	–	0.025

*by RT-PCR of 50 candidate genes in the BORG Entire Cohort (EC) (5), microarray for global gene expression in the EC, or RNA-sequencing in the SRC; †based on number of any-platform genes; ‡based on number of β_1-GSN genes; §same directional change in ≥2 platforms/total number of genes measured by any platform.

Table S6 (Excel file)**.** β_1-GSN gene list (N=430) by VMO Biologic Category: **A** (Sheet 1), Downregulated in Tg, upregulated in reverse remodeling; **B** (Sheet 2), Upregulated in Tg, downregulated in reverse remodeling.

Table S7. Upregulated vs. Downregulated genes, fatty acid, glucose, branched chain amino acid, electron transport and miscellaneous metabolic pathways.

Upregulated (function)	Gene (protein Localization*)	Category	Fold change	P value† vs. Downregulated
1 (enzyme)	*ACAA2* (M)	Beta oxidation	1.22	–
2 (enzyme)	*ACAD10* (M)	Beta oxidation	1.11	–
3 (enzyme)	*ACAD8* (M)	Beta oxidation	1.33	–
4 (enzyme)	*ACADM* (M)	Beta oxidation	1.35	–
5 (enzyme)	*ACADSB*(M)	Beta oxidation	1.30	–
6 (enzyme)	*ACADVL* (M)	Beta oxidation	1.33	–
7 (enzyme)	*CPT1B* (M)	Beta oxidation	1.18	–
8 (enzyme)	*DECR1* (M)	Beta oxidation	1.32	–
9 (enzyme)	*ECI1* (DCI) (M)	Beta oxidation	1.41	–
10 (enzyme)	*HADH* (M)	Beta oxidation	1.34	–
11 (enzyme)	*HADHA* (M)	Beta oxidation	1.31	–
12 (enzyme)	*HADHB* (M)	Beta oxidation	1.40	–
**Subtotal N**	12	Beta oxidation	1.30 ±0.09	12 vs. 0. 0.0005
13 (enzyme)	*ACSL1* (M, ER)	Other f.a. metabolism	1.39	–
14 (enzyme)	*ECHDC2* (M)	Other f.a. metabolism	1.35	–
15 (enzyme)	*ECHDC3* (M)	Other f.a. metabolism	1.44	–
**Subtotal N**	3	Other f.a. metabolism	1.39 ±0.05	3 vs. 1, 0.32
16 (transporter)	*ABCD2* (P)	Peroxisome f.a. metabolism	1.57	–
17 (enzyme)	*PHYH* (P)	Peroxisome f.a. metabolism	1.28	–
18 (transporter)	*SCP2* (P)	Peroxisome f.a. metabolism	1.30	–
19 (enzyme)	*ECH1* (P)	Peroxisome f.a. metabolism	1.34	–
20 (enzyme)	*HSDL2* (P)	Peroxisome, regulatory factor in lipid metabolism	1.35	–
**Subtotal N**	5	Peroxisome f.a./lipid metabolism	1.37 ±0.12	5 vs. 0 0.046
21(enzyme)	*AUH* (4 (M)	Branched-chain amino acid metabolism	1.34	–

22 (enzyme)	*BCKDHA* (M)	Branched chain a.a. met.	1.39	−
23 (enzyme)	*HIBADH* (M)	Branched chain a.a. met.	1.23	−
24 (enzyme)	*MCCC1* (M)	Branched chain a.a. met.	1.32	−
**Subtotal N**	4	Branched chain a.a. met.	1.32 ±0.07	4 vs. 0 0.046
25 (enzyme)	*ACAT1* (M)	TCA cycle	1.24	−
26 (enzyme)	*CS* (M)	TCA cycle	1.24	−
27 (enzyme)	*IDH2* (M)	TCA cycle	1.21	−
28 (enzyme)	*IDH3B* (M)	TCA cycle	1.31	−
29 (enzyme)	*MMAB* (M)	TCA cycle	1.33	−
30 (enzyme)	*MUT* (M)	TCA cycle	1.33	−
31 (enzyme)	*SUCLA2* (M)	TCA cycle	1.31	−
**Subtotal N**	7	TCA cycle	1.28 ±0.05	7 vs. 0 0.008
32 (enzyme)	*DLAT* (M)	Glycolysis	1.30	−
33 (enzyme)	*PDHB* (M)	Glycolysis	1.27	−
34 (enzyme)	*PDK2* (M)	Glycolysis	1.44	−
35 (enzyme)	*PDP1* (M)	Glycolysis	1.39	−
36 (enzyme)	*PANK4* (M)	Glycolysis	1.14	
37(enzyme)	*PFKM* (C)	Glycolysis	1.31	−
**Subtotal N**	6	Glycolysis	1.31 ±0.10	6 vs. 3 0.32
38 (enzyme)	*COQ3* (M)	Electron transport chain	1.37	−
39 (enzyme)	*COQ9* (M)	Electron transport chain	1.17	−
40 (enzyme)	*COX6C* (M)	Electron transport chain	1.22	−
41(cytochrome)	*CYC1* (M)	Electron transport chain	1.39	−
42 (enzyme)	*ETFB* (M)	Electron transport chain	1.34	−
43 (enzyme)	*ETFDH* (M)	Electron transport chain	1.35	−
44 (enzyme)	*NDUFA5* (M)	Electron transport chain	1.31	−
45 (enzyme)	*NDUFC2* (M)	Electron transport chain	1.25	−
46 (enzyme)	*NDUFS1* (M)	Electron transport chain	1.32	−
47 (enzyme)	*NDUFS2* (M)	Electron transport chain	1.05	−

48 (enzyme)	*NDUFS4* (M)	Electron transport chain	1.36	–
49 (transporter)	*NIPSNAP2* (M)	Electron transport chain	1.26	–
50 (enzyme)	*NUDT13* (M)	Electron transport chain	1.31	–
51 (enzyme)	*SDHA* (M)	Electron transport chain	1.21	–
52 (enzyme)	*SDHD* (M)	Electron transport chain	1.29	–
**Subtotal N**	15	Electron transport chain	1.28 ±0.09	15 vs. 1 0.0004
**Subtotal** N = 52	45M, 1C, 5P, 1ER	Upregulated genes for fatty acid, glucose, branched chain amino acid, electron transport	1.31 ±0.09	52 vs. 5 <0.0001
		Miscellaneous metabolic functions		
53 (transporter)	*CD36* (S)	Sarcolemmal f.a. transport	1.33	–
54 (enzyme)	*DHRS7C* (SR)	SR dehydrogenase/reductase, ?Ca^{2+} handling	12.6	–
55 (enzyme)	*ADHFE1* (M)	Oxidizes 4-hydroxybutyrate	1.29	–
56 (enzyme)	*ADK* (C)	Regulates concentration of adenosine	1.33	–
57 (enzyme)	*ALDH2* (C)	Metabolizes alcohol	1.34	–
58 (enzyme)	*LCLAT1* (C))	An acyl group transferase	1.35	–
59 (enzyme)	*AS3MT* (C)	Transfers CH3s from SAM to arsenicals	1.31	–
60 (enzyme)	*DGAT2* (C)	Catalyzes binding of DAG to long chain acyl CoA	1.88	–
61 (enzyme)	*OPLAH* (C)	Catalyzes formation of glutamate from L-proline	1.29	–
62 (enzyme)	*PM20D1* (C)	Generates N-acyl amino acids from f.a. or amino acids	1.36	–
63 (enzyme)	*ENTPD6* (C)	An NTPase	1.12	–
64 (enzyme)	*PCMT1* (C)	Catalyzes deamidation of aspartyl species to L-aspartyl	1.27	–
65 (enzyme)	*PCMTD2* (C)	Novel inhibitor of mammalian sterile 20-like kinase 1, which causes myocyte apoptosis and a dilated cardiomyopathy	1.35	–
66 (enzyme)	*ZADH2* (C)	Oxidoreductase and acyl transferase activities	1.31	–
67 (enzyme)	*CKM* (C)	Transfers a phosphate between ATP and creatine phosphate	1.35	–
**Subtotal N = 15**	12C, 1M, 1S, 1SR	Miscellaneous metabolic functions	2.10 2.91	15 vs. 11 0.38
**Grand Total: 67**	(46M; 5P; 13C; 1ER; 1S, 1SR)	All Upregulated metabolism genes	1.48 ±1.38	67 vs. 16 <0.0001

(62 enzymes, 4 transporters, 1 cytochrome)				

**Downregulated (function)**	**Gene**	Category	**Fold change**	**P value vs. Upregulated**
1 (enzyme)	*HK1* (M,C)	Glycolysis	0.83	–
2 (enzyme)	*PFKP* (C)	Glycolysis	0.91	–
3 (enzyme)	*PGAM1* (C)	Glycolysis	0.73	–
**Subtotal N**	3	Glycolysis	0.82 ±0.09	3 vs. 6 0.32
4 (enzyme)	*FADS1* (M,ER)	Other f.a. metabolism	0.98	1 vs. 3, 0.32
5 (protein assembly)	*ATPAF1* (M)	Electron transport chain	0.78	1 vs. 15 0.0004
**Totals: 5** 4 enzymes, 1 protein assembly	(1M, 1M/C; 2C, 1M/ER)	Downregulated genes for fatty acid, glucose, branched chain amino acid, electron transport	0.85 ±0.10	5 vs. 52 <0.0001
Miscellaneous metabolic functions				
6 (enzyme)	*ALDH18A1* (M)	Catalyzes reduction of glutamate in the biosytnesis of proline, arginine andornithine	0.69	–
7 (transporter)	*SFXN3* (M)	Serine transporter required for 1-carbon metabolism	0.72	–
8 (enzyme)	*RDH13* (M)	Catalyzes the reduction and oxidation of retinoids	0.73	–
9 (enzyme)	*CYB5R3* (C)	NADH-dependent enzyme that converts methemoglobin to hemoglobin	0.70	–
10 (enzyme)	*CYP1B1* (C)	A cytochrome P450 enzyme	0.70	–
11 (enzyme)	*UCK2* (C)	A pyrimidine ribonucleoside kinase (uridine->UMP & CMP)	0.65	–
12 (transporter)	*GM2A* (C)	A Glycolipid transporter protein	0.75	–
13 (enzyme)	*B4GALT5* (C)	Glycoprotein enzyme of uncertain function	0.90	–
14 (enzyme)	*SORD* (C)	Catalyzes the interconversion of polyols in the sorbitol pathway	0.73	–

15 (enzyme)	*STS* (ER)	Catalyzes several 3-beta-hydroxysteroid precursors for estrogens, androgens and cholesterol	0.69	–
16(enzyme)	*APOE* (M,P,N)	Major apoprotein of the chylomicron	0.69	–
**Subtotal: 11**	6C, 3M, 1ER, 1 M/P/N	Miscellaneous metabolic functions	0.72 ±0.06	–
**Grand Total: 16** (62 enzymes, 4 transporters, 1 cytochrome)	(8C, 4M; 1 ER, 1M/C, 1M/ER, 1M/P/N	All Downregulated metabolism genes	0.76 ±0.09	

*M = Mitochondria, C = Cytosol, ER = Endoplasmic reticulum, P = Peroxisome, S = Sarcolemma, SR = sarcoplasmic reticulum, N = nucleus; †Chi Square 1x2 test of absolute numbers of upregulated vs. downregulated within category.

Table S8. Subcategories of genes in the Gene Regulation category.

Gene Regulation	Upregulated in reverse remodeling (N)	Downregulated in reverse remodeling (N)	1x2† P value	2x2* P value*
Transcription regulation	(15) *CDK19, GCOM1, HDAC4, JARID2, MED4, MEIS2, PCF11, RBL2, RFXAP, RSAD1, SAFB2, TARDBP, TCEA3, VEZF1, ZFP30,*	(3) *NELFE, ZNF462, PNRC1*	0.005	0.53
Transcription factors	(7) *ATF7IP, CREBZF, HLF, KLF9, NR1D2, RCOR2, RXRG*	(2) *KLF7, NR4A1*	0.096	0.98
mRNA processing or stability	(8) *CLK1, HNRNPA1, HNRNPA2B1, HNRPDL, HNRNPM, SFRS11, SON, SYF2*	(0)	0.005	0.11
mRNA splicing regulation	(2) *RBM20, RBFOX1*	(1) *SRPK2*	0.56	0.62
Translation regulation	(4) *THUMPD1, PAIP2B, CPEB3, PET112L*	(4) *DHX32, GARS, PABPC1, EEF1A1*	1.00	0.040
Ribosomal protein	(5) *MRPL9, MRPs23, MRPs9, RPL15, RPL22*	(1) *RPL37*	0.10	0.75
DNA repair, stability or synthesis	(6) *REV1, REV3L* (mito), *EEPD1, SIRT5, SWIM7, RRM2B* (mito)	(1) *INTS3*	0.059	0.61
Chromatin/Histone regulation	(1) *CHD2*	(1) *BRD4*	1.00	0.33
DNA methylation, other epigenetic regulation	(2) *N6AMT1, N6AMT2*	(1) *CDK2AP1*	0.56	0.62
Total	(50)	(14)	<0.0001	

Chi square 1x2 test of upregulated vs. downregulated genes in individual subcategories; †Chi Square 2x2 test of individual subcategory against all other subcategories; mito = mitochondria.

Table S9. Subcategories of upregulated vs. downregulated **A.** Channels/Solute Exchangers and **B.** Cell Homeostasis genes.

**Category**	**Upregulated in Reverse Remodeling (N)**	**Downregulated in Reverse Remodeling (N)**	**1x2* P value**	**2x2† P value**
**A. Channels and Solute Exchangers**	**(12)** *HCN4,ANOS,CLCN3,KCNJ11,RNF207,SLC27A1, SLC41A1,SLC46A3,KCND3,SLC15A2,SLC25A26,AQP7*	**(4)** *CLCN3,SLC25A5,SLC38A3, SLC9A1*	0.046	–
**B. Cell Homeostasis**				
Golgi, ER, membrane and cytosol trafficking, protein folding and degradation	**(14)** *ABCA1, CELSR2, GGCT, IGSF1, ABCC9, ARIH2, BPHL, CHPT1, FBXO3, FRMD4A, GJA3, STX17, POMP, BOD1*	**(32)** *ABHD2, ANXA2, ARPC3, HSP90AA1, HSP90B1, PAM, PDIA4, PDLIM5, PICALM, CENPF, GOLIM4, HEXB, MAGED2, PACRG, SEC61A1, SERPINE2, SHROOM3, STK39, TMED3, GPX3, KRT80, MFAP5, TRAK2, AMFR, ANKIB1, CALU, CDC26, FTH1, GLTP, KLHL13, SEPX1, SIAH2*	0.008	0.002
Mitochondrial integrity	**(6)** *MRPS25, NNT, OMA1, CLPX, GCSH, GPAM*	**(2)** *ARMCX3*, GPX8	0.16	0.031
Peroxisome integrity	**(3)** *PRDX5, PXMP2, PEX7*	**(0)**	0.083	0.031
**Total Cell Homeostasis**	**(23)**	**(34)**	**0.16**	_

Chi square 1x2 test of upregulated vs. downregulated genes in individual subcategories; †Chi Square 2x2 test of individual subcategory against all other subcategories.

Table S10. A. Subcategories of genes in the Non-β_1-AR/cAMP Signaling pathways; **B.** Immune Function, Vascular/thrombosis and Unclassified categories.

**A. Signaling Pathways Other than β-adrenergic/cAMP/PKA**	**Upregulated in Reverse Remodeling (N)**	**Downregulated in Reverse Remodeling (N)**	**1x2* P value**	**2x2† P value**
Phosphoinositide, phospholipase or lysophosphatidic acid	**(6)** *PLCD3, PLCL2, PPAP2B, PPAPDC3, ARAP2, PIK3IP1*	**(2)** *PLCG2, SH3D19*	0.16	0.24
Non-β-adrenergic Neurohormonal	**(5)** *NR3C2, P2RY1, ADRA1A, AGTR1, ART3*	**(3)** *GNG12, CMKLR1, PDE4B*	0.48	0.68
Cytokines/Downstream Signaling	**(7)** *KLHL21, TRAP1, IL15, ASB10, ASB14, ASB15, ASB5*	**(8)** *TRAF4, CXCL16, IL1R1, TNFAIP6, SOCS2, C1QTNF6, IL6, NOS2*	0.80	0.43
Other Signaling Pathways	**(9)** *GPR116, HOMER2, SYCP3, FRMD5, EGF, ITGB6, PLXNB1, PPFIBP2, PRKCQ*	**(10)** *EGLN3, SRR, YWHAB, LAD1, S1PR1, ZDHHC2, ATRNL1, PAK1, PPM1E, PRKCA*	1.00	0.39
Small GTPases, Regulators	**(11)** *ARHGAP9, DOCK8, GPSMI, NKIRAS1, RAB12, RHOT1, TBC1D4, DENND4B, RASD2, RICS, RIC8B*	**(7)** *ARF4, DOCK1, PRAF2, RABGEF1, RAB23, RASSF2, RAB3IP*	0.49	0.59
AKAP related	**(1)** *AKAP8*	**(2)** *AKAP13, AKAP2*	0.56	0.43
**Total**	**39**	**32**	**0.34**	–
**B. Immune Function, Vascular/ thrombosis, Unclassified**	**Upregulated in Reverse Remodeling (N)**	**Downregulated in Reverse Remodeling (N)**	**1x2* P value**	**2x2† P value**
Immune function	**(5)** *B3GALT2, RHD, CD247, JAM2, QSOX2*	**(4)** C5AR1, CD44, CD55, THY1	0.74	0.70
Vascular/thrombosis	**(1)** *ANGPT1*	**(2)** *SEMA4A, PROS1*	0.56	0.30
Unclassified/Unknown function	**(11)** *KLHDC1,SH3RF2,TMEM182,MLF1, CCRN4L ,SPAG7,SUV420H1,TTC32,ALG10B,ZNF839*	**(5)** *AMMECR1,PRSS23,PDPN,FAM122B*	0.13	0.31
**Total**	**(17)**	**(11)**	**0.26**	**–**

*Chi square 1x2 test of upregulated vs. downregulated genes in individual subcategories; †Chi Square 2x2 test of individual subcategory against all other subcategories.

Table S11. Baseline (0 months), 3 months and 12 months data for the BORG study Entire Cohort used in Nonparametric Permutation Testing (N=46), Responders (R_{EC}) and Nonresponders NR_{EC}).

Parameter	Baseline	3 Month	12 Month	†Linear trend P
**Responders**	**n=30**	**n=30**	**n=26**	
LV ejection fraction (LVEF) (%)	25.9±8.1	40.9±11.6**	47.8±9.8**	<0.0001
LV end systolic volume (ESV) (ml)	168±72.5	99.4±59.5**	73.5±39.2**	<0.0001
LV end diastolic volume (EDV) (ml)	220±82.5	165±73.2**	136±54.1**	0.0002
RV ejection fraction (RVEF) (%)	27.2±8.8	32.4±10.7*	37.4±7.0**	0.0001
Heart rate (HR, bpm)	87.2±21.0	70.3±13.6**	68.8±10.2**	<0.0001
SBP, mmHg	106±12.8	113±15.2*	116±21.2*	0.030
PWP, mean mmHg)	11.3±8.7	9.5±5.4	7.2±5.1*	0.025
RAP, mean (mmHg)	4.1±3.6	4.6±3.8	4.0±4.2	0.91
Norepinephrine (NE, pg/ml)	452±266	430±426	391±240	0.52
NYHA, I/II/III/IV (%)	0/50/50/0	3/69/28/0*	31/42/27/0*	0.001
BMI (kg/m^2)	28.9±6.3	30.0±6.4*	31.7±6.8**	0.12
Cr Clearance (CrCl) (ml/min)	85.3±18.8	86.3±19.9	88.0±26.0	0.65
**Nonresponders**	**n=16**	**n=16**	**n=13**	**†Linear trend P**
LVEF (%)	27.8±9.9	30.8±12.8	30.0±10.2	0.58
LV ESV (ml)	183±92.8	171±99.0	193±70.0	0.86
LV EDV (ml)	255±111	236±105	264±81.8	0.90
RVEF (%)	27.2±9.7	31.3±11.5	33.1±12.9	0.21
HR (bpm)	79±20.2	73.6±13.8	76.4±19.0	0.66
SBP, (mmHg)	109±16.8	111±23.7	112±22.0	0.78
PWP (mean mmHg)	14.5±7.7	16.0±8.1	16.0±9.7	0.62
RAP (mean mmHg)	6.7±3.8	7.3±4.3	6.5±5.1	0.92
NE (pg/ml)	574±438	399±278	567±394	0.83
NYHA (I/II/III/IV (%)	0/62/38	27/53/20*	15/54/23/8	0.51
BMI (kg/m^2)	28.9±4.8	29.1±4.5	31.1±4.6*	0.23

CrCl (ml/min)	71.0±26.2	68.9±22.0	64.6±18.9	0.78
**All Subjects (Entire Cohort)**	**N=46**	**N=46**	**N=39**	
LVEF (%)	26.6±8.7	37.4±12.9**	41.9±13.0**	<0.0001
LV ESV (ml)	173±78.7	125±82.4**	104±71.2**	0.0006
LV EDV (ml)	232±92.7	190±91.3**	169±83.3*	0.006
RVEF (%)	27.2±9.0	32.0±10.9**	36.1±9.4**	0.0001
HR (bpm)	84.3±20.9	71.5±13.6**	71.3±14.0**	0.0003
SBP, (mmHg)	107±14.2	112±18.4*	114±21.3*	0.068
PWP (mean mmHg)	12.4±8.5	11.8±7.1	10.1±8.0	0.19
RAP (mean mmHg)	5.0±3.8	5.5±4.1	4.8±4.6	0.85
NE (pg/ml)	492±331	421±387	431±285	0.45
NYHA (I/II/III/IV (%)	0/53/47/0/	12/65/23/0*	26/45/26/3*	0.003
BMI (kg/m^2)	28.9±5.7	29.7±5.8*	31.5±6.1**	0.049
CrCl (ml/min)	80.2±22.5	80.9±21.8	80.9±26.2	0.90

*P <0.05 vs Baseline, paired Holm-Sidak test; **P <0.001 vs Baseline, paired Holm-Sidak test; †performed on unpaired values; PWP = pulmonary wedge pressure; BMI = Body Mass Index; NYHA = New York Heart Association functional class.

Table S12. Positive and negative Z_p values, and those that are statistically significant (≥1.96, ≤-1.96).

Time	$Z_p > 0$	$Z_p < 0$	$Z_p > 1.96$	$Z_p < -1.96$	*Significance, + vs - Z_p†*	*Significance, $Z_p > 1.96$‡*	*Significance, $Z_p < -1.96$‡*
Month 0	152 (60.3%)	100 (39.7%)	40 (15.9%)	5 (2.0%)	$\chi^2(1) = 10.73$, p = 0.0011	$\chi^2(1) = 184.89$, p< 0.0001	$\chi^2(1) = 0.28$, p = 0.5999
Month 3	170 (67.5%)	82 (32.5%)	58 (23.0%)	6 (2.4%)	$\chi^2(1) = 30.73$, p< 0.0001	$\chi^2(1) = 435.15$, p< 0.0001	$\chi^2(1) = 0.01$, p = 0.9037
Month 12	200 (79.4%)	52 (20.6%)	72 (28.6%)	1 (0.4%)	$\chi^2(1) = 86.92$, p< 0.0001	$\chi^2(1) = 702.73$, p< 0.0001	$\chi^2(1) = 4.57$, p = 0.0325

†: Comparison to Expectation 50%, 50%

‡: Comparison to Expectation 2.5%, 97.5%

Table S13. Phenotypic categories, number of gene ventricular myocardial ontology (VMO) measurements with Z_ps ≥1.96

Row	*BMI*	*CrCl*	*EDV*	*ESV*	*HR*	*LVEF*	*norepi*	*nyha*	*PWP*	*RAP*	*RVEF*	*SBP*	*Total*
M0	0	0	0	1	9	9	0	2	4	1	10	4	40
M3	1	1	8	9	2	12	1	1	14	2	7	0	58
M12	4	2	0	3	3	13	4	5	13	11	13	1	72
Overall χ^2 Test	$\chi^2(2)$ = 5.65, p = 0.059	$\chi^2(2)$ = 2.10, p = 0.35	$\chi^2(2)$ = 18.33, p = 0.0001	$\chi^2(2)$ = 10.08, p = 0.006	$\chi^2(2)$ = 7.90, p = 0.019	$\chi^2(2)$ = 1.66, p = 0.44	$\chi^2(2)$ = 5.65, p = 0.059	$\chi^2(2)$ = 3.72, p = 0.16	$\chi^2(2)$ = 11.56, p = 0.003	$\chi^2(2)$ = 16.71, p = 0.0002	$\chi^2(2)$ = 3.44, p = 0.18	$\chi^2(2)$ = 5.65, p = 0.059	$\chi^2(2)$ = 11.72, p = 0.003
Proportion Trend Test	$\chi^2(1)$ = 5.21, p = 0.022	$\chi^2(1)$ = 2.10, p = 0.15	$\chi^2(1)$ = 0.00, p = 1.00	$\chi^2(1)$ = 0.58, p = 0.45	$\chi^2(1)$ = 4.96, p = 0.026	$\chi^2(1)$ = 1.53, p = 0.22	$\chi^2(1)$ = 5.21, p = 0.022	$\chi^2(1)$ = 1.93, p = 0.16	$\chi^2(1)$ = 7.72, p = 0.006	$\chi^2(1)$ = 13.78, p = 0.0002	$\chi^2(1)$ = 0.86, p = 0.35	$\chi^2(1)$ = 2.93, p = 0.09	$\chi^2(1)$ = 11.66, p = 0.0006

Table S14. Z_p value summary and directional correlation of VMO categories vs. phenotype measurements (RAP and SBP not included, neither of which had statistically significant Z_p Friedman omnibus P values for differences in times 0 to 12). The calculation include only genes in a phenotypic correlation with at least 1 $Z_p \geq 1$ at either Month 0, 3 or 12.

Gene Category	Month 0				Month 3						Month 12					
	Abs/Z_p	Net (Color*)	↑ genes	↓ genes	Abs/Z_p	Net (Color*)	↑ genes	↓ genes	New	Lost c/t Prev	Abs/Z_p	Net (Color*)	↑ genes	↓ genes	New	Lost c/t Prev
	ESV				ESV (↓)											
MT	2.0	G	0	0	0.3					L	-0.2					
ECM/Fb	-1.4				6.9	B	0	+	N		1.5					L
Growth/Hty	1.2				4.2	G	0	+	N		1.2					L
Hmst	-0.3				3.6	G	−	+	N		1.7					L
Met	1.9				4.4	Y	−	+	N		2.7	Y	−	+		
Cytkns	-0.7				3.5	G	−	+	N		1.0					L
CAP	1.0				4.0	G	0	0	N		2.3	G	0	0		
Chn/ExCh	-0.2				2.9	G	−	0	N		1.9					L
βAR/PKA	0.1				3.0	G	−	+	N		3.1	G	0	+	N	
Gene Reg	1.5				2.7	Y	−	+	N		1.1					L
Median<br>25%, 75%	0.59<br>(-0.28,<br>1.40)				3.53<br>(2.89,<br>4.11)						1.58<br>(1.14,<br>2.22)					
P value*<br>vs. M0<br>vs. M3	0.003<br>−				 0.009						 0.008<br>0.009					
EDV					EDV (↓)											
ECM/Fb	-1.3				4.9	B	0	+	N		0.2					L
Growth/Hty	0.6				2.9	G	0	+	N		0.3					L
Hmst	-0.5				2.7	G	−	+	N		0.8					L
Met	1.3				2.9	Y	−	+	N		1.6					L
Cytkns	-0.8				2.1	Y	−	+	N		1.1					L
CAP	0.5				3.1	G	0	0	N		1.6					L
Chn/ExCh	-0.3				2.1	G	−	0	N		1.1					L
βAR/PKA	0.2				2.3	G	−	+	N		1.8					L
Median<br>25%, 75%	-0.04<br>(-0.59,<br>0.55)				2.78<br>(2.23,<br>2.96)						1.12<br>(0.65,<br>1.60)					
P value*<br>vs. M0<br>vs. M3	0.0008<br>−				 0.012						 0.016<br>0.012					

**LVEF**					**LVEF (↑)**											
Met	3.8	G	+	−	4.6	B	+	−	N		7.9	B	+	−		
Gene Reg	3.6	B	+	−	3.6	B	+	−			2.3	B	+	−		
βAR/PKA	3.8	G	+	−	4.0	G	+	−			4.0	G	0	−	N	
Growth/Hty	3.0	G	0	−	5.1	G	0	−			4.2	G	0	−		
Ca2+ Handl	2.9	G	0	0	1.6					L	2.2	G	0	0	N	
LAP	2.7	G	0	0	5.1	G	0	0			4.3	G	0	0		
ECM/Fb	3.8	Y	0	−	8.8	Y	0	−			5.0	Y	0	−		
Chn/ExCh	2.2	G	+	0	4.3	G	+	0			4.3	G	+	0		
Ctsk	2.6	G	0	0	1.6					L	1.7					
Hmst	1.1				5.1	G	+	−	N		4.5	G	+	−		
Cytkns	-0.7				4.2	G	−	+	N		1.9					L
PI3K/PLC	1.7				2.4	G	0	0	N		1.9					L
Non-β NH	-0.1				2.0	G	0	0	N		0.5					L
Immune	0.1				2.9	G	0	0	N		2.1	G	0	0		
Vasc/Throm	-1.5				-0.3						3.1	G	0	0	N	
Sm GTPases	1.0				1.952						2.3	G	+	−	N	
Median 25%, 75%	2.25 (-0.06, 2.99)				3.63 (1.95, 4.62)						3.08 (2.07, 4.25)					
P value* vs. M0 vs. M3	0.004 −				0.004						0.004 0.38					
**RVEF**					**RVEF (↑)**											
Growth/Hty	4.4	G	0	0	1.8					L	3.3	Y	0	−	N	
Ca2+ Hndl	3.3	G	0	0	2.3	G	0	0			0.9					L
Cytsk	2.7	G	0	0	1.3					L	-0.1					
Chn/ExCh	2.5	G	+	0	1.5					L	3.8	G	+	0	N	
Met	2.4	B	+	0	4.8	B	+	0			6.4	B	+	−	N	
Apoptosis	2.3	G	0	0	0.7					L	0.6					
Gene Reg	2.2	G	+	−	0.7					L	3.2	B	+	0	N	
βAR/PKA	2.2	B	+	−	2.3	G	+	0	N		2.7	G	0	−	N	
CAP	2.1	G	0	0	1.3					L	0.9					
UnCl/Ukn	2.0	G	+	0	0.7					L	2.2	G	0	0	N	
ECM/Fb	0.5				9.5	Y	0	−	N		6.6	Y	0	−		
Cytkns	1.2				4.2	G	+	−	N		0.7					
Sm GTPases	0.4				2.9	G	0	0			2.4	G	+	−	N	
Vasc/Throm	-0.9				-1.1						3.3	G	0	0	N	
PI3K/PLC	0.1				1.3						2.0	G	0	0	N	

Immune	1.2				0.5						4.7	G	0	0	N	
NonβAR NH	1.1				0.6						3.1	G	0	0	N	
Hmst	1.0				2.1	G	0	0	N		2.1	G	+	−	N	
Median<br>25%, 75%	2.04<br>(1.04,<br>2.38)				1.43<br>(0.69,<br>2.27						2.55<br>(1.20,<br>3.30					
P value*<br>vs. M0<br>vs. M3	0.90				– 						–<br>–					
		**PWP**									**PWP (↔/↓)**					
βAR/PKA	2.6	G	−	0	4.2	G	−	0			2.6	G	−	+	N	
Growth/Hty	4.2	G	0	+	6.3	B	0	+	N		3.4	G	0	+	N	
Hmst	3.2	B	0	+	5.6	G	−	+	N		4.2	G	−	+		
CAP	2.9	G	0	0	2.7	G	0	0			4.7	G	0	0		
ECM/Fb	-0.5				12.6	B	0	+	N		6.4	B	0	+		
Met	0.1				6.6	Y	−	+	N		6.8	Y	−	+		
Cytkn	1.9				4.1	G	0	0	N		1.7					L
Chnls/ExCh	1.2				5.1	Y	−	0	N		4.7	Y	−	0		
Gene Reg	1.4				4.7	Y	−	+	N		4.7	Y	−	+		
PI3K/PLC	0.1				2.2	G	0	0	N		0.7					L
Non β NH	-0.8				3.4	G	0	0	N		2.8	G	0	0		
Immune	0.1				4.8	G	0	0	N		2.0	G	0	0		
Cytsk	1.0				2.4	G	0	0	N		2.9	G	0	0		
Apopt	1.6				2.4	B	0	+	N		1.2					L
Ca2+ Handl	-2.1				0.5						3.9	G	0	0	N	
Other Signl	1.7				1.4						3.1	G	−	+	N	
Median<br>25%, 75%	1.27<br>(0.07,<br>2.05)				4.17<br>(2.39.<br>5.24)						3.26<br>(2.45,<br>4.67)					
P value*<br>vs. M0<br>vs. M3	0.005<br>−				0.0005						0.003<br>0.21					
		**Heart Rate**									**Heart Rate ↓**					
Met	3.7	Y	−	+	-2.1					L	1.7					
Gene Reg	5.4	Y	−	+	1.8					L	2.4	Y	−	+		
βAR/PKA	3.2	G	−	+	-0.3					L	0.9					
Growth/Hty	2.7	G	0	+	1.4					L	1.7					
Hmst	2.1	G	−	+	1.5					L	1.3					
Ca2+ Handl	2.3	G	0	0	0.3					L	0.9					

Apopt	2.6	G	0	0	1.5					L	3.6	B	0	+	N	
ECM/Fb	3.1	B	0	+	-2.5					L	2.7	B	0	+	N	
Cytkn	2.6	G	−	0	-0.3					L	0.6					
PI3K/PLC	0.5				2.6	G	0	0	N		-2.1					L
AKAP	-0.0				3.5	G	0	0	N		-0.5					L
Median<br>25%, 75%	2.57<br>(2.20,<br>3.13)				1.38<br>-0.29,<br>1.77)						1.28<br>(0.73,<br>2.22)					
P value*<br>vs. M0<br>vs. M3	0.009<br>−				 0.012						 0.015<br>0.32					
**NYHA**					**NYHA (↓)**											
ECM/Fb	6.2	B	0	+	-0.6					L	3.5	B	0	+		
AKAP	2.3	G	0	0	1.3					L	0.1					
Met	0.5				2.6	Y	−	0	N		2.4	Y	−	+		
Chnls/ExCh	-1.0				0.4						2.2	Y	−	0	N	
CAP	-0.5				0.8						3.2	G	0	0	N	
Hmst	1.4				1.5						2.6	G	−	+	N	
Median<br>25%, 75%	0.93<br>(-0.28,<br>2.06)				0.64<br>(0.38,<br>1.20)						2.54<br>(2.28,<br>3.09)					
P value*<br>vs. M0<br>vs. M3	0.61<br>−				 −						 −<br>−					
**Norepinephrine**					**Norepinephrine (↔) (effects (↓) due to β-blockade)**											
Grwth/Hty	0.1				2.0	G	0	+	N		0.3					L
Met	0.1				-1.0						2.0	Y	−	0	N	
ECM/Fb	-2.3				-2.3						3.1	B	0	+	N	
Chnls/ExCh	-0.4				-0.6						2.7	G	−	0	N	
Non-β NH	-1.1				-1.0						3.1	G	0	0	N	
Median<br>25%, 75%	-0.42<br>(-1.07,<br>0.14)				-0.96<br>(-0.99,<br>-0.56)						2.65<br>(2.03,<br>3.05)					
P value*<br>vs. M0<br>vs. M3	0.074<br>−				 −						 −<br>−					
**CrCl**					**CrCl (↔)**											
Non-β NH	1.1				2.2	G	0	+	N		1.7					L
βAR/PKA	-0.0				0.4						3.0	G	0	0	N	
Ca2+ Handl	1.0				0.5						2.2	G	0	0	N	

Median<br>25%, 75%	1.01<br>(0.49,<br>1.03)				0.52<br>(0.47,<br>1.36)						2.21<br>(1.95,<br>2.62)					
P value*<br>vs. M0<br>vs. M3	0.26<br>–				– 						 –<br>–					
**BMI**					**BMI (↑)**											
Other Signl	1.3				2.5	G	0	+	N		2.2	G	0	+		
ECM/Fb	-0.4				-2.3						4.8	B	0	+	N	
CAP	-0.6				-0.2						2.3	G	0	0	N	
Sm GTPases	0.1				1.2						2.4	G	0	0	N	
Median<br>(25%,<br>75%)	-0.19<br>(-0.48,<br>0.36)				0.48<br>(-0.74,<br>1.52)						2.34<br>(2.29,<br>2.97)					
P value*<br>vs. M0<br>vs. M3	0.11<br>–				– 						 –<br>–					

Color Coded Net correlation with phenotypic measure: B (Blue) = Direct, Y (Yellow) = Inverse; G (Gray) = No significant correlation; "N" in New column = Z_p is newly significant since the previous timepoint, or there has been a change in correlation directionality; "L" in Lost column means Z_p from the previous timepoint is no longer statistically significant; *P values are generated by Friedman tests for omnibus value (in top row, time 0), with Wilcoxon signed rank between groups followed by a Benjamini-Hochberg adjustment for false discovery (2nd and 3rd rows).

Table S15 (Excel file). All lncRNAs identified by NONCODE.

Table S16 (Excel file)**. A.** 121 β_1-GSN genes that are the closest proximity to lncRNAs change with reverse remodeling in Responders: **A**, Downregulated β_1-GSN that are the closest genes to changed lncRNAs; **B**. Upregulated β_1-GSN closest genes to changed lncRNAs. See xls spreadsheets.

10.0 Supplemental Figures

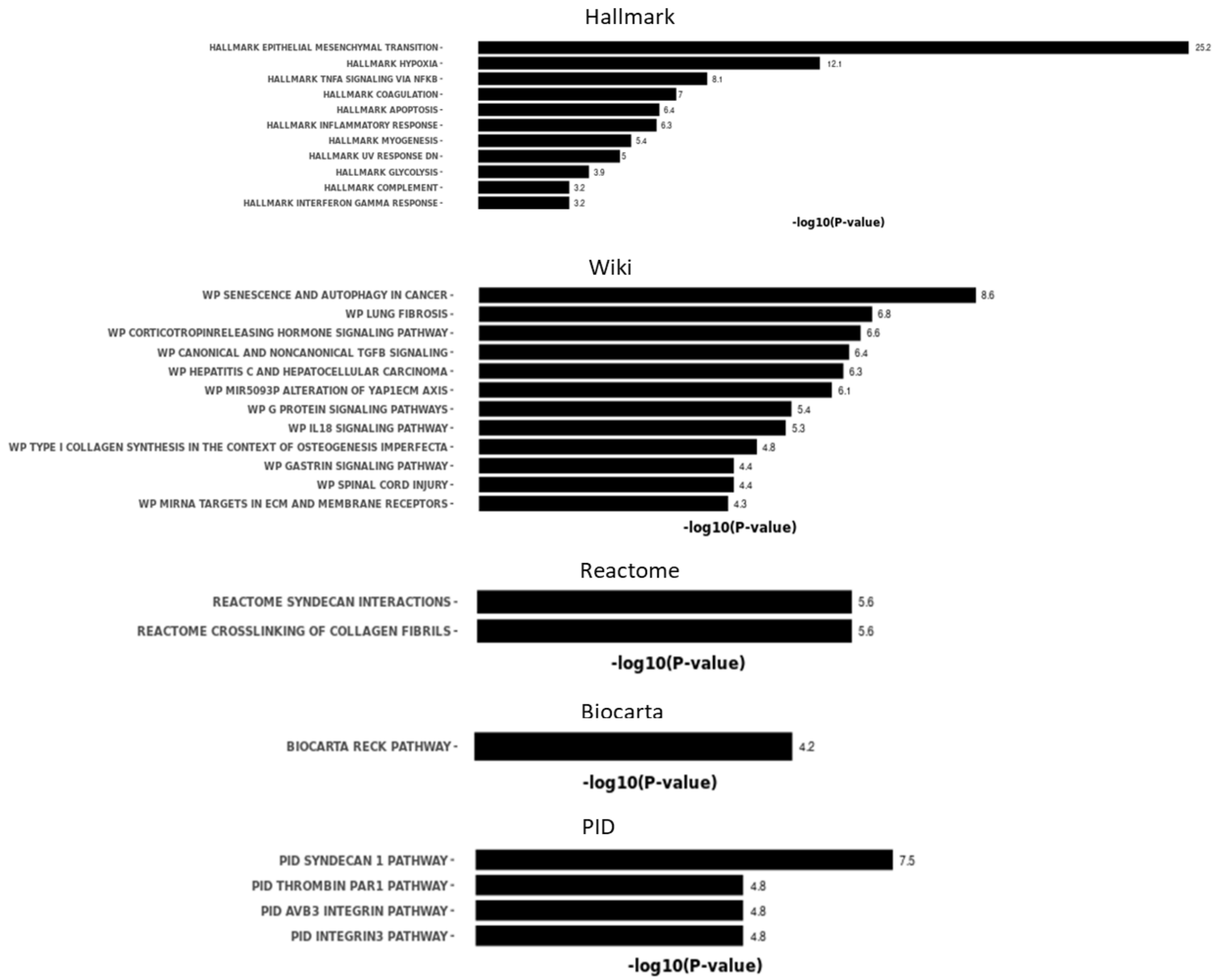

Figure S1. Canonical pathway enrichment of downregulated genes in the β_1-gene signaling network. -Log P values at the end of bars are from a modified Fisher's exact test to determine pathway enrichment, with a Benjamini-Hochberg correction for false discovery.

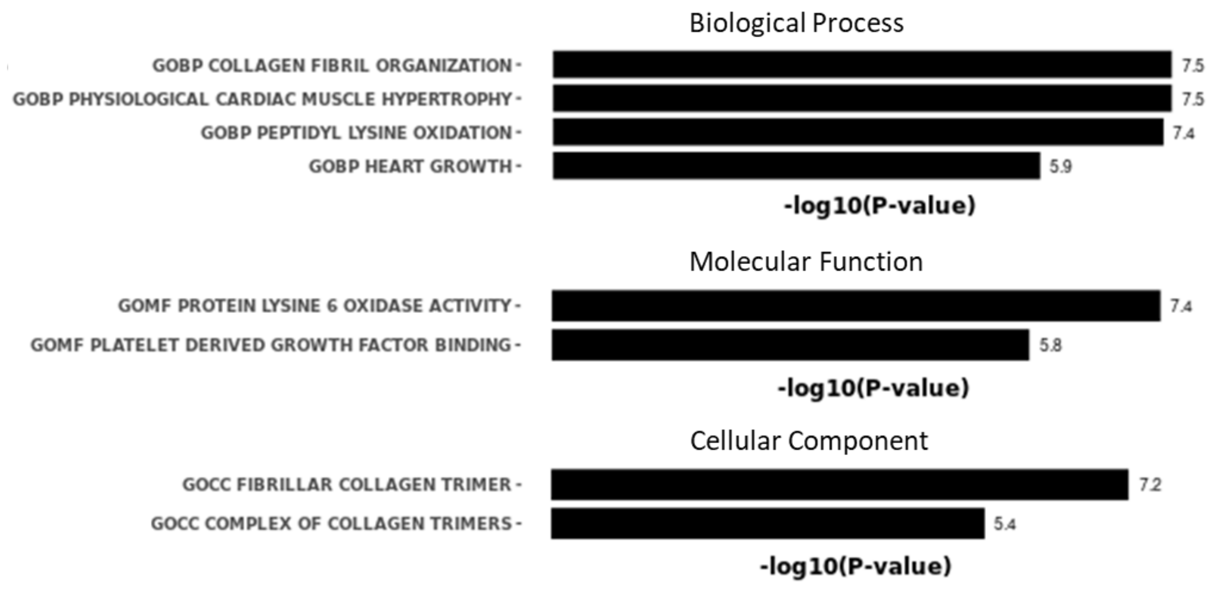

Figure S2. GO pathway enrichment of downregulated genes in the β_1-gene signaling network. -Log P values at the end of bars are from a modified Fisher's exact test to determine pathway enrichment, with a Benjamini-Hochberg correction for false discovery.

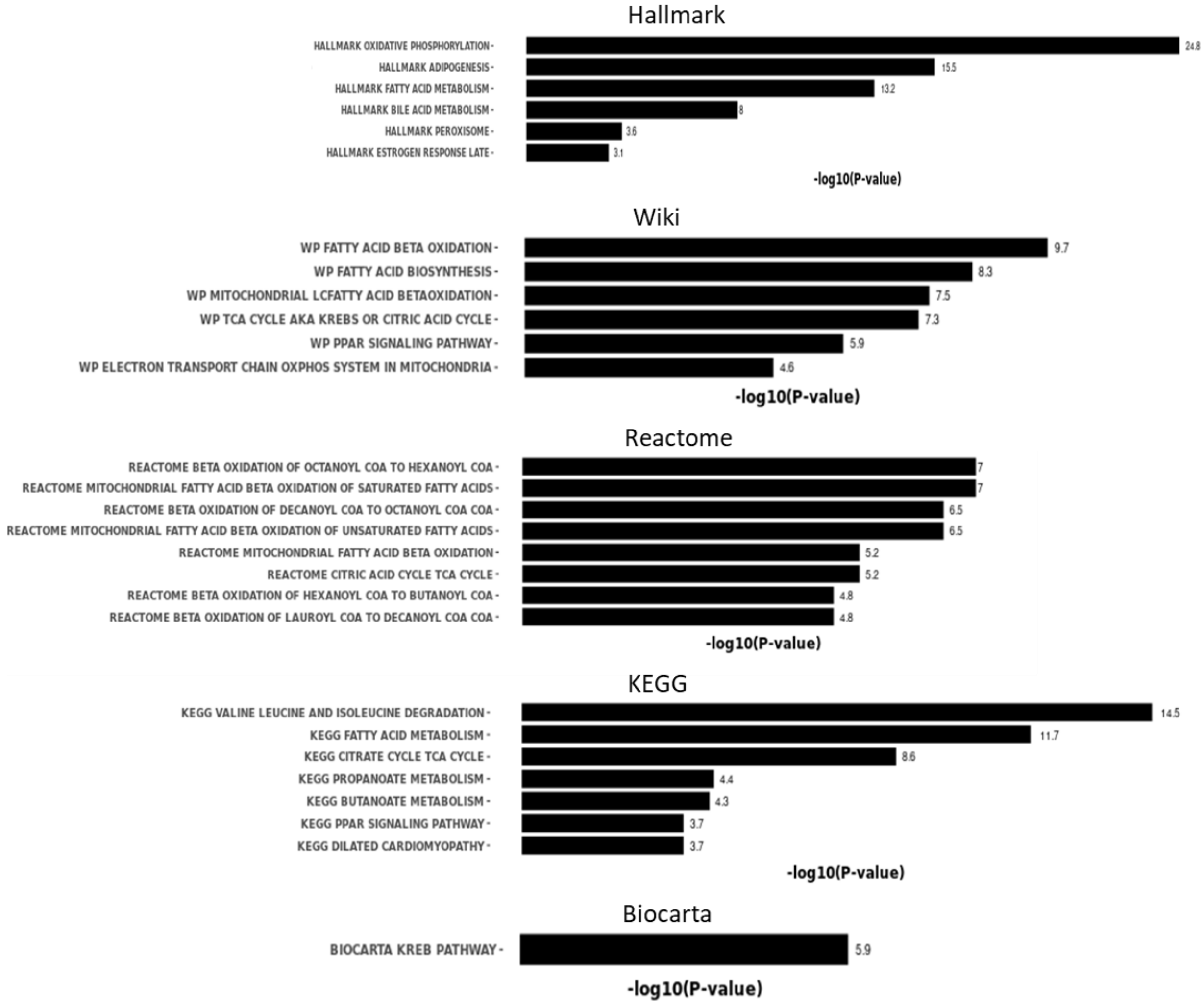

Figure S3. Canonical pathway enrichment of upregulated genes in the β_{1}-gene signaling network. -Log P values at the end of bars are from a modified Fisher's exact test to determine pathway enrichment, with a Benjamini-Hochberg correction for false discovery.

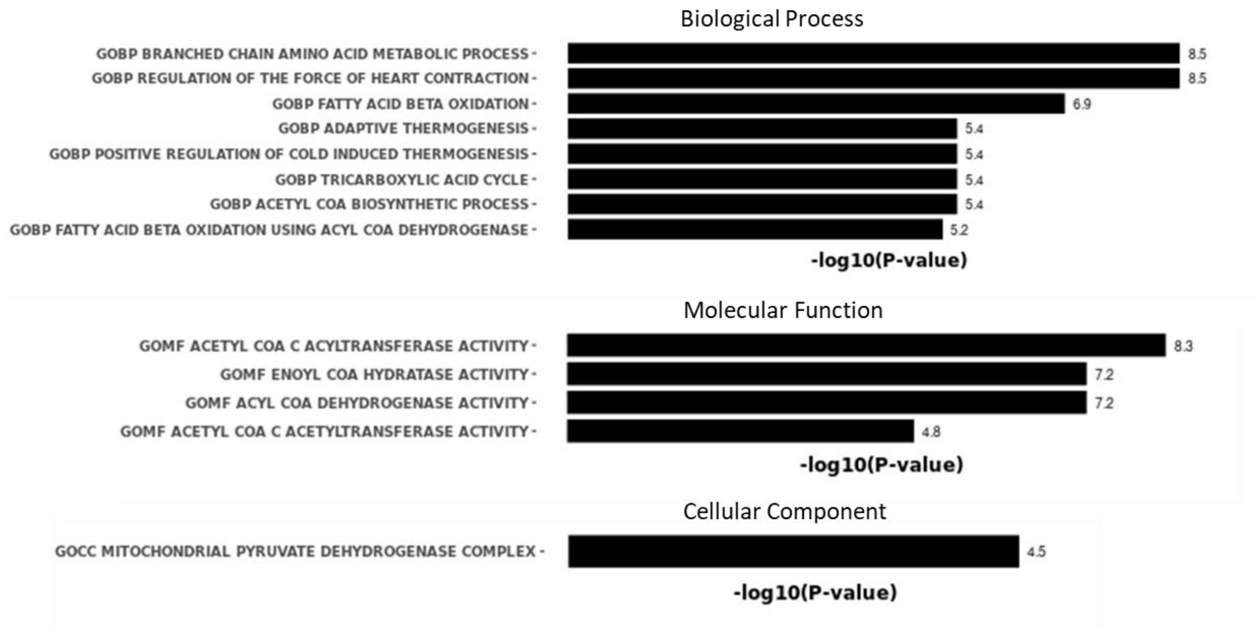

Figure S4. GO pathway enrichment of upregulated genes in the β_1-gene signaling network, -Log P values at the end of bars are from a modified Fisher's exact test to determine pathway enrichment, with a Benjamini-Hochberg correction for false discovery.

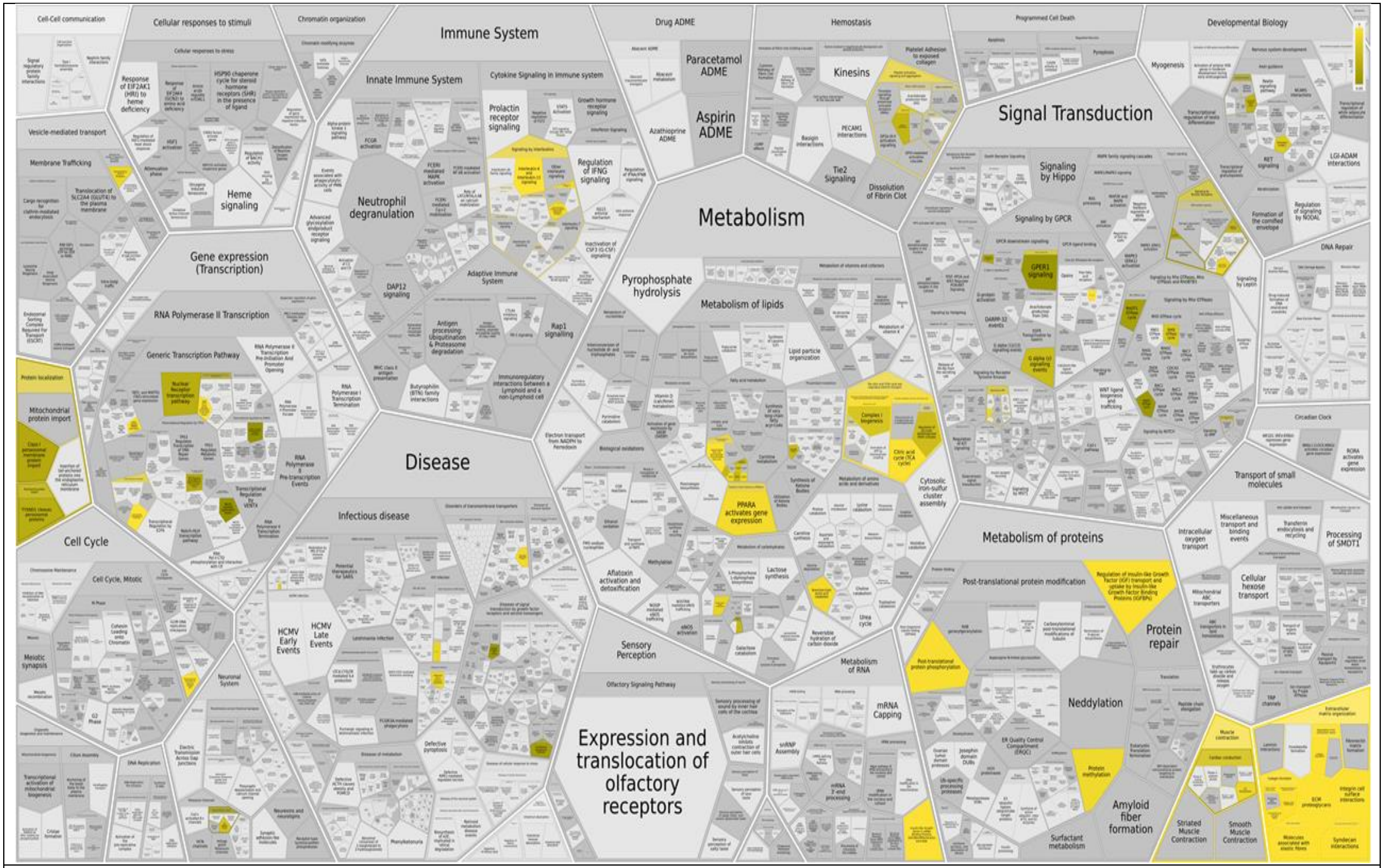

Figure S5 Reactome enrichment map of the β_1-ARgene signaling network.

A.

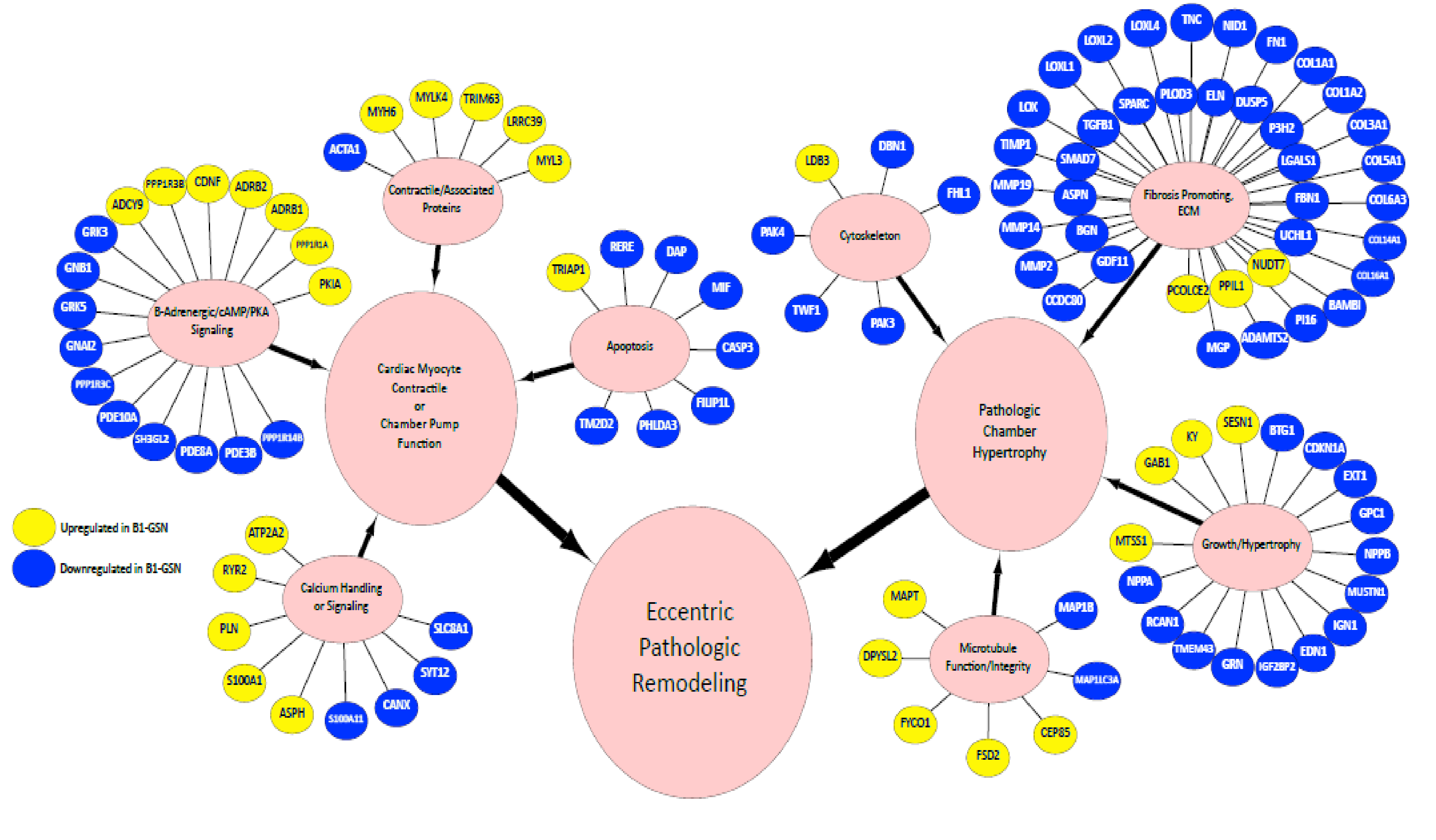

B-Adrenergic/cAMP/PKA Signaling
ADCY9
PPP1R3B
CDNF
ADRB2
ADRB1
PKIA
GRK3
GNB1
GRK5
GNAI2
PPP1R3C
PDE10A
SH3GL2
PDE8A
PDE3B
PPP1R14B
Contractile/Associated Proteins
ACTA1
MYH6
MYLK4
TRIM63
LRRC39
MYL3
Cardiac Myocyte Contractile or Chamber Pump Function
Apoptosis
TRIAP1
RERE
DAP
MIF
CASP3
FILIP1L
PHLDA3
TM2D2
Calcium Handling or Signaling
ATP2A2
RYR2
PLN
S100A1
ASPH
S100A11
CANX
SYT12
SLC8A1
Upregulated in B1-GSN
Downregulated in B1-GSN
Eccentric Pathologic Remodeling
Cytoskeleton
LDB3
DBN1
FHL1
PAK4
TWF1
PAK3
Fibrosis Promoting, ECM
LOXL1
LOXL2
LOXL4
TNC
NID1
FN1
COL1A1
COL1A2
COL3A1
COL5A1
COL6A3
COL14A1
COL16A1
BAMBI
PI16
ADAMTS2
MGP
PPIL1
PCOLCE2
NUDT7
UCHL1
FBN1
LGALS1
P3H2
DUSP5
ELN
PLOD3
SPARC
TGFB1
SMAD7
ASPN
BGN
GDF11
CCDC80
MMP2
MMP14
MMP19
TIMP1
LOX
Pathologic Chamber Hypertrophy
Growth/Hypertrophy
KY
SESN1
BTG1
CDKN1A
EXT1
GPC1
NPPB
MUSTN1
IGN1
EDN1
IGF2BP2
GRN
TMEM43
RCAN1
NPPA
MTSS1
GAB1
Microtubule Function/Integrity
MAPT
MAP1B
MAP1LC3A
CEP85
FSD2
FYCO1
DPYSL2

B.

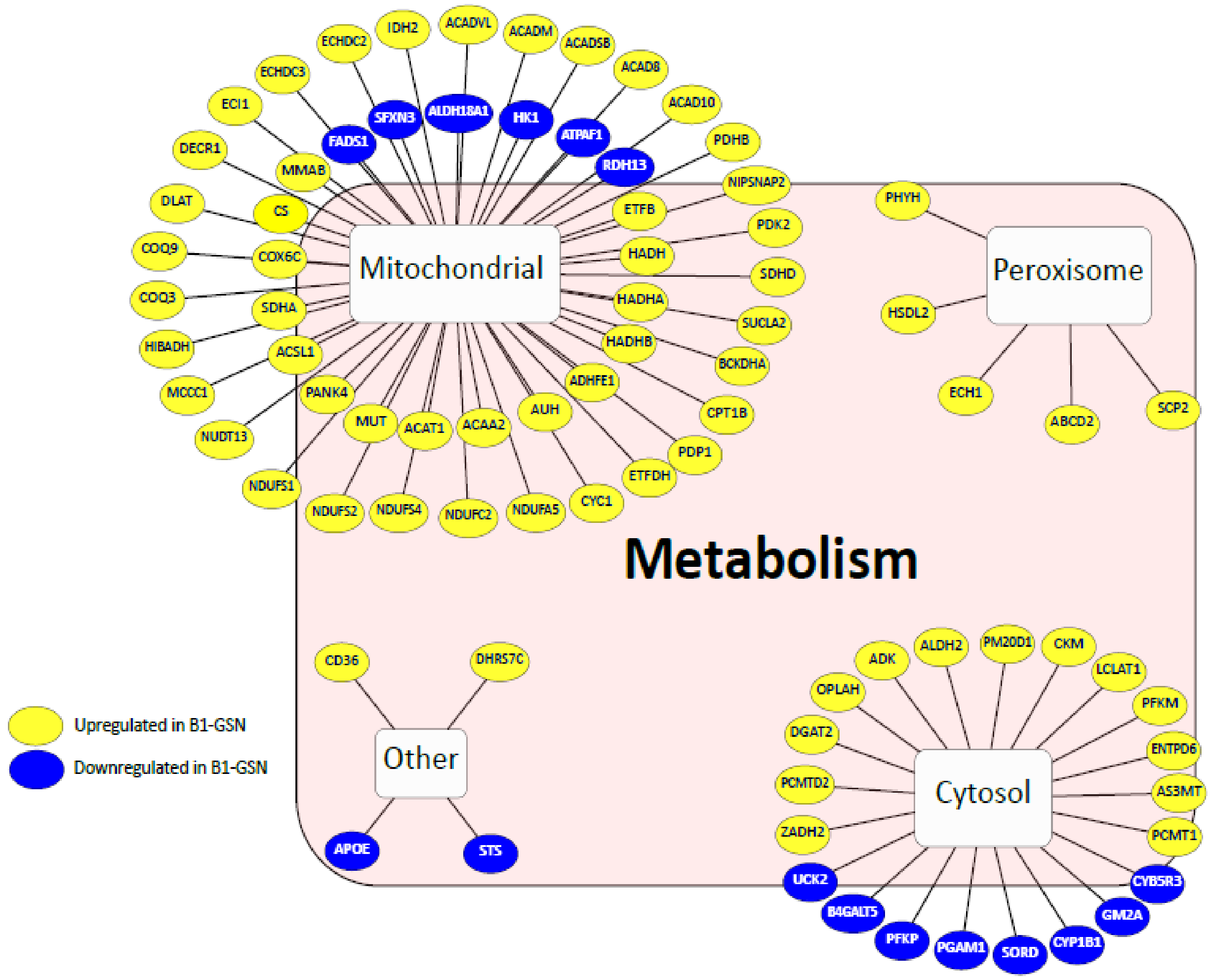

Figure S6. Cytoscape (version 3.10.0, https://cytoscape.org/download.html) (12) network modeling of β_1-GSN members within ventricular myocardial ontology (VMO) categories involved in **A.** eccentric pathologic remodeling and its reversal, and **B**. Metabolism.

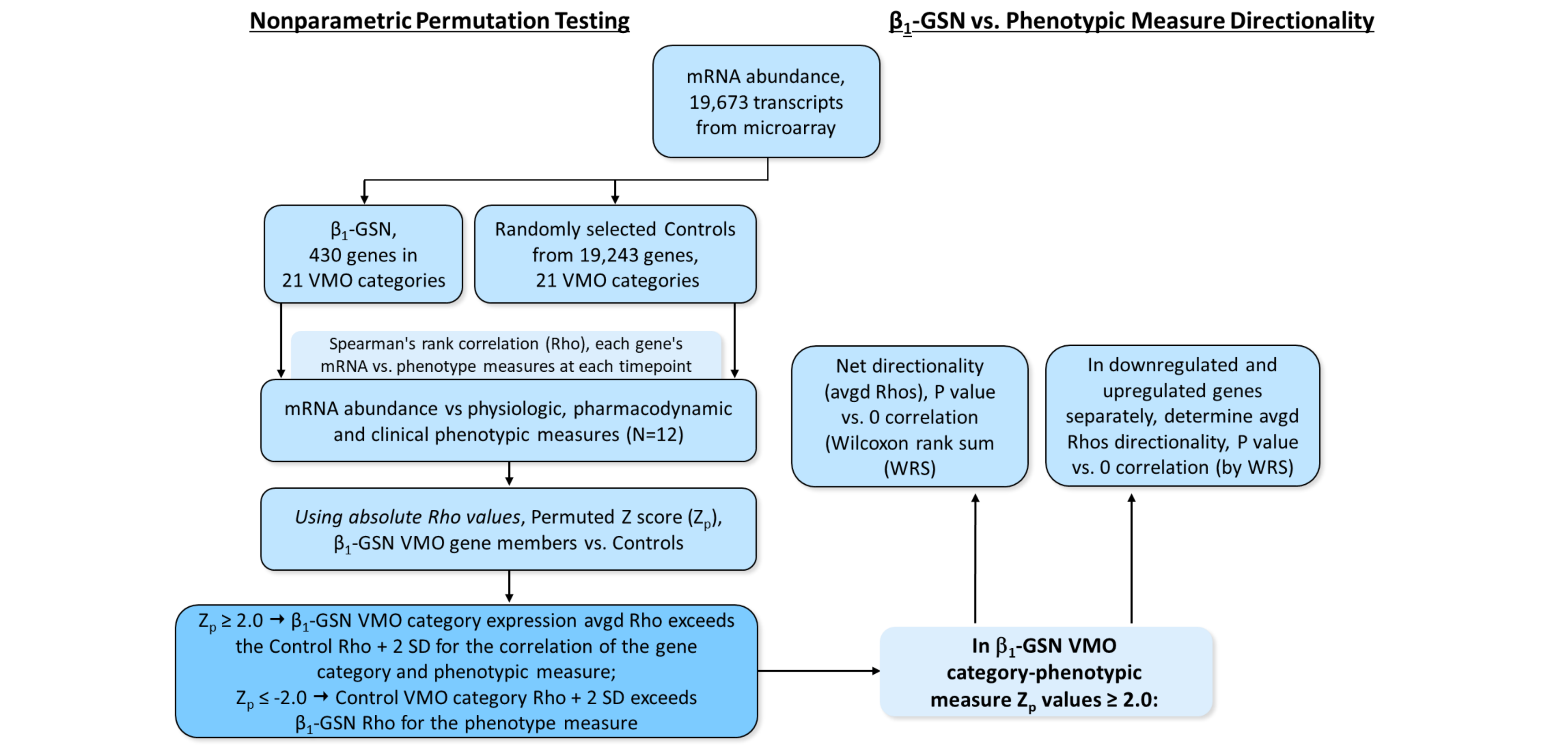

Figure S7. Scheme for Nonparametric Permutation Testing and generation of Z_p values for β_1-AR Gene Signaling Network (β_1-GSN) vs. non β_1-GSN Controls, and determination of directionality of β_1-GSN) Ventricular Myocardial Ontology (VMO) Categories vs. Phenotype measurements. Statistical significance for Z_p values is $\geq$1.96, rounded to 2.0 in heatmaps.

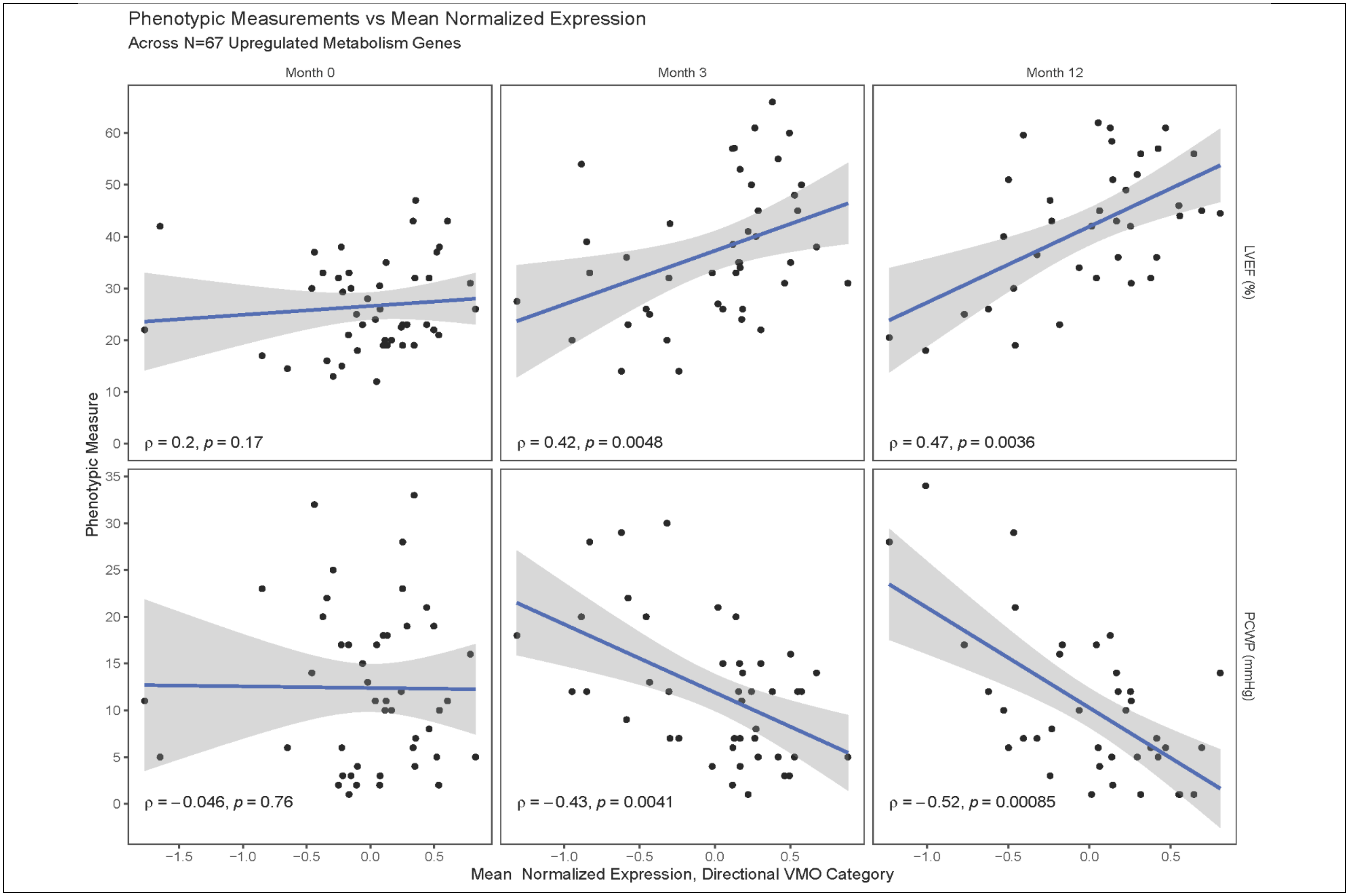
Phenotypic Measurements vs Mean Normalized Expression
Across N=67 Upregulated Metabolism Genes
Month 0
Month 3
Month 12
ρ = 0.2, p = 0.17
ρ = 0.42, p = 0.0048
ρ = 0.47, p = 0.0036
ρ = −0.046, p = 0.76
ρ = −0.43, p = 0.0041
ρ = −0.52, p = 0.00085
LVEF (%)
PCWP (mmHg)
Phenotypic Measure
Mean Normalized Expression, Directional VMO Category

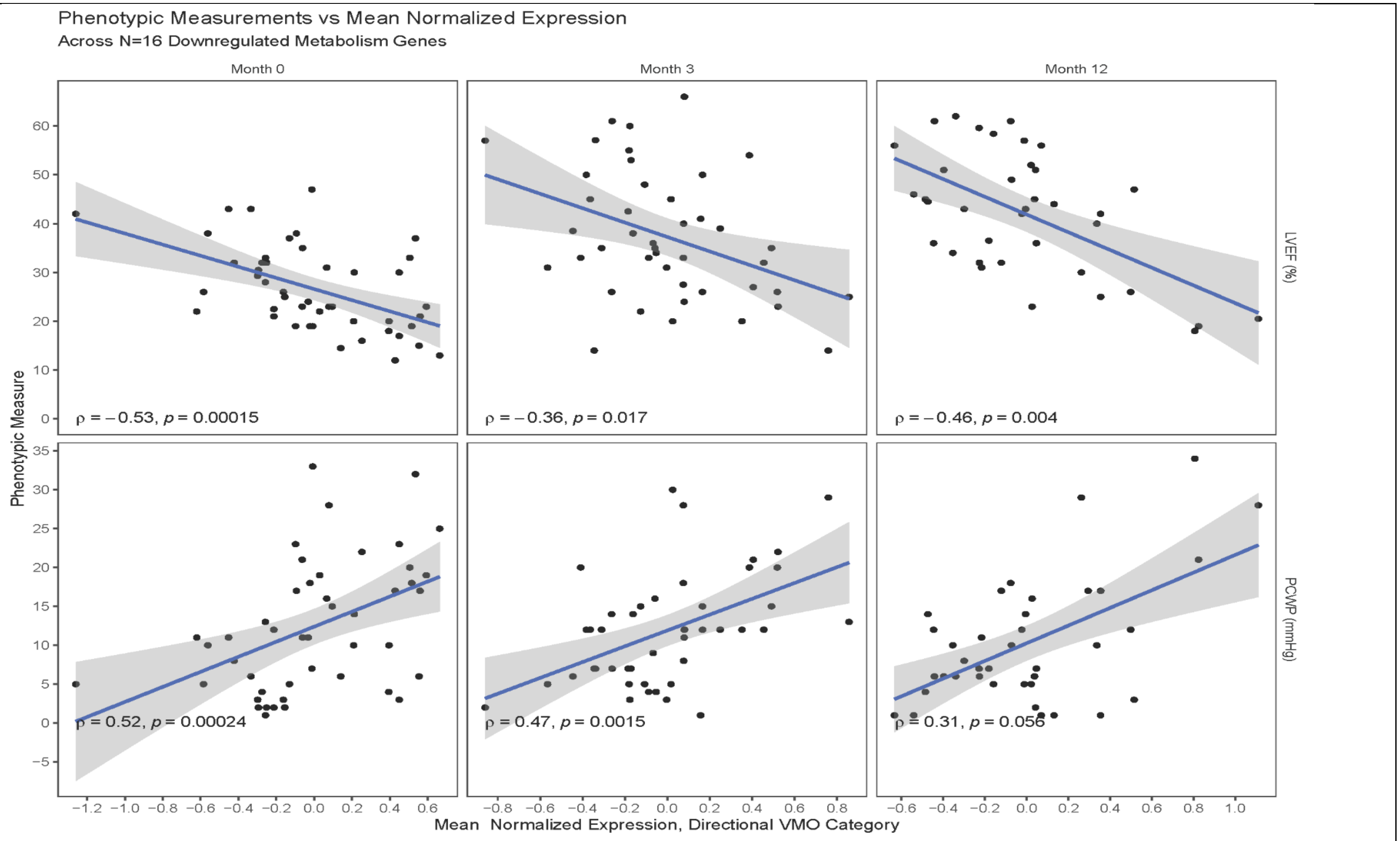

Figure S8. A. Metabolism genes that were upregulated (N=67) in Responders in the LOCF analysis, plotted for all 46 subjects as normalized mRNA abundance vs. either LVEF or PWP (PCWP) measurements. **B.** Metabolism Genes that were downregulated (N=16) in Responders, vs. LVEF or PWP (PCWP). In the inserts ρ= Rho, *p* = P value. Abundance of mRNA is Log2 transformed flourescence intensity Z transformed to a normal scale. For purposes of presentation clarity these plots consist of averaged mRNA abundance for the individual genes in the Metabolism category that are then subjected to Spearman's rank correlation for Rho calculation, as opposed to first generating Rho values for each individual gene's mRNA abundance vs. phenotypic measurement followed by averaging the Rho values as done for Z_p generation.

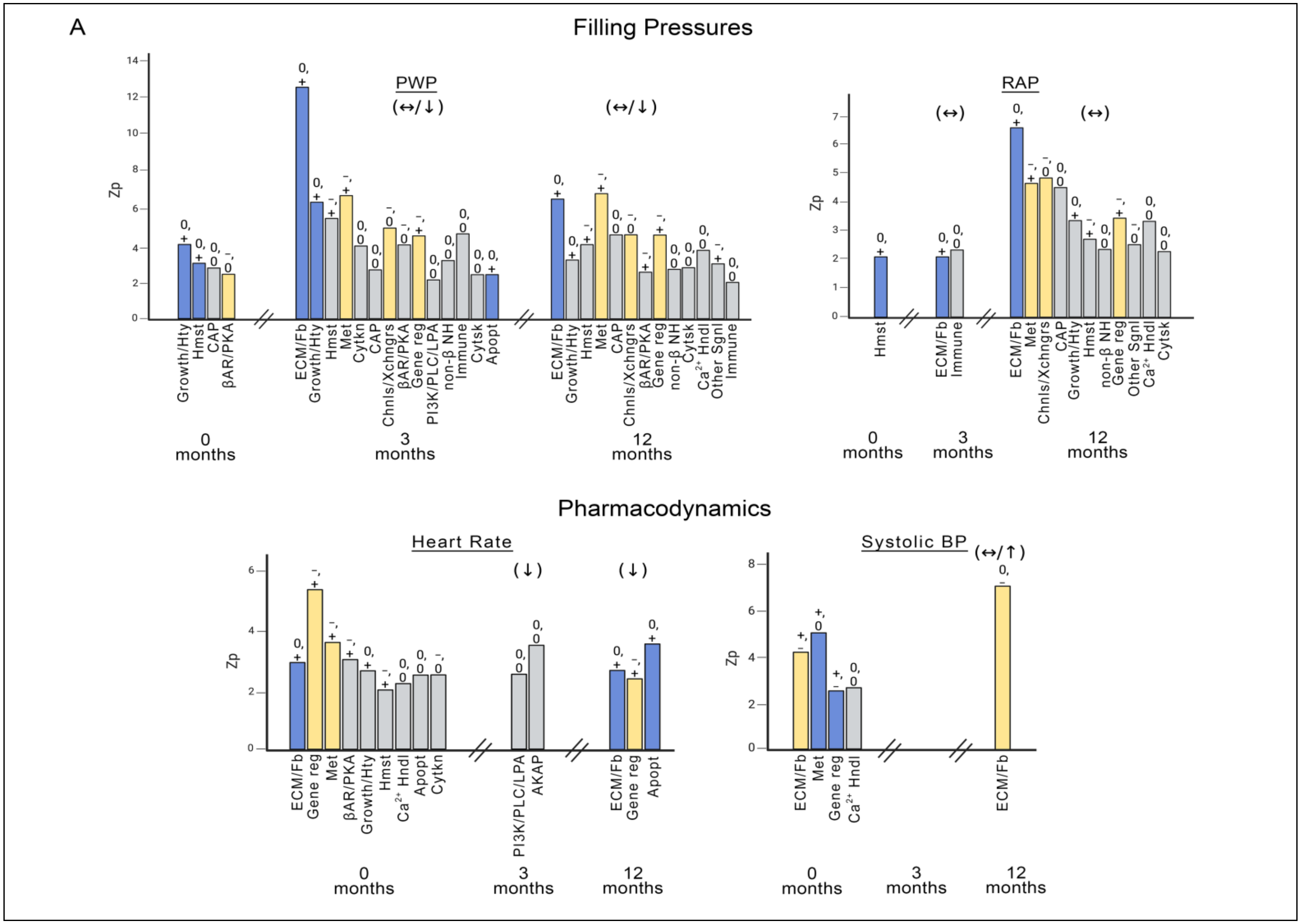
A
Filling Pressures
PWP
(↔/↓)
(↔/↓)
RAP
(↔)
(↔)
Zp
0 months
3 months
12 months
Pharmacodynamics
Heart Rate
(↓)
(↓)
Systolic BP
(↔/↑)
ECM/Fb
Growth/Hty
Hmst
Met
Cytkn
CAP
Chnls/Xchngrs
βAR/PKA
Gene reg
PI3K/PLC/LPA
non-β NH
Immune
Cytsk
Apopt
Other Sgnl
AKAP

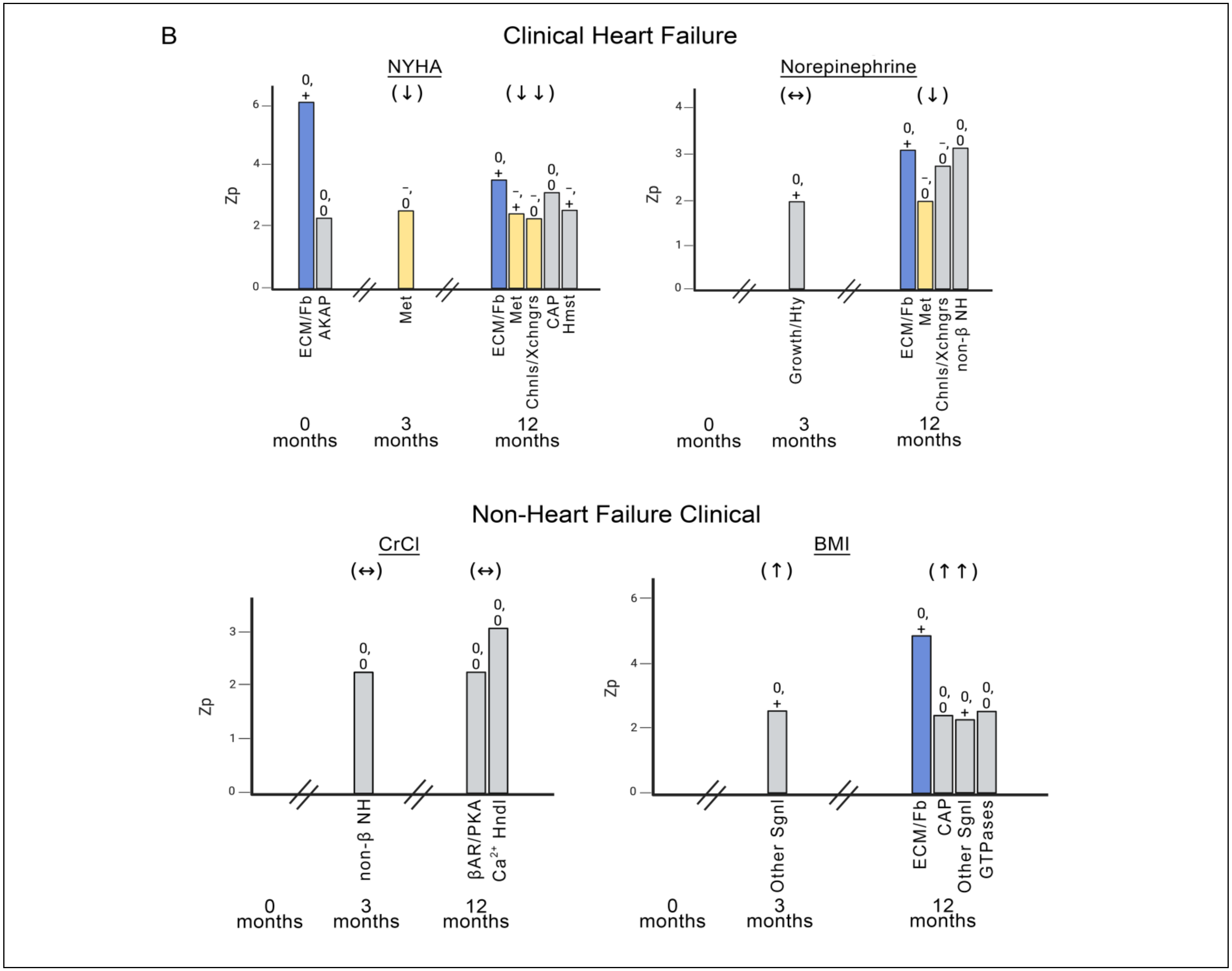

B
Clinical Heart Failure
NYHA
(↓)
(↓↓)
Zp
ECM/Fb
AKAP
Met
Chnls/Xchngrs
CAP
Hmst
0 months
3 months
12 months
Norepinephrine
(↔)
(↓)
Growth/Hty
non-β NH
Non-Heart Failure Clinical
CrCl
(↔)
βAR/PKA
Ca²⁺ Hndl
BMI
(↑)
(↑↑)
Other Sgnl
GTPases

Figure S9. Temporal pattern of mRNA abundance-phenotypic relationships, **A.** ventricular filling pressures and pharmacodynamics, **B.** clinical parameters. N=46 human nonischemic dilated cardiomyopathy patients at Baseline (Month 0, N=46) and with 3 months (N=46) and 12 months (N=39) of β-blocker treatment. Y axes are Z_p values ≥1.96 from nonparametric permutation testing of average Spearman's rank correlation Rho values of β_1-Gene Signaling Network (β_1-GSN) Ventricular Myocardial Ontology (VMO) categories (Table 2, Figure 3) vs. non-β_1-GSN VMO controls at Months 0, 3 and 12. Bars are color coded as: Blue, direct relationship of phenotypic measurement with net (including upregulated and downregulated genes) RNA expression; Yellow, inverse relationship of net mRNA abundance changes with phenotypic measure; Gray, no statistically significant relationship of net mRNA expression and phenotype measurement. The designations above the bars are: 1st entry is for upregulated genes, 2nd is for downregulated; (+) = direct directional correlation with the phenotypic measure, (−) = inverse directional correlation, 0 = no directional correlation.

11.0 Funding Sources

This work was supported by: National Heart, Lung, and Blood Institute Grants HL-07160 (awarded to SBL), 2R01 HL-48013 (awarded to MRB), 1K01HL-088708-01 (awarded to CCS), HL-051239 (awarded to JDP); American Heart Association grant 16SFRN31420008 (awarded to MRB); and ARCA biopharma, Westminster, CO.
